# Supplementary material for: New Pyrroline Isolated from Antarctic Krill-Derived Actinomycetes Nocardiopsis sp. LX-1 Combining with Molecular Networking
Source: Mar Drugs. 2023 Feb 15;21(2):127. doi: 10.3390/md21020127 (PMC9967698; doi:10.3390/md21020127)
Supplement: Supplementary file 1 [file marinedrugs-21-00127-s001.zip › marinedrugs-2101337-supplementary.pdf]

Supplementary material: New Pyrroline Isolated from Antarctic Krill-Derived Actinomycetes *Nocardiopsis* LX-1 Combining with Molecular Networking

Ting Shi, Yan-Jing Li, Ze-Min Wang, Yi-Fei Wang, Bo Wang and Da-Yong Shi

Figure S1. ^{1}H NMR spectrum of compound **1** (CD_3OD).
Figure S2. ^{13}C NMR spectrum of compound **1** (CD_3OD).
Figure S3. HSQC spectrum of compound **1** (CD_3OD).
Figure S4. COSY spectrum of compound **1** (CD_3OD).
Figure S5. HMBC spectrum of compound **1** (CD_3OD).
Figure S6. NOESY spectrum of compound **1** (CD_3OD).
Figure S7. HR-ESI$^-$-MS spectrum of compound **1**.
Figure S8. HR-ESI$^+$-MS spectrum of compound **1**.
Figure S9. IR spectrum of compound **1**.
Figure S10. ^{1}H NMR spectrum of compound **2** (DMSO-d_6).
Figure S11. ^{13}C NMR spectrum of compound **2** (DMSO-d_6).
Figure S12. HR-ESI$^-$-MS spectrum of compound **2**.
Figure S13. HR-ESI$^+$-MS spectrum of compound **2**.
Figure S14. ^{1}H NMR spectrum of compound **3** ($CDCl_3$).
Figure S15. ^{13}C NMR spectrum of compound **3** ($CDCl_3$).
Figure S16. HR-ESI$^-$-MS spectrum of compound **3**.
Figure S17. HR-ESI$^+$-MS spectrum of compound **3**.
Figure S18. ^{1}H NMR spectrum of compound **4** ($CDCl_3$).
Figure S19. ^{13}C NMR spectrum of compound **4** ($CDCl_3$).
Figure S20. HR-ESI$^-$-MS spectrum of compound **4**.
Figure S21. HR-ESI$^+$-MS spectrum of compound **4**.
Figure S22. ^{1}H NMR spectrum of compound **5** (CD_3OD).
Figure S23. ^{13}C NMR spectrum of compound **5** (CD_3OD).
Figure S24. HR-ESI$^-$-MS spectrum of compound **5**.
Figure S25. HR-ESI$^+$-MS spectrum of compound **5**.
Figure S26. ^{1}H NMR spectrum of compound **6** ($CDCl_3$).
Figure S27. ^{13}C NMR spectrum of compound **6** ($CDCl_3$).
Figure S28. HR-ESI$^-$-MS spectrum of compound **6**.
Figure S29. HR-ESI$^+$-MS spectrum of compound **6**.
Figure S30. ^{1}H NMR spectrum of compound **7** ($CDCl_3$).
Figure S31. ^{13}C NMR spectrum of compound **7** ($CDCl_3$).
Figure S32. HR-ESI$^-$-MS spectrum of compound **7**.
Figure S33. HR-ESI$^+$-MS spectrum of compound **7**.
Figure S34. ^{1}H NMR spectrum of compound **8** (CD_3OD).
Figure S35. ^{13}C NMR spectrum of compound **8** (CD_3OD).
Figure S36. HR-ESI$^-$-MS spectrum of compound **8**.
Figure S37. HR-ESI$^+$-MS spectrum of compound **8**.

Figure S38. ^{1}H NMR spectrum of compound **9** (DMSO-d_6).
Figure S39. ^{13}C NMR spectrum of compound **9** (DMSO-d_6).
Figure S40. HR-ESI^{-}-MS spectrum of compound **9**.
Figure S41. ^{1}H NMR spectrum of compound **10** (DMSO-d_6).
Figure S42. ^{13}C NMR spectrum of compound **10** (DMSO-d_6).
Figure S43. HR-ESI^{-}-MS spectrum of compound **10**.
Figure S44. HR-ESI^{+}-MS spectrum of compound **10**.
Figure S45. ^{1}H NMR spectrum of compound **11** (CD_3OD).
Figure S46. ^{13}C NMR spectrum of compound **11** (CD_3OD).
Figure S47. HR-APCI-MS spectrum of compound **11**.
Figure S48. ^{1}H NMR spectrum of compound **12** ($CDCl_3$).
Figure S49. ^{13}C NMR spectrum of compound **12** ($CDCl_3$).
Figure S50. HR-ESI^{-}-MS spectrum of compound **12**.
Figure S51. HR-ESI^{+}-MS spectrum of compound **12**.
Figure S52. MS/MS spectrum of compound **a** (black) compared with GNPS library spectrum (green).
Figure S53. MS/MS spectrum of compound **b** (black) compared with GNPS library spectrum (green).
Figure S54. MS/MS spectrum of compound **c** (black) compared with GNPS library spectrum (green).
Figure S55. MS/MS spectrum of compound **d** (black) compared with GNPS library spectrum (green).
Figure S56. MS/MS spectrum of compound **e** (black) compared with GNPS library spectrum (green).
Figure S57. MS/MS spectrum of compound **f** (black) compared with GNPS library spectrum (green).
Figure S58. MS/MS spectrum of compound **g** (black) compared with GNPS library spectrum (green).
Figure S59. MS/MS spectrum of compound **h** (black) compared with GNPS library spectrum (green).
Figure S60. MS/MS spectrum of compound **i** (black) compared with GNPS library spectrum (green).
Figure S61. MS/MS spectrum of compound **j** (black) compared with GNPS library spectrum (green).
Figure S62. MS/MS spectrum of compound **k** (black) compared with GNPS library spectrum (green).
Figure S63. MS/MS spectrum of compound **l** (black) compared with GNPS library spectrum (green).
Figure S64. MS/MS spectrum of compound **m** (black) compared with GNPS library spectrum (green).
Figure S65. MS/MS spectrum of compound **n** (black) compared with GNPS library spectrum (green).
Figure S66. MS/MS spectrum of compound **o** (black) compared with GNPS library spectrum (green).
Figure S67. MS/MS spectrum of compound **p** (black, $[M+H]^+$) compared with GNPS library spectrum (green).
Figure S68. MS/MS spectrum of compound **p** (black, $[M+Na]^+$) compared with GNPS library spectrum (green).
Table S1. NMR spectroscopic data (400/100 MHz) of daidzein (**2**) in DMSO-d_6.
Table S2. ^{1}H NMR spectroscopic data (400 MHz) of compounds **3**–**8** in $CDCl_3$.
Table S3. ^{13}C NMR spectroscopic data (100 MHz) of compounds **3**–**8** in $CDCl_3$.
Table S4. Specific OR of cyclic dipeptides **3**–**8** in MeOH.
Table S5. NMR spectroscopic data (400/100 MHz) of compounds **9**–**12**.

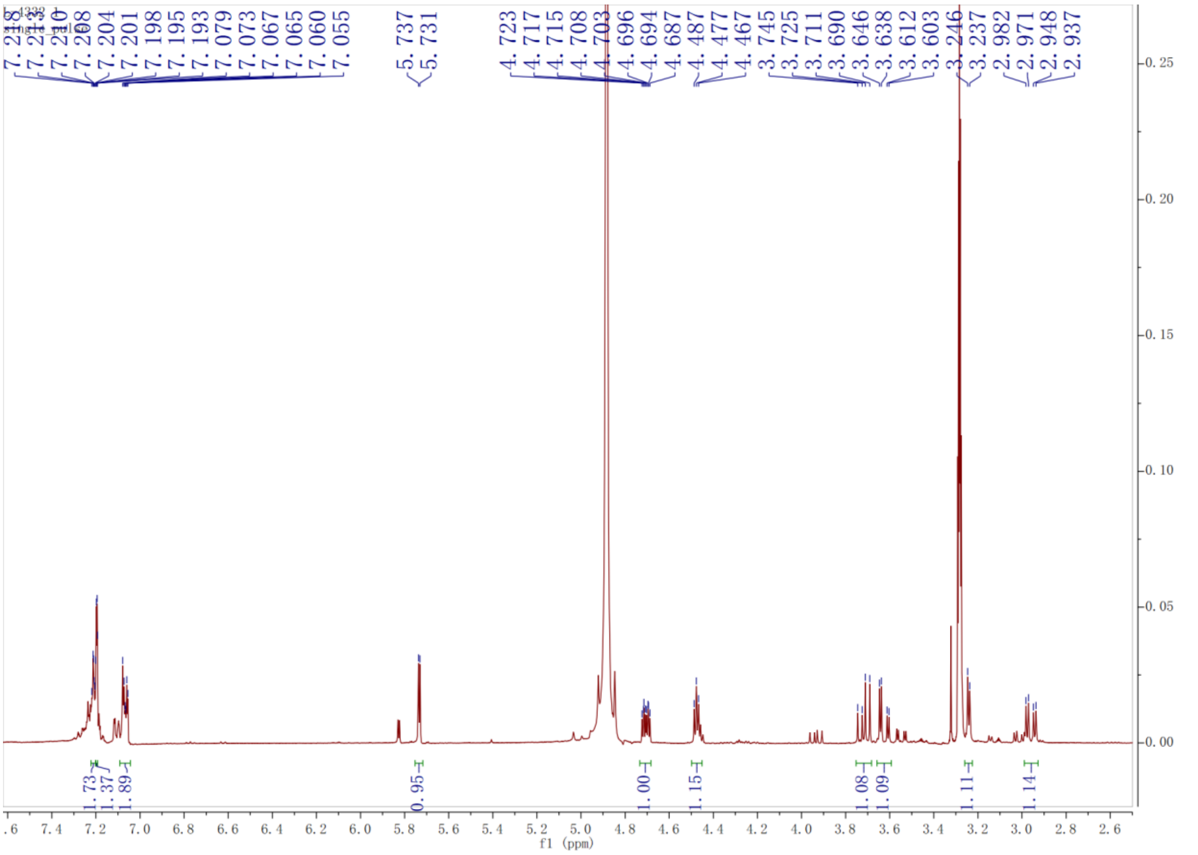

Figure S1. ^{1}H NMR spectrum of compound **1** (CD_3OD).

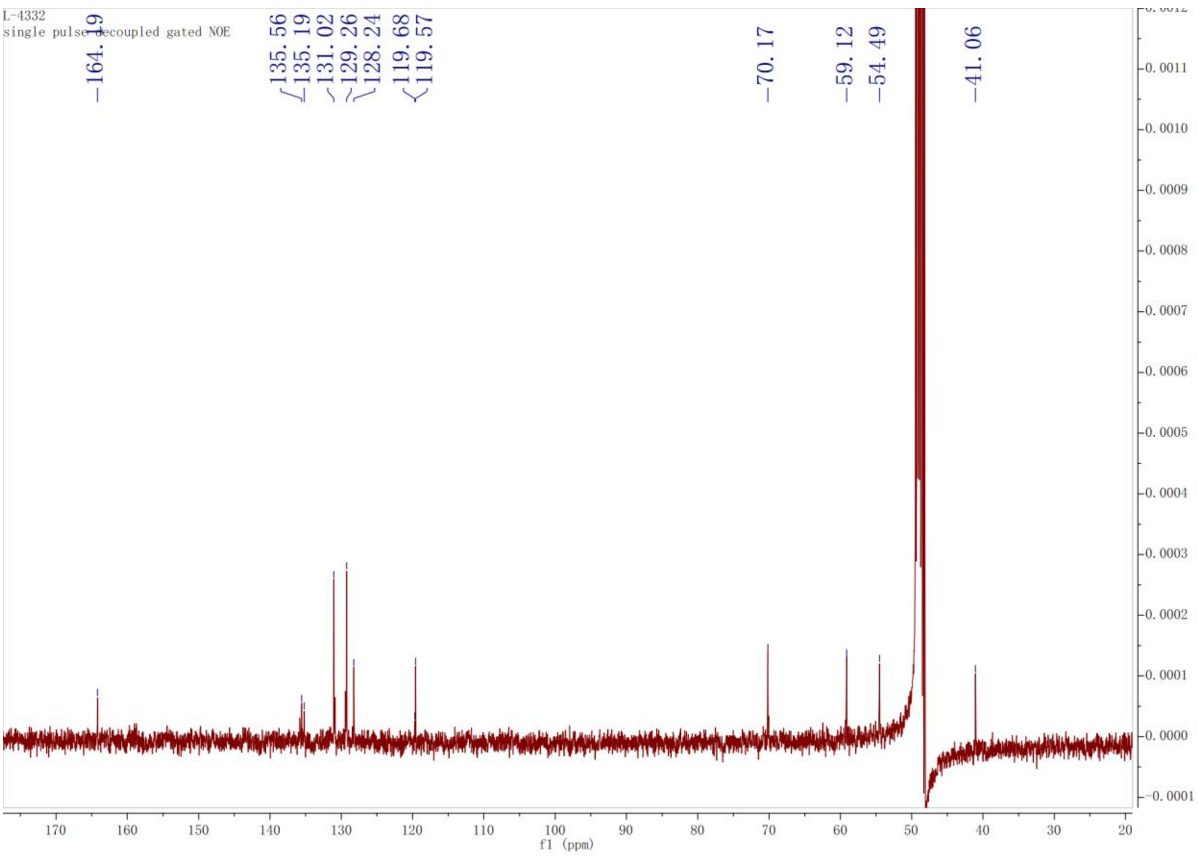

Figure S2. ^{13}C NMR spectrum of compound **1** (CD_3OD).

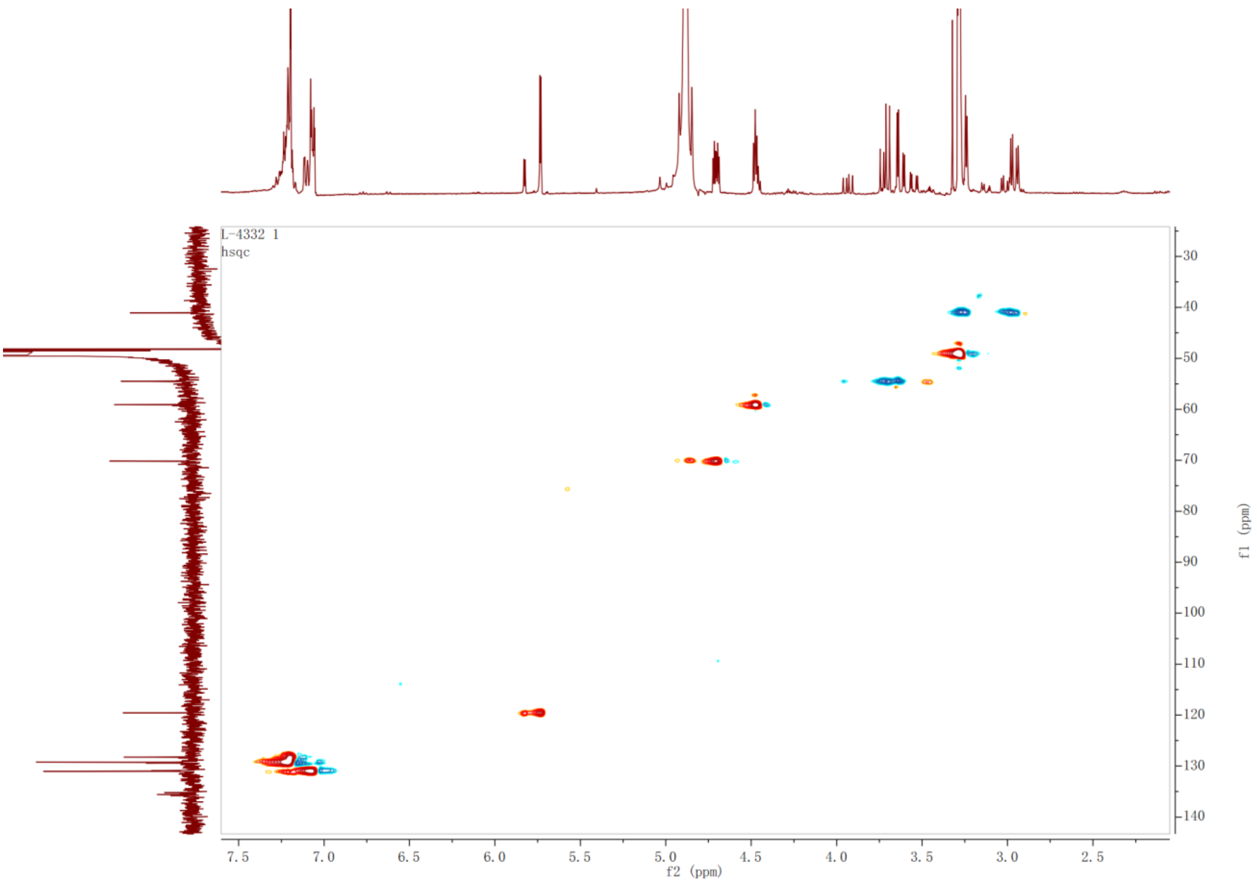

Figure S3. HSQC spectrum of compound **1** (CD_3OD).

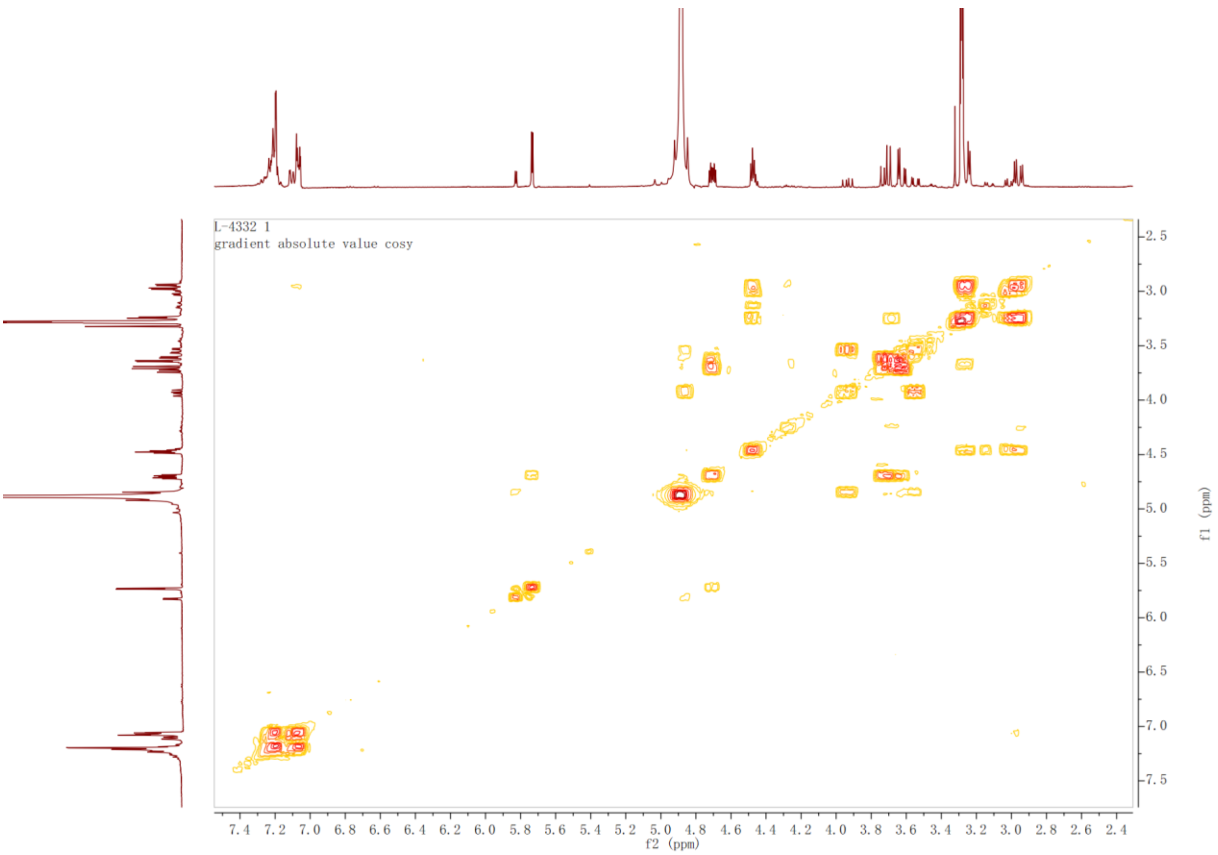

Figure S4. COSY spectrum of compound **1** (CD_3OD).

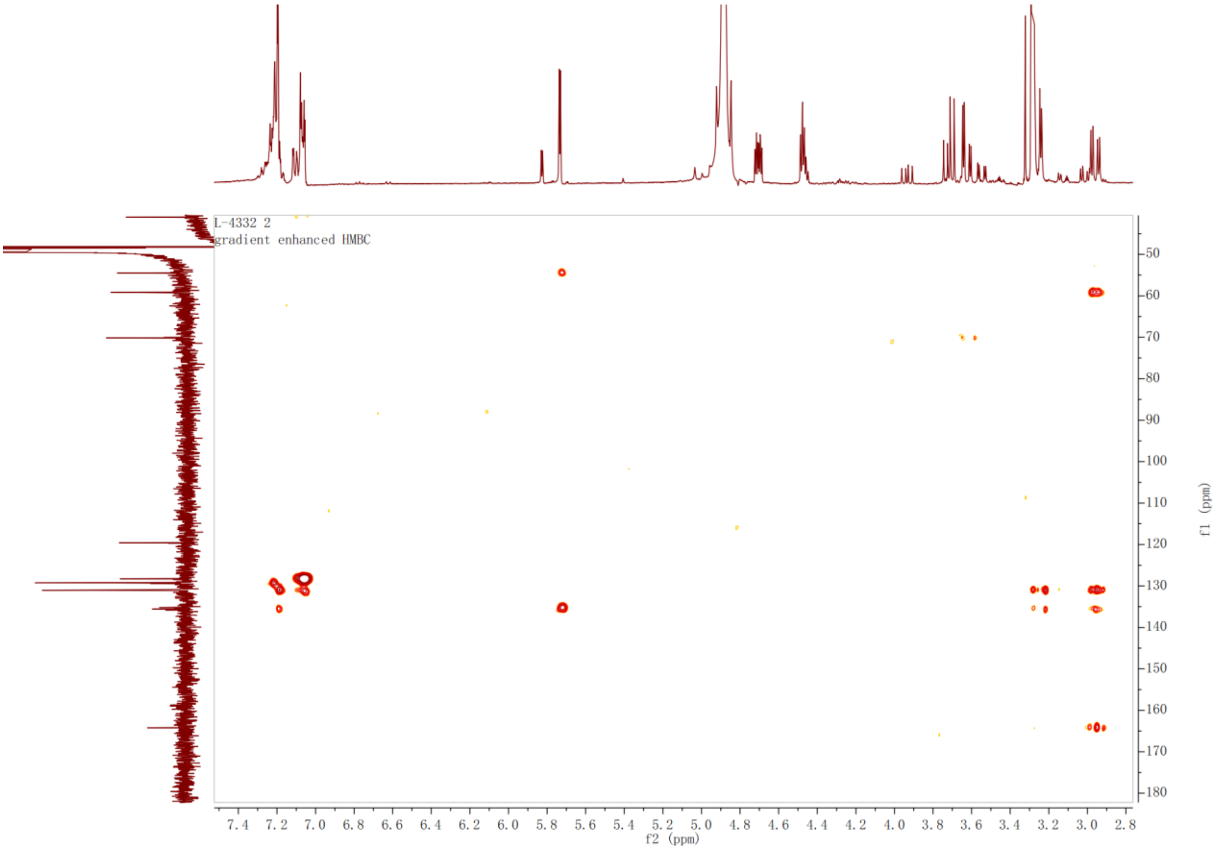

Figure S5. HMBC spectrum of compound **1** (CD_3OD).

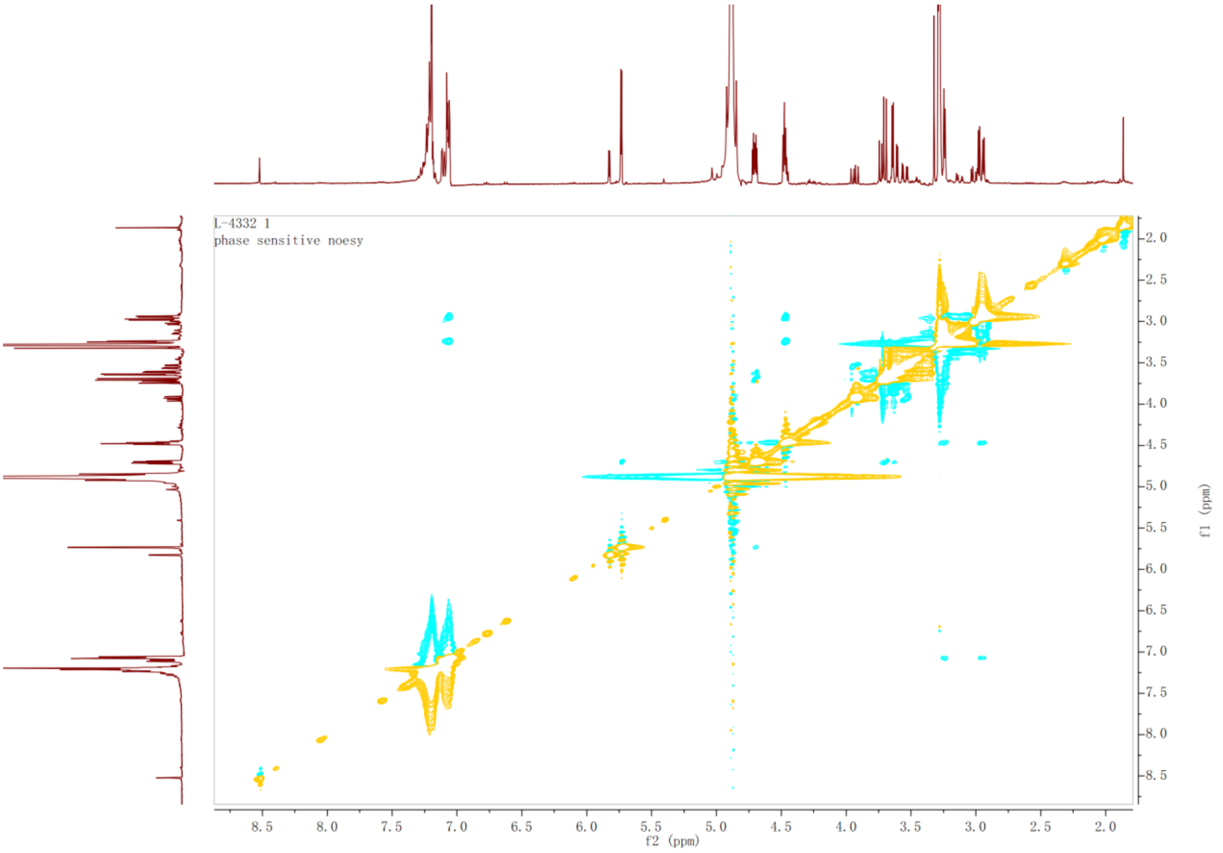

Figure S6. NOESY spectrum of compound **1** (CD_3OD).

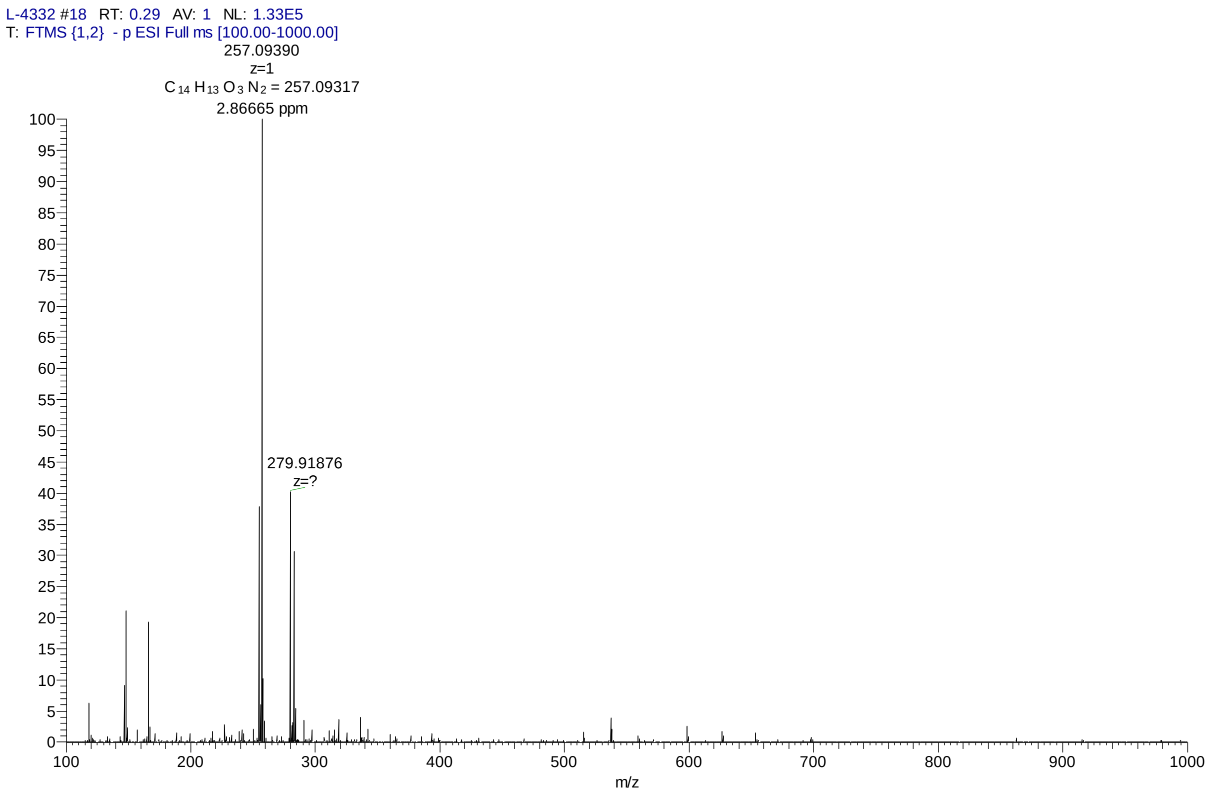

Figure S7. HR-ESI$^-$-MS spectrum of compound **1**.

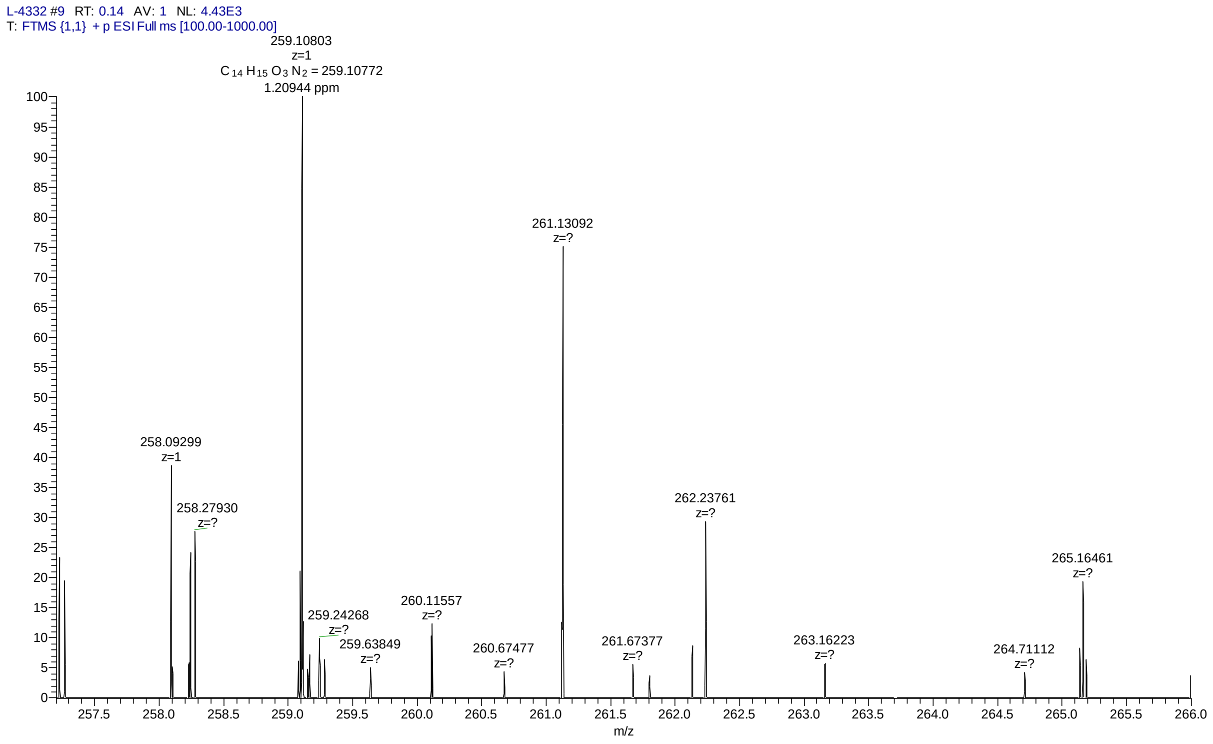

Figure S8. HR-ESI$^+$-MS spectrum of compound **1**.

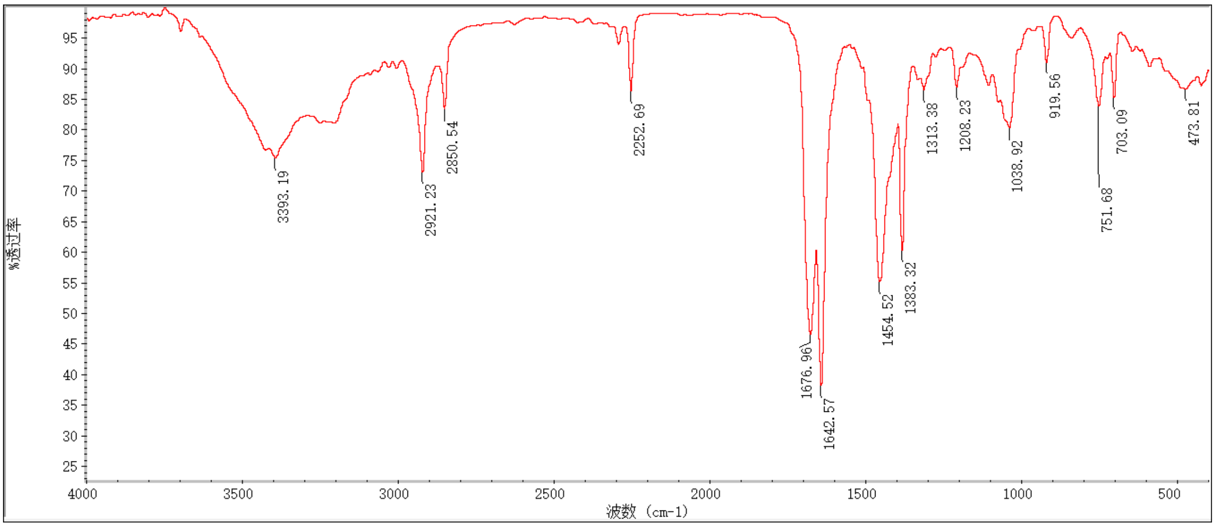

Figure S9. IR spectrum of compound **1**.

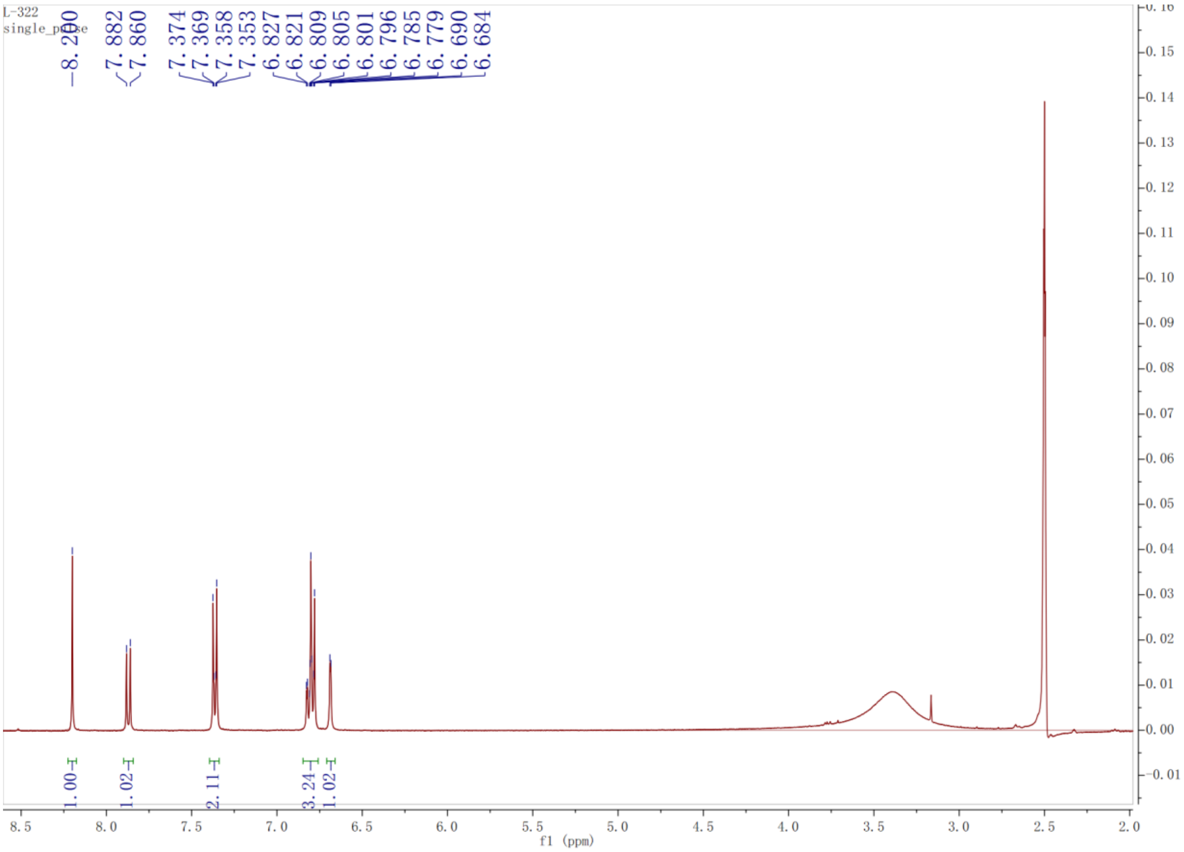

Figure S10. ^{1}H NMR spectrum of compound **2** (DMSO-d_6).

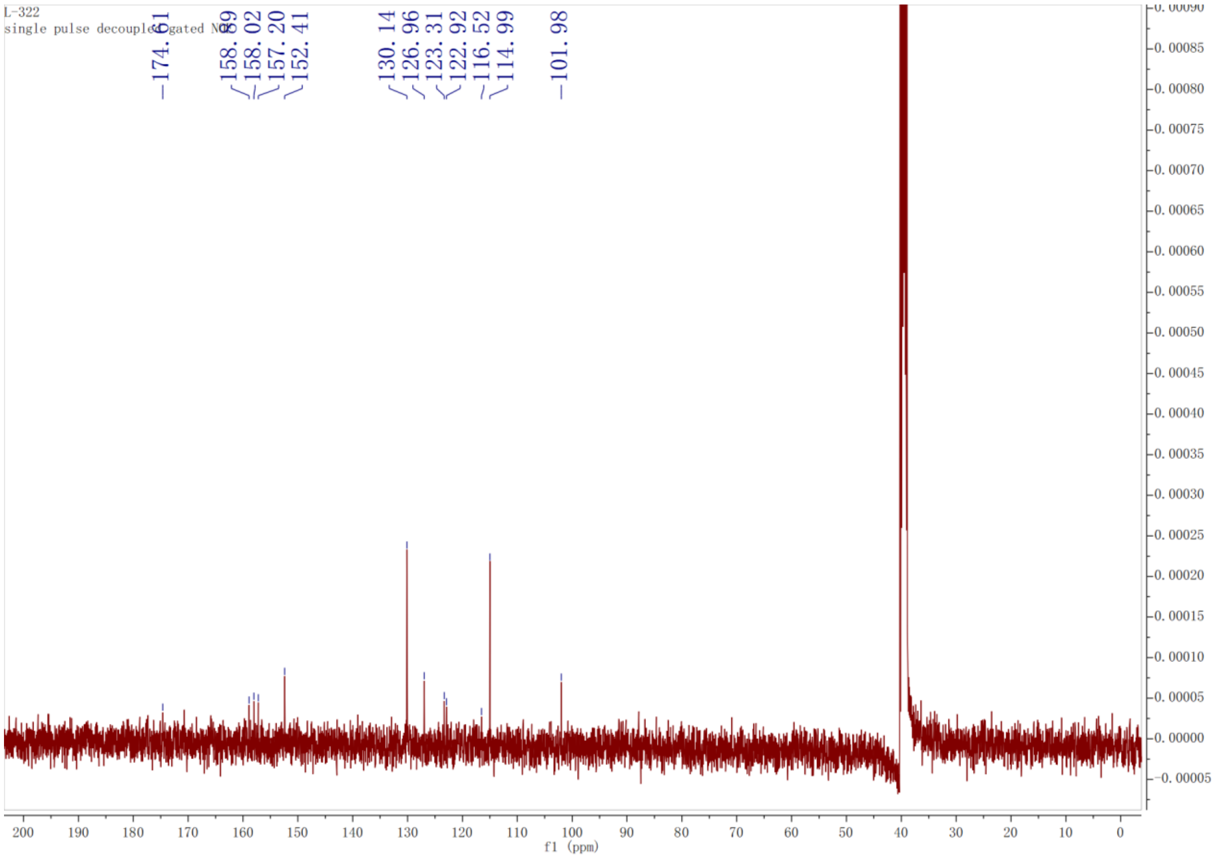

Figure S11. ^{13}C NMR spectrum of compound **2** (DMSO-d_6).

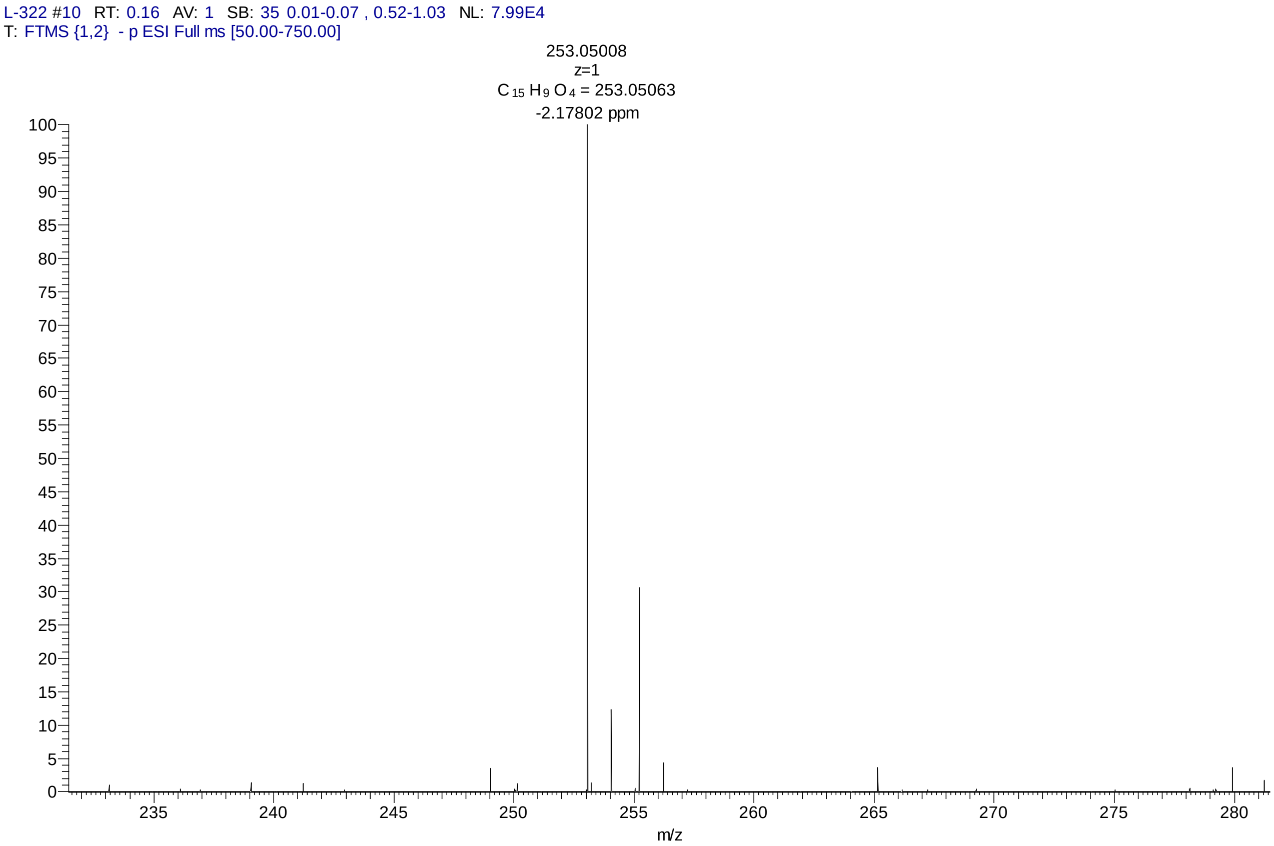

Figure S12. HR-ESI$^-$-MS spectrum of compound **2**.

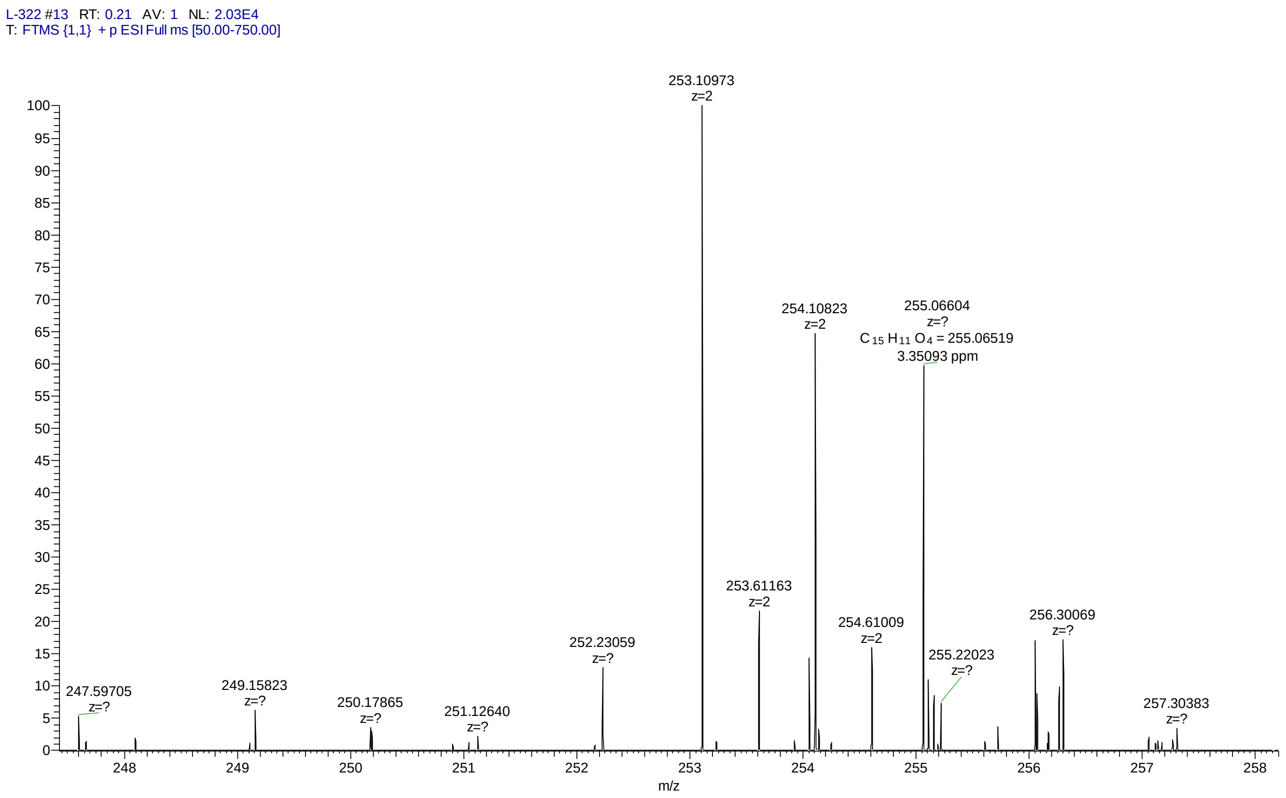

Figure S13. HR-ESI+-MS spectrum of compound **2**.

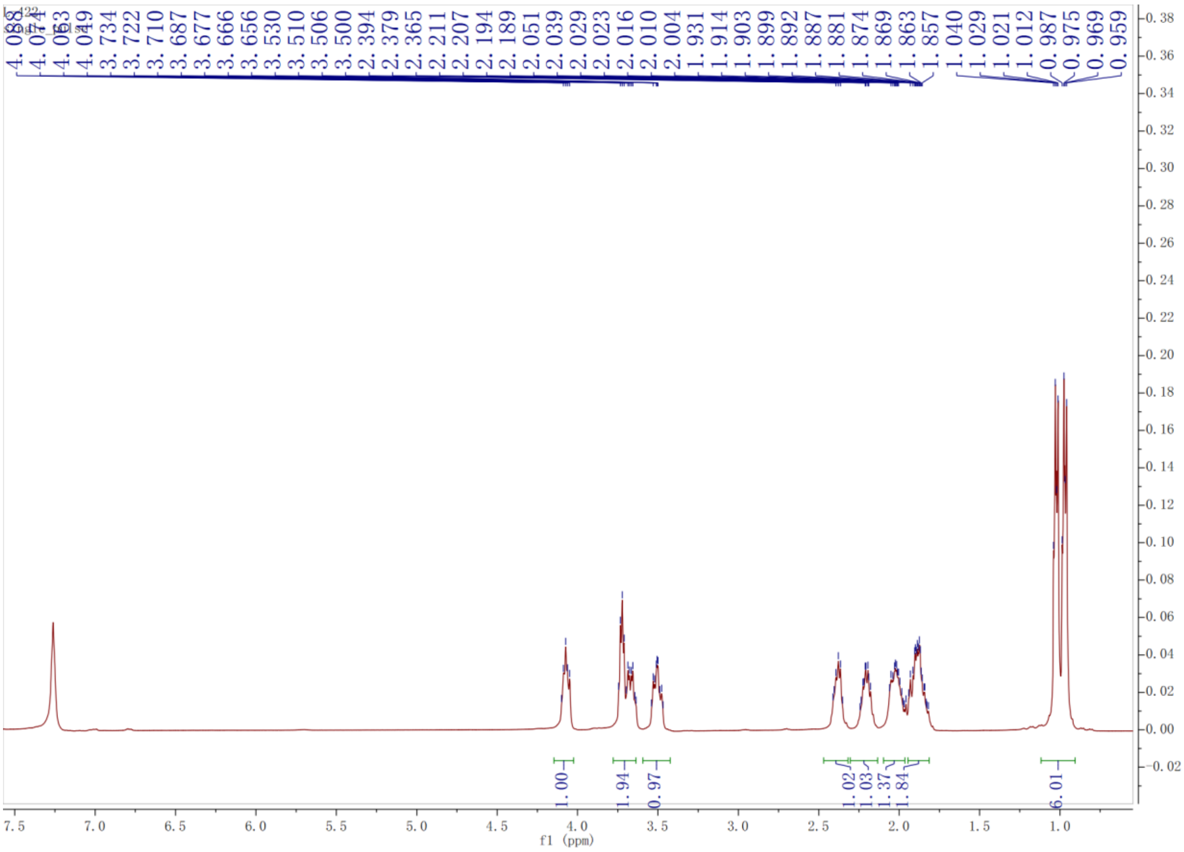

Figure S14. ^{1}H NMR spectrum of compound **3** ($CDCl_3$).

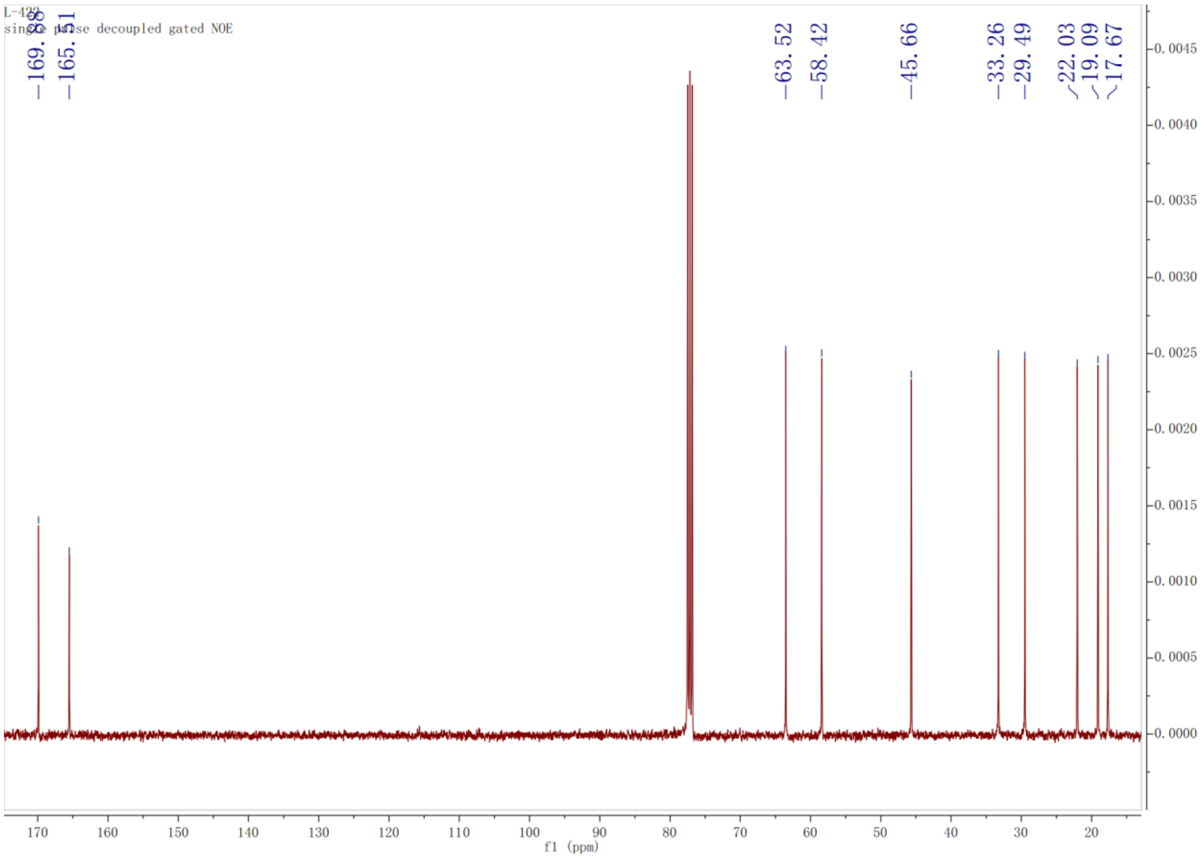

Figure S15. ^{13}C NMR spectrum of compound **3** ($CDCl_3$).

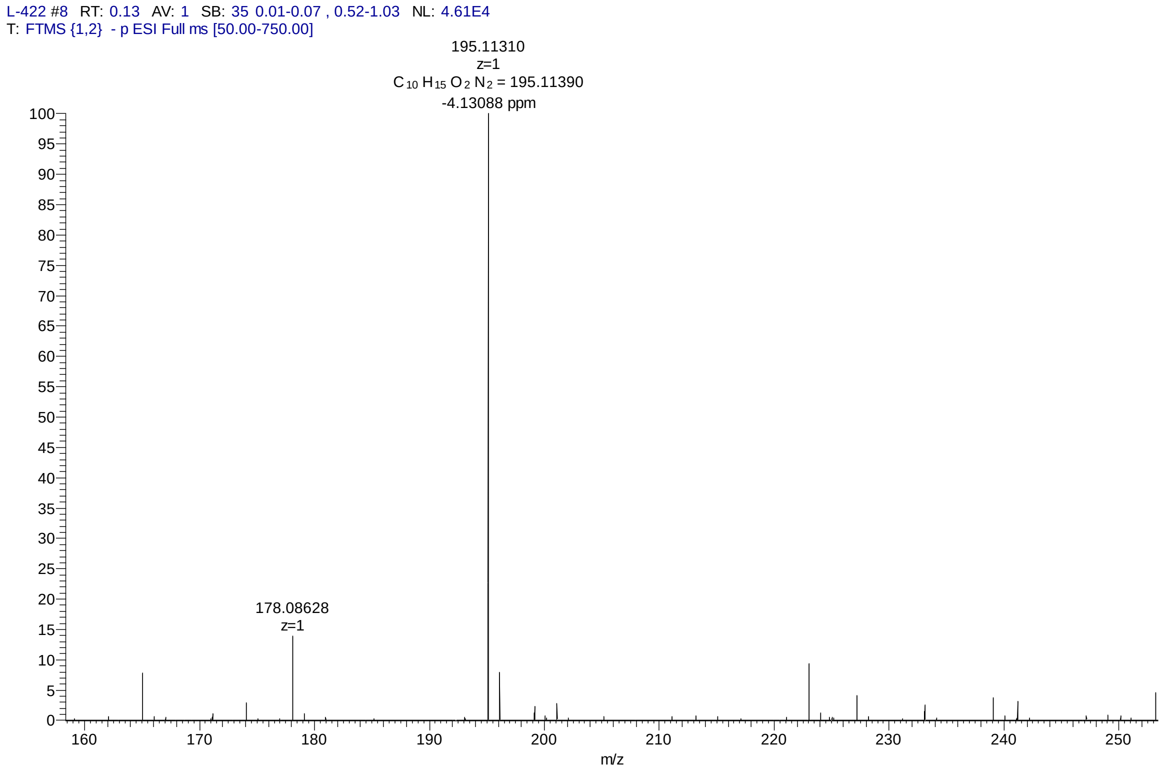

Figure S16. HR-ESI^{-}-MS spectrum of compound **3**.

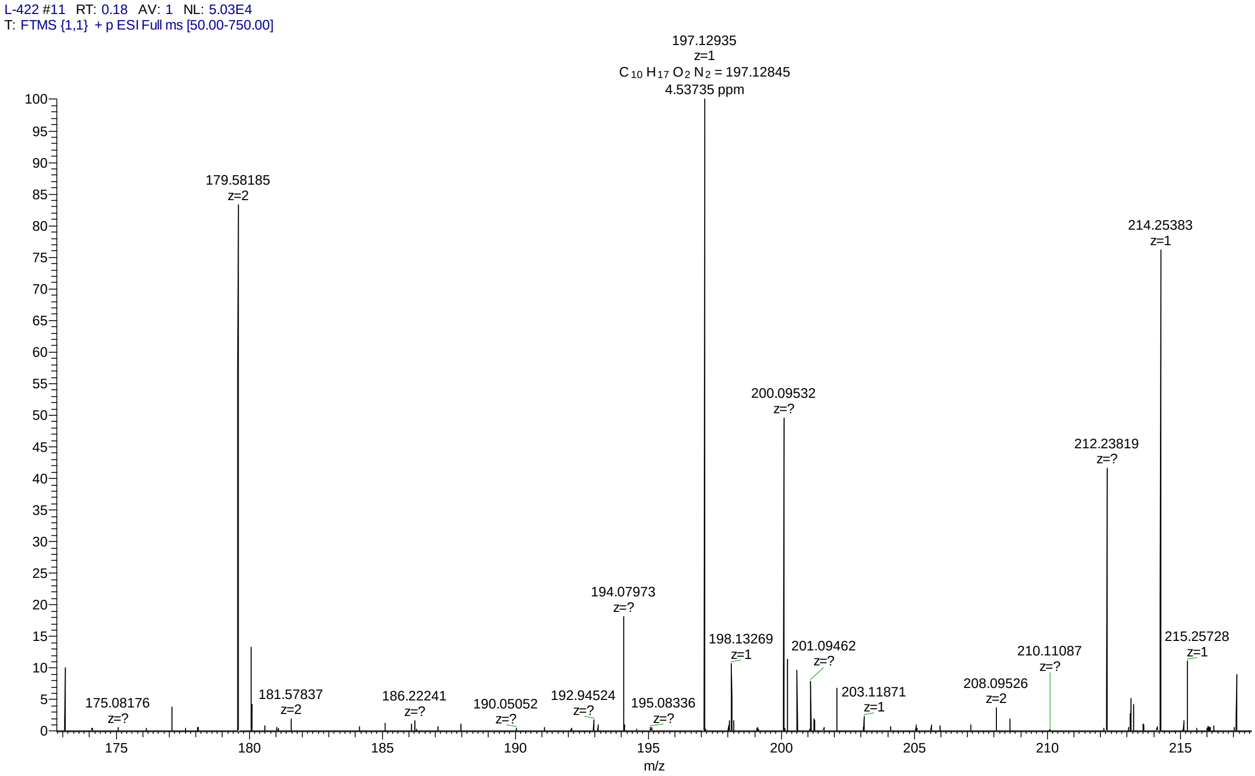

Figure S17. HR-ESI+-MS spectrum of compound **3**.

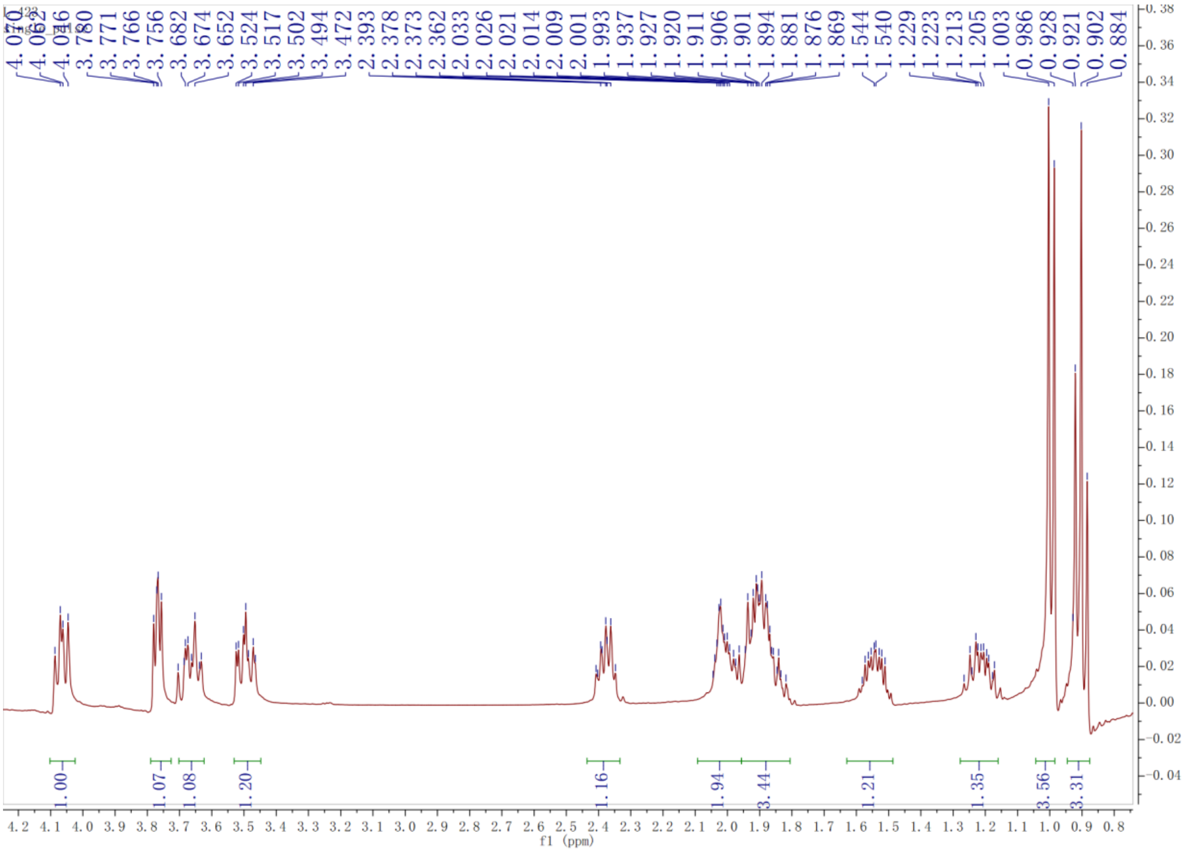

Figure S18. ^{1}H NMR spectrum of compound **4** ($CDCl_3$).

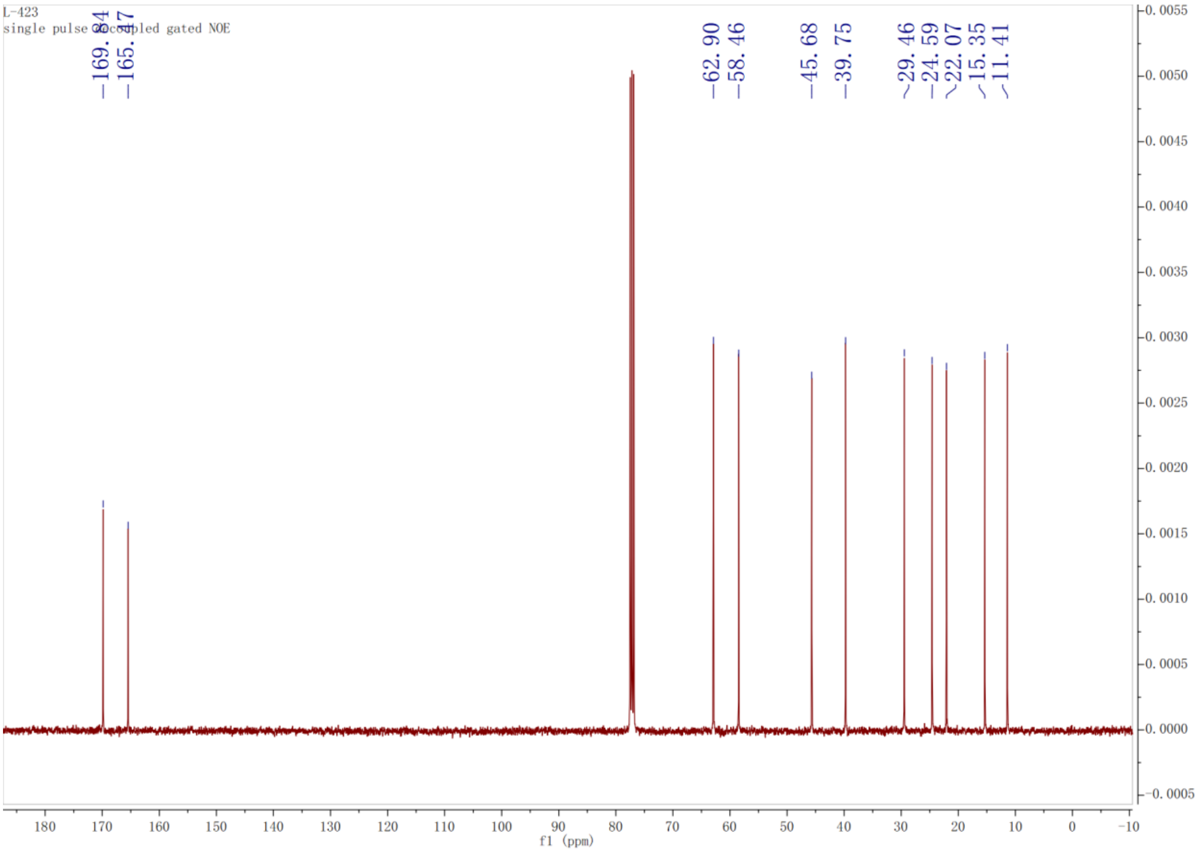

Figure S19. ^{13}C NMR spectrum of compound **4** ($CDCl_3$).

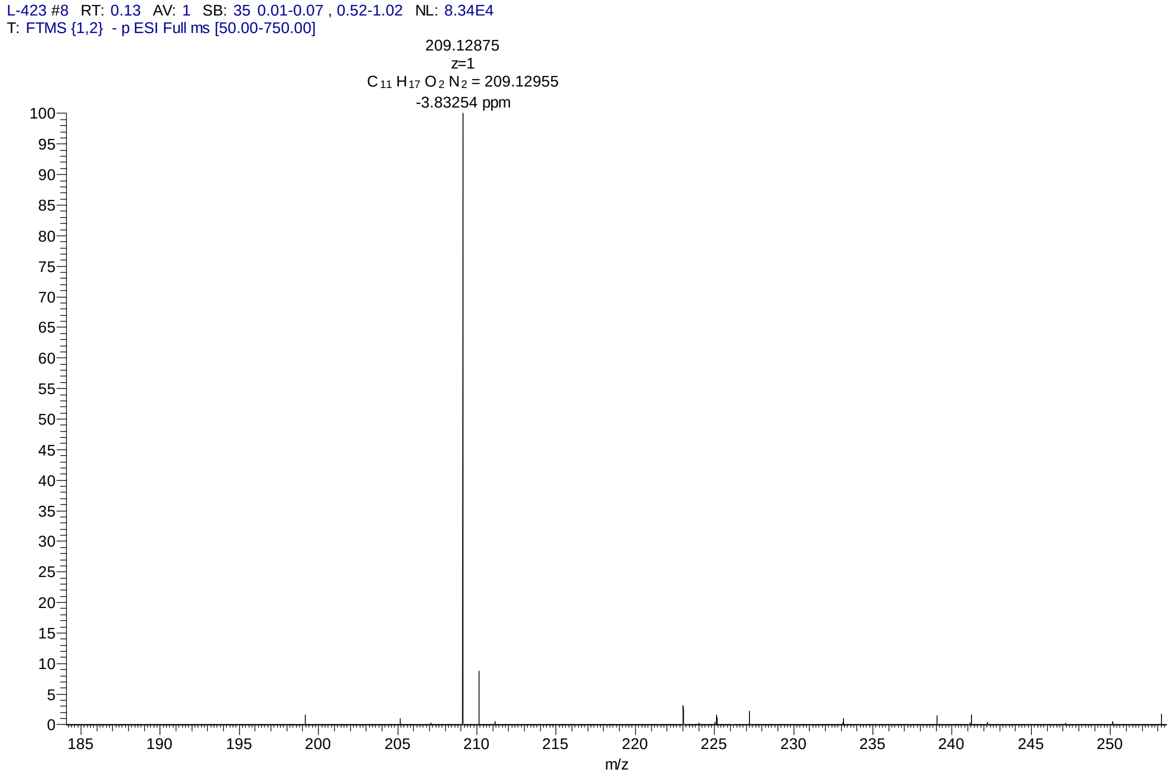

Figure S20. HR-ESI$^-$-MS spectrum of compound **4**.

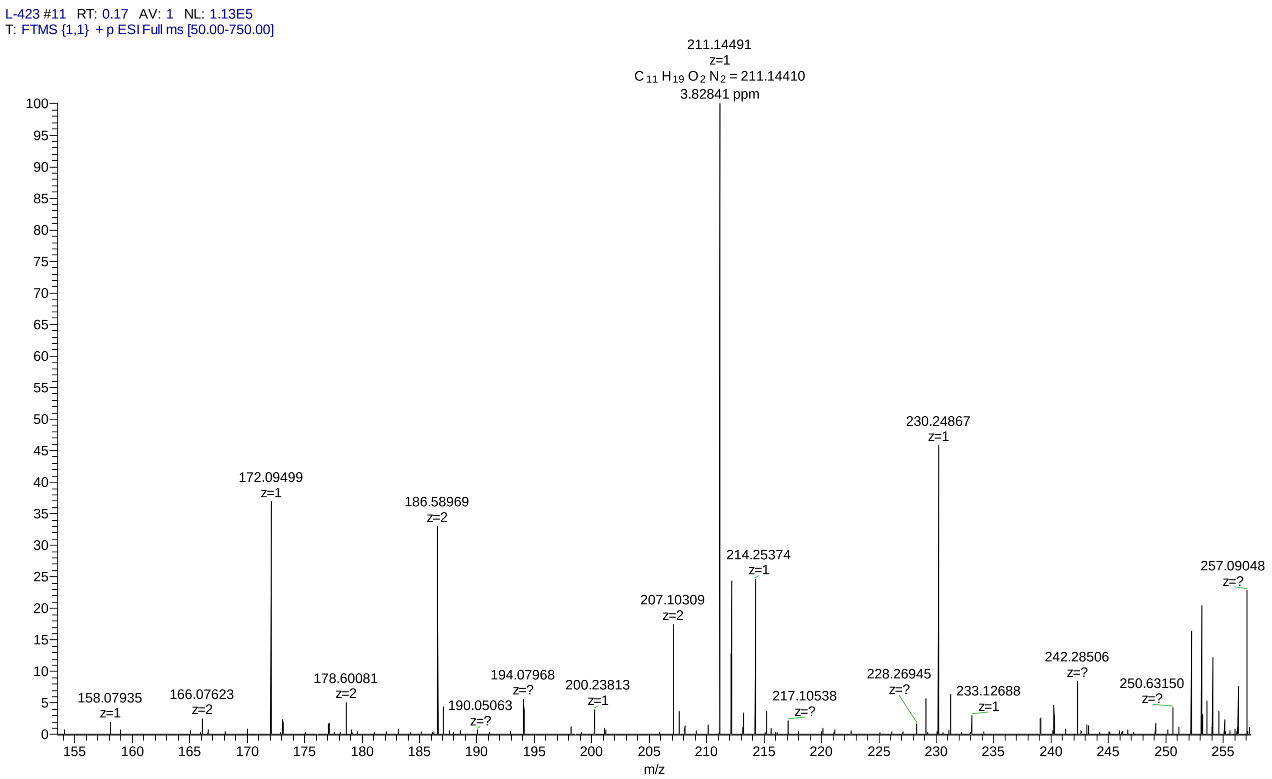

Figure S21. HR-ESI$^+$-MS spectrum of compound **4**.

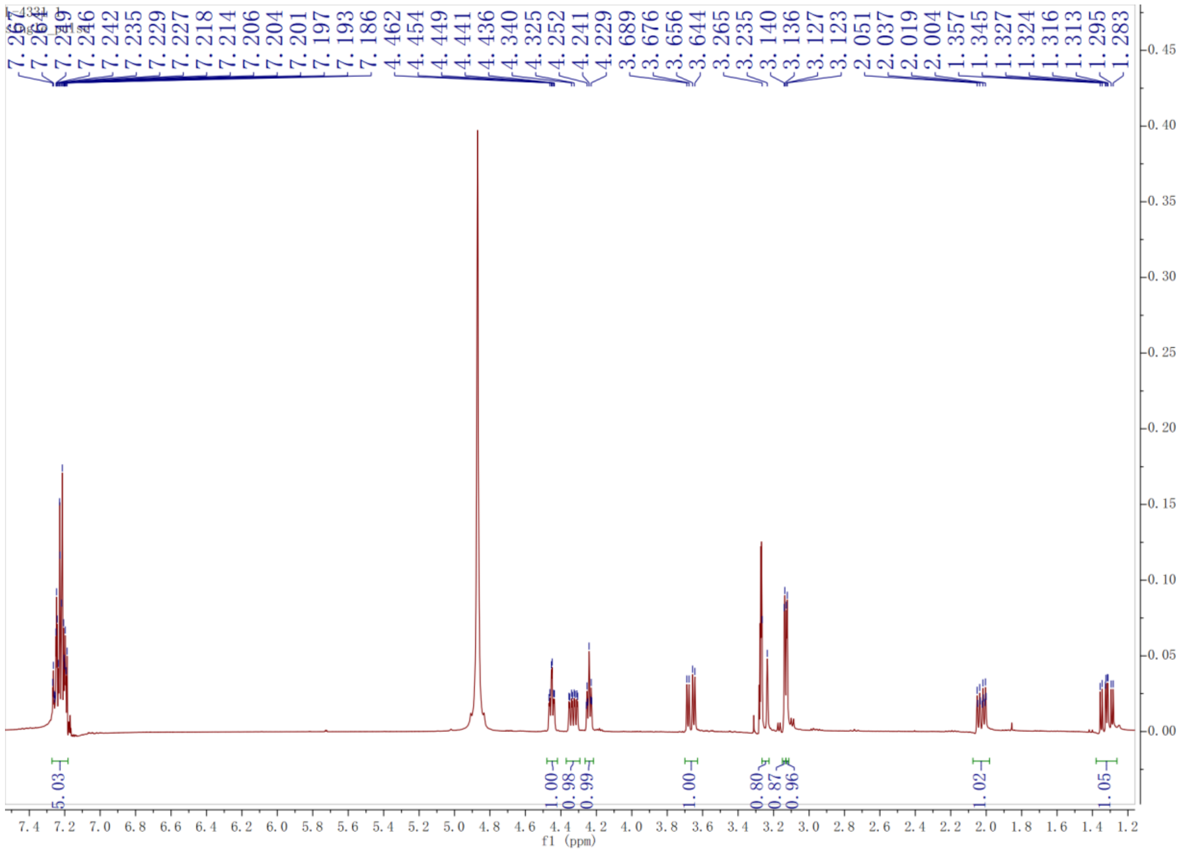

Figure S22. ^{1}H NMR spectrum of compound **5** (CD_3OD).

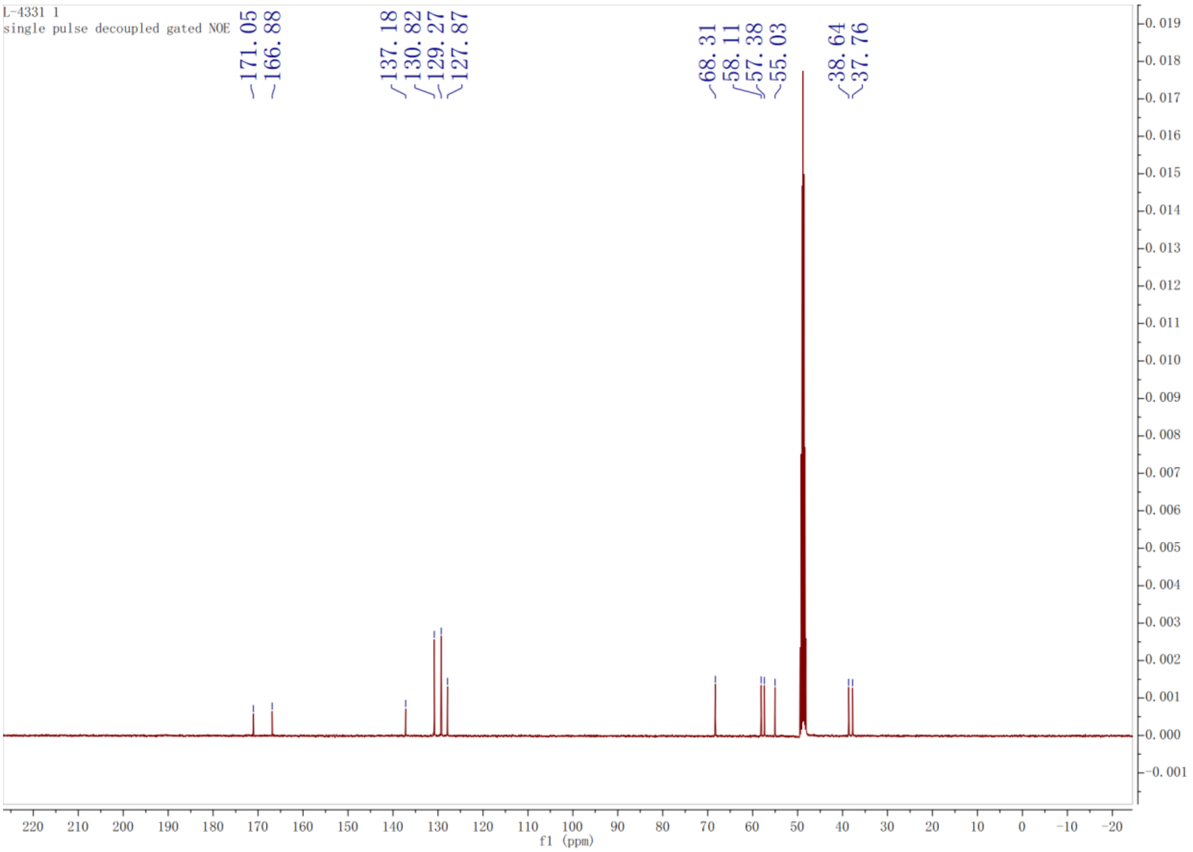

Figure S23. ^{13}C NMR spectrum of compound **5** (CD_3OD).

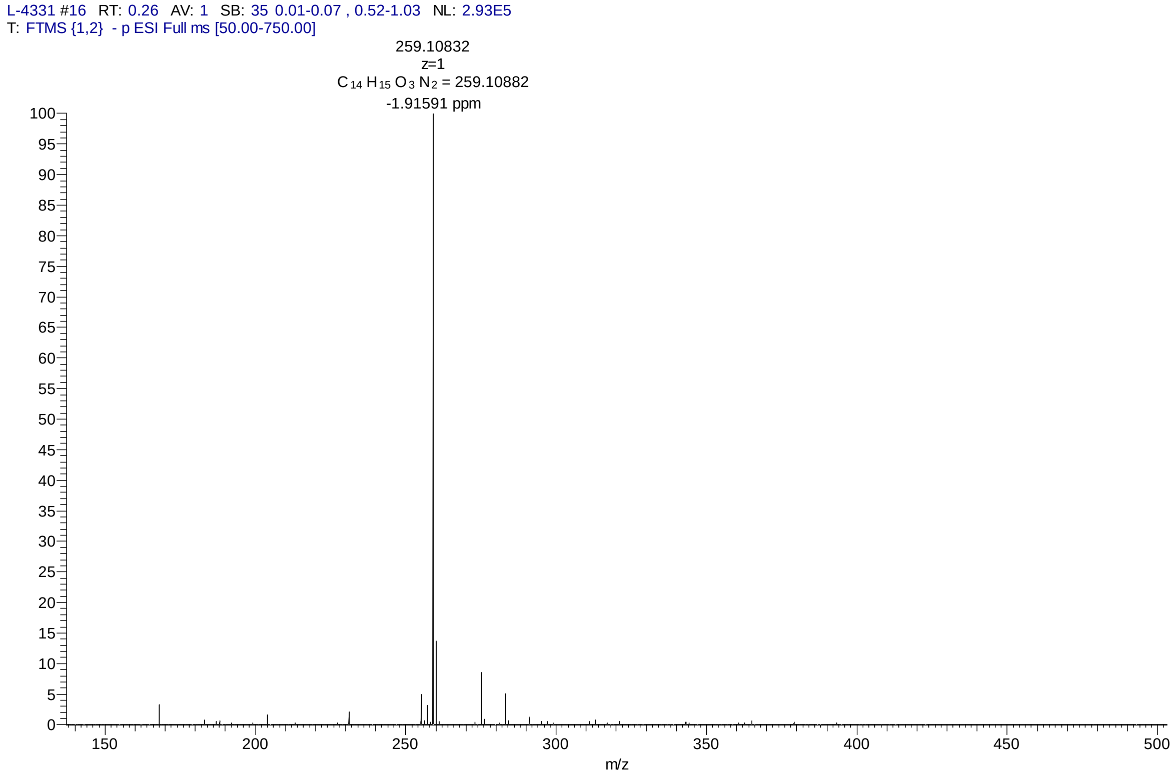

Figure S24. HR-ESI$^-$-MS spectrum of compound **5**.

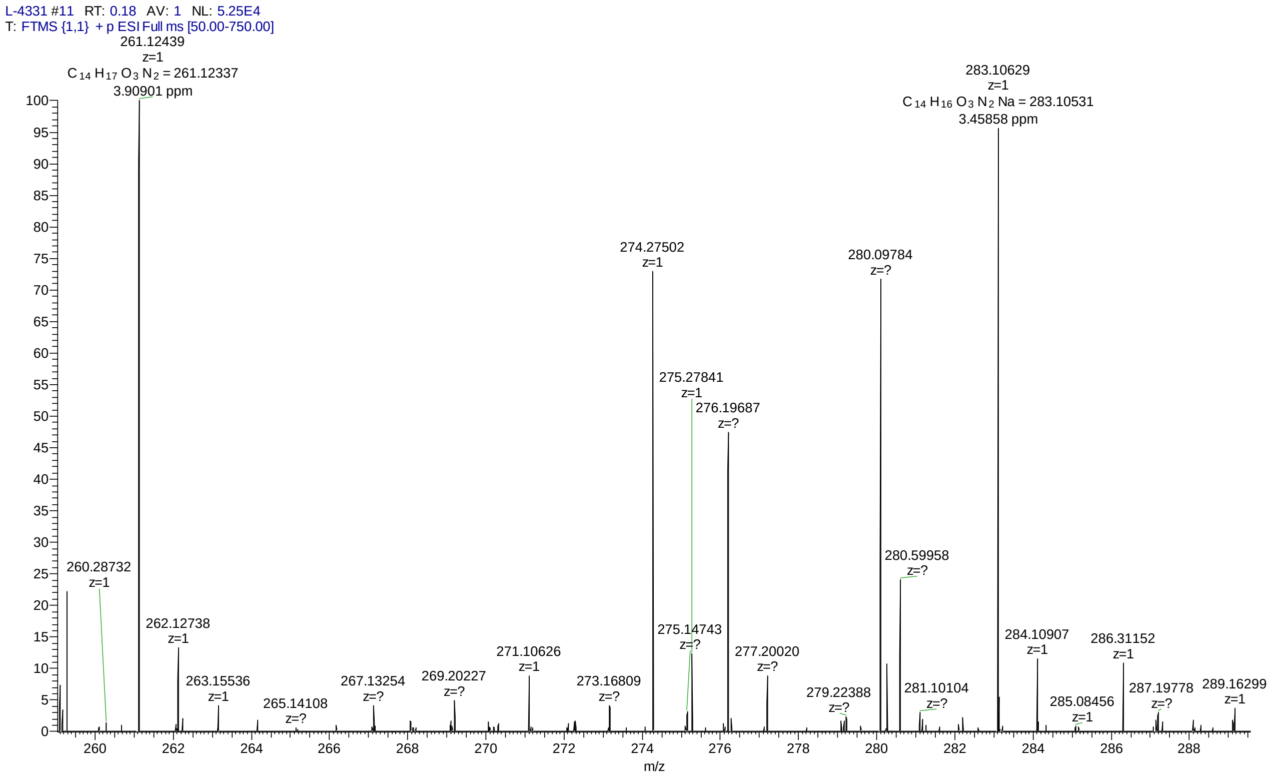

Figure S25. HR-ESI$^+$-MS spectrum of compound **5**.

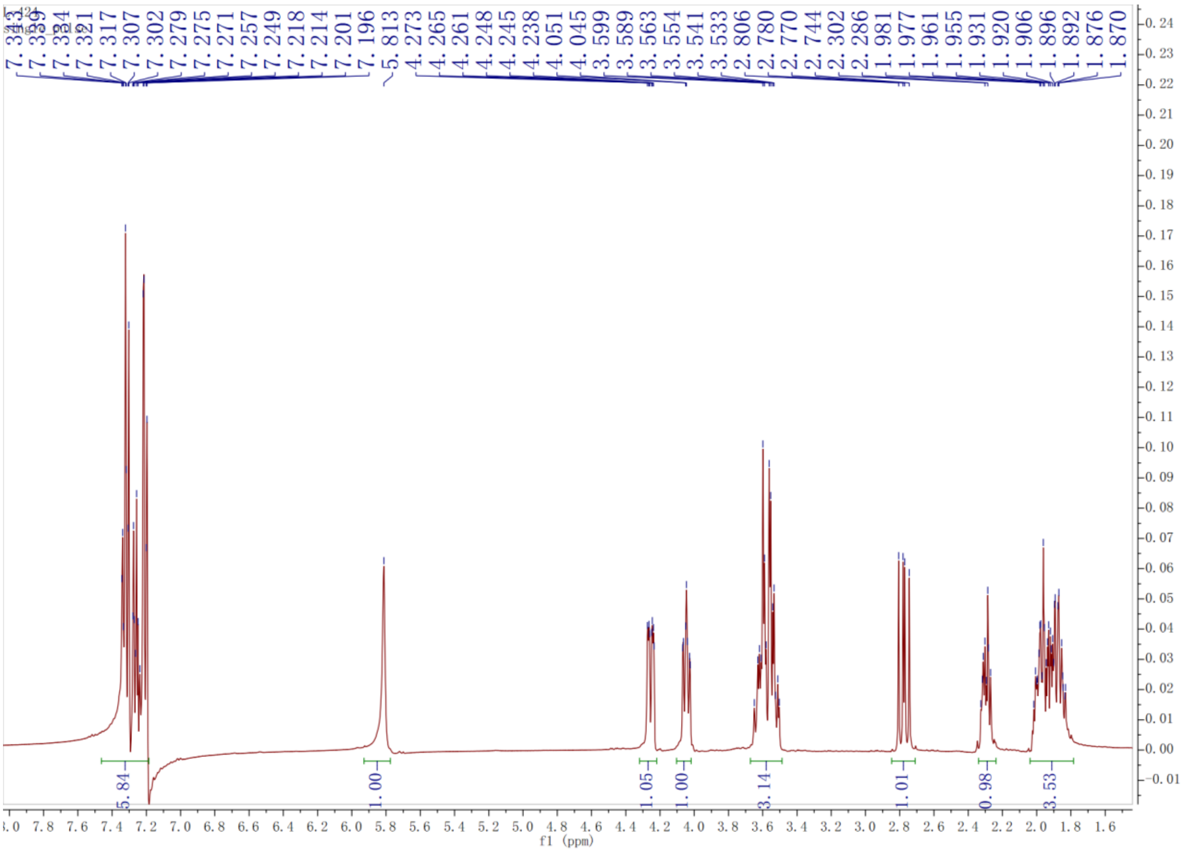

Figure S26. ^{1}H NMR spectrum of compound **6** ($CDCl_3$).

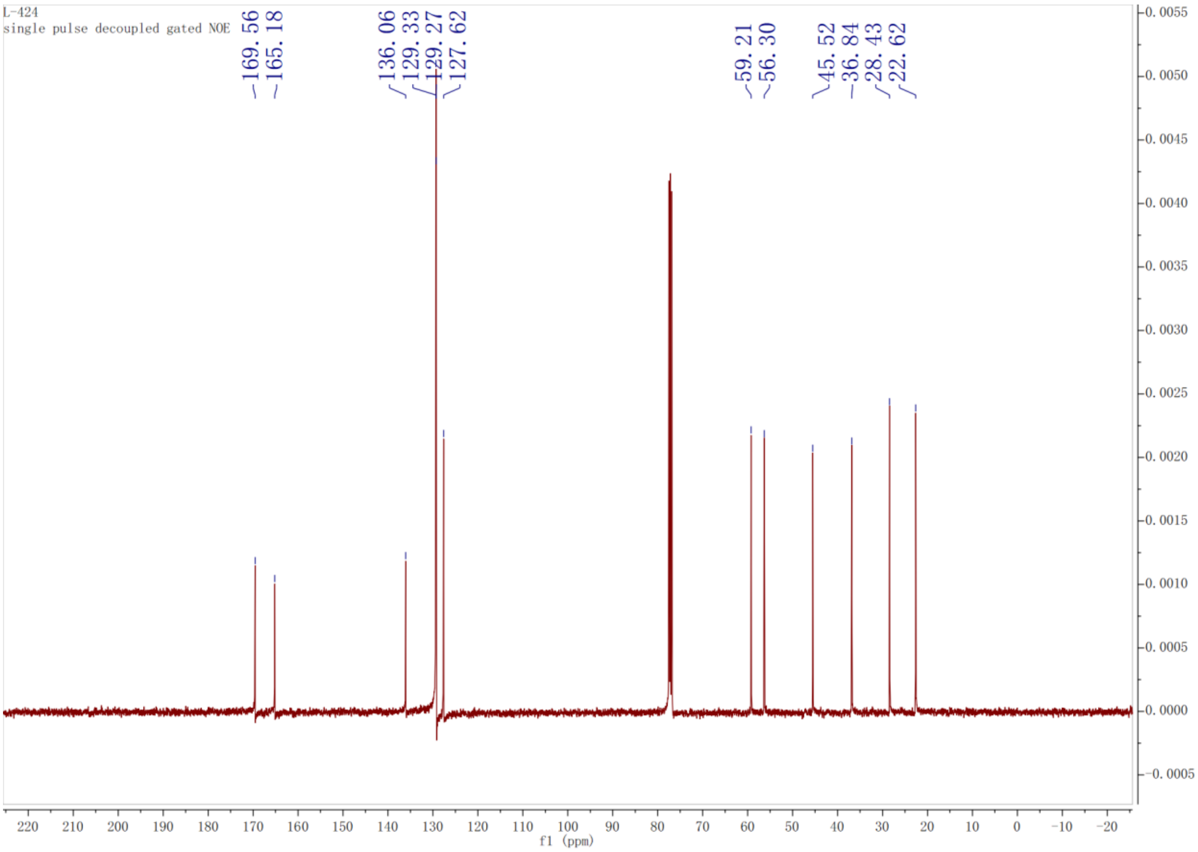

Figure S27. ^{13}C NMR spectrum of compound **6** ($CDCl_3$).

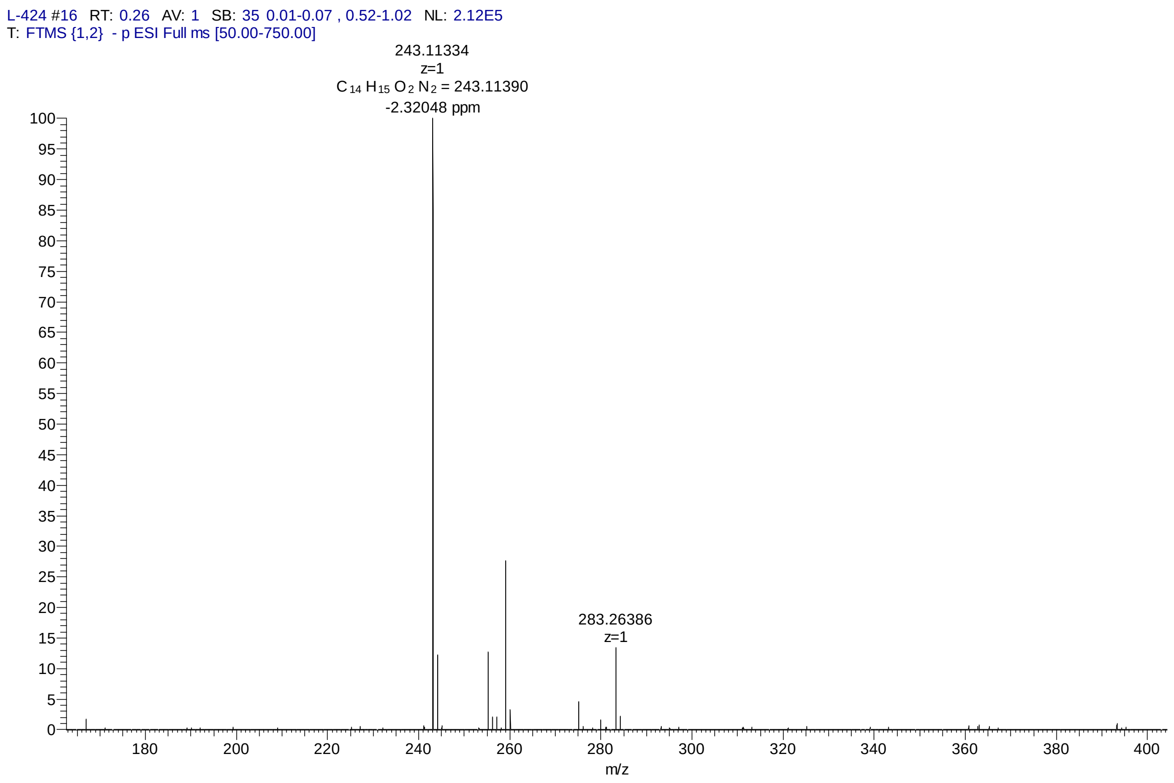

Figure S28. HR-ESI$^-$-MS spectrum of compound **6**.

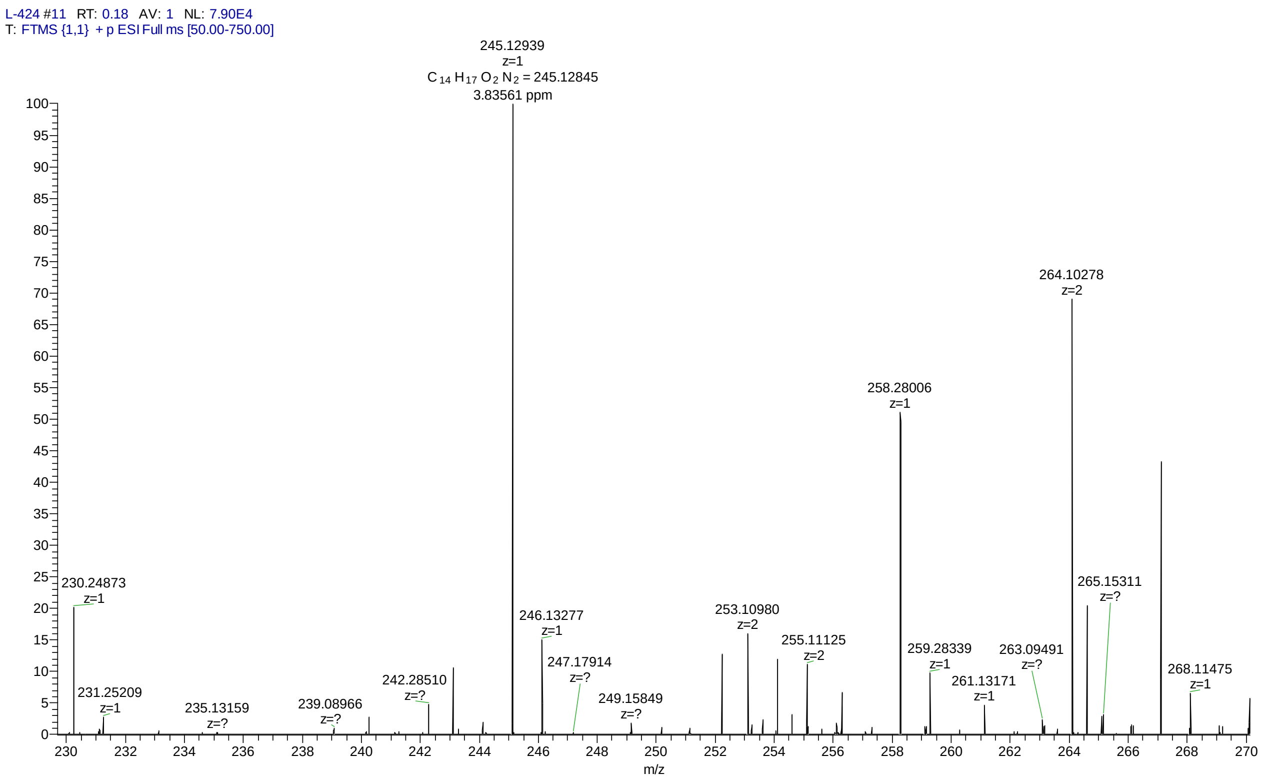

Figure S29. HR-ESI$^+$-MS spectrum of compound **6**.

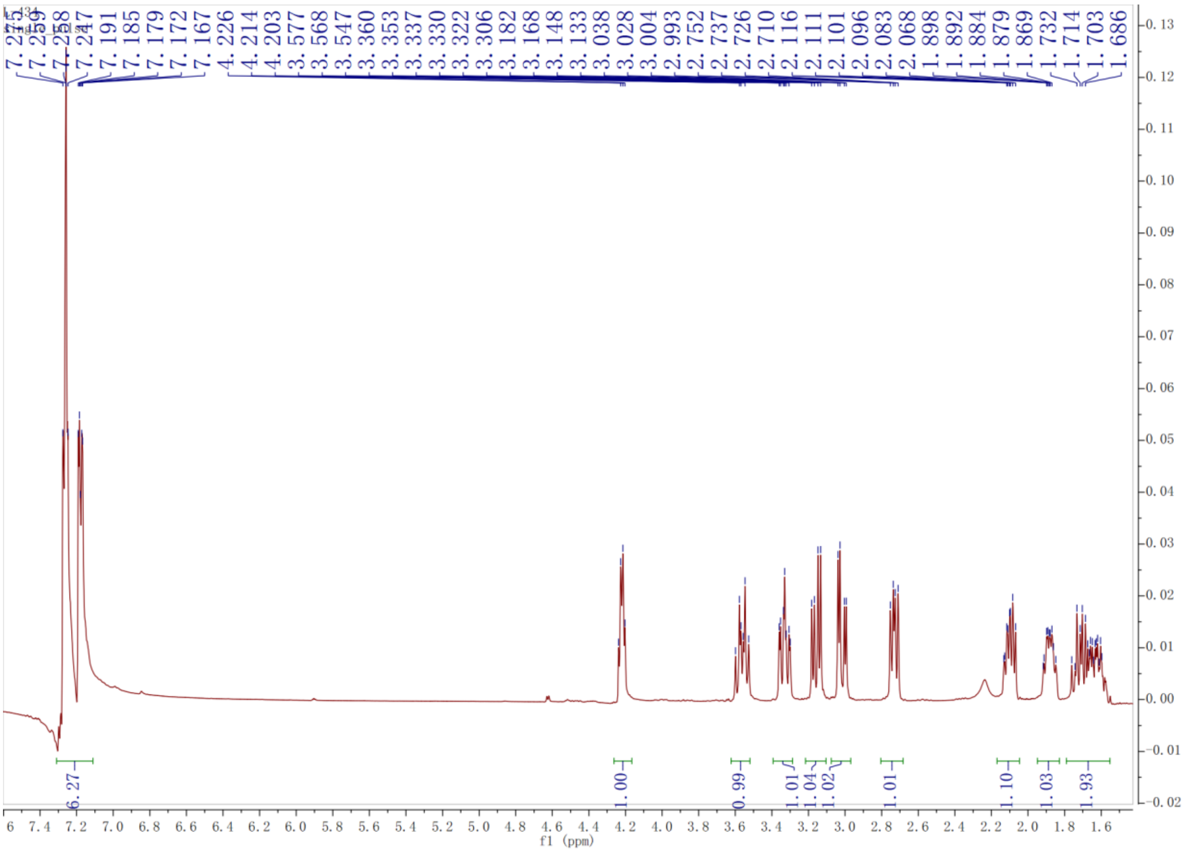

Figure S30. ^{1}H NMR spectrum of compound **7** ($CDCl_3$).

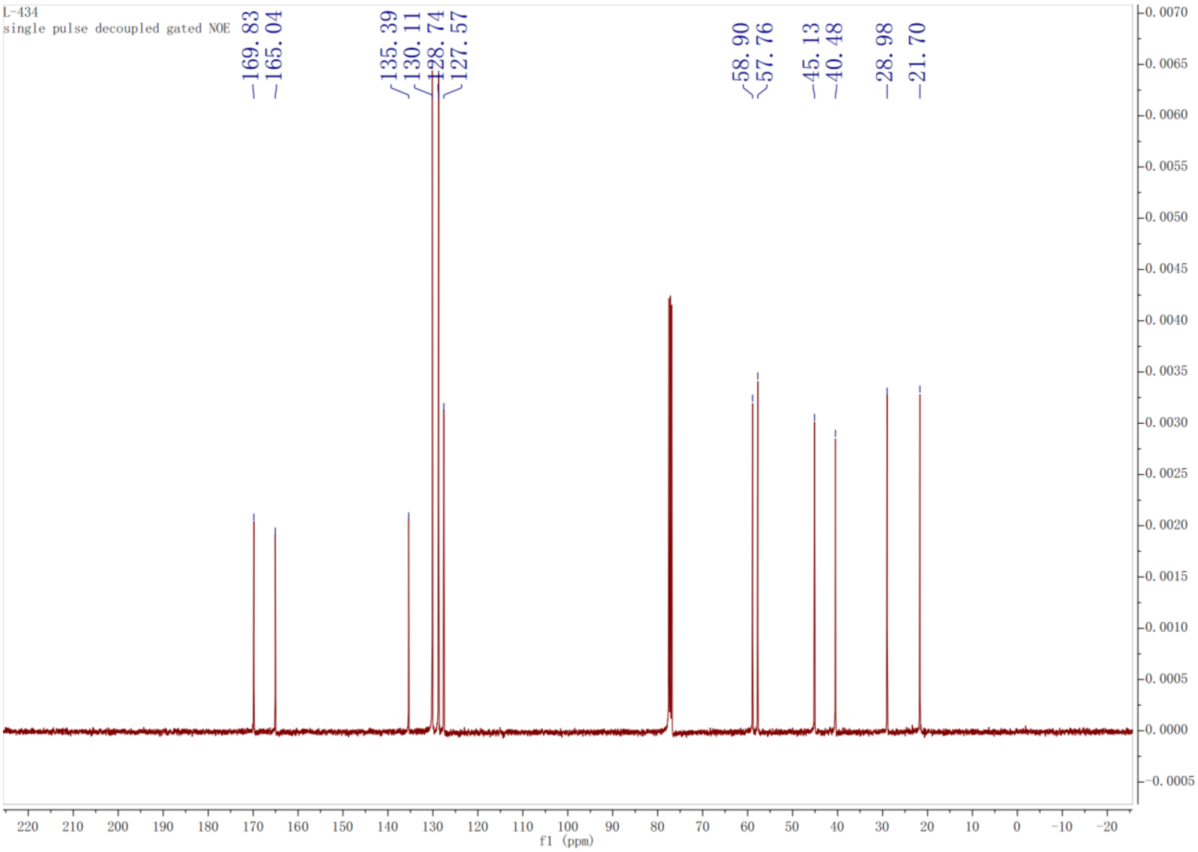

Figure S31. ^{13}C NMR spectrum of compound **7** ($CDCl_3$).

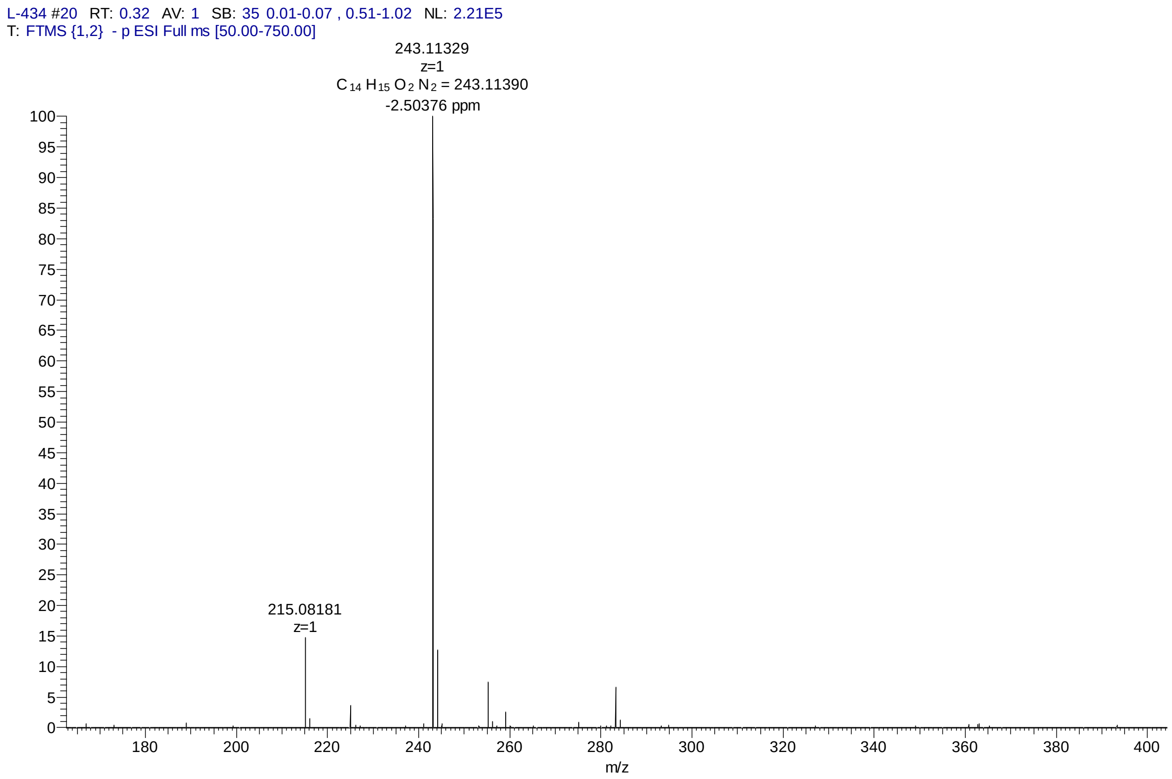

Figure S32. HR-ESI$^-$-MS spectrum of compound **7**.

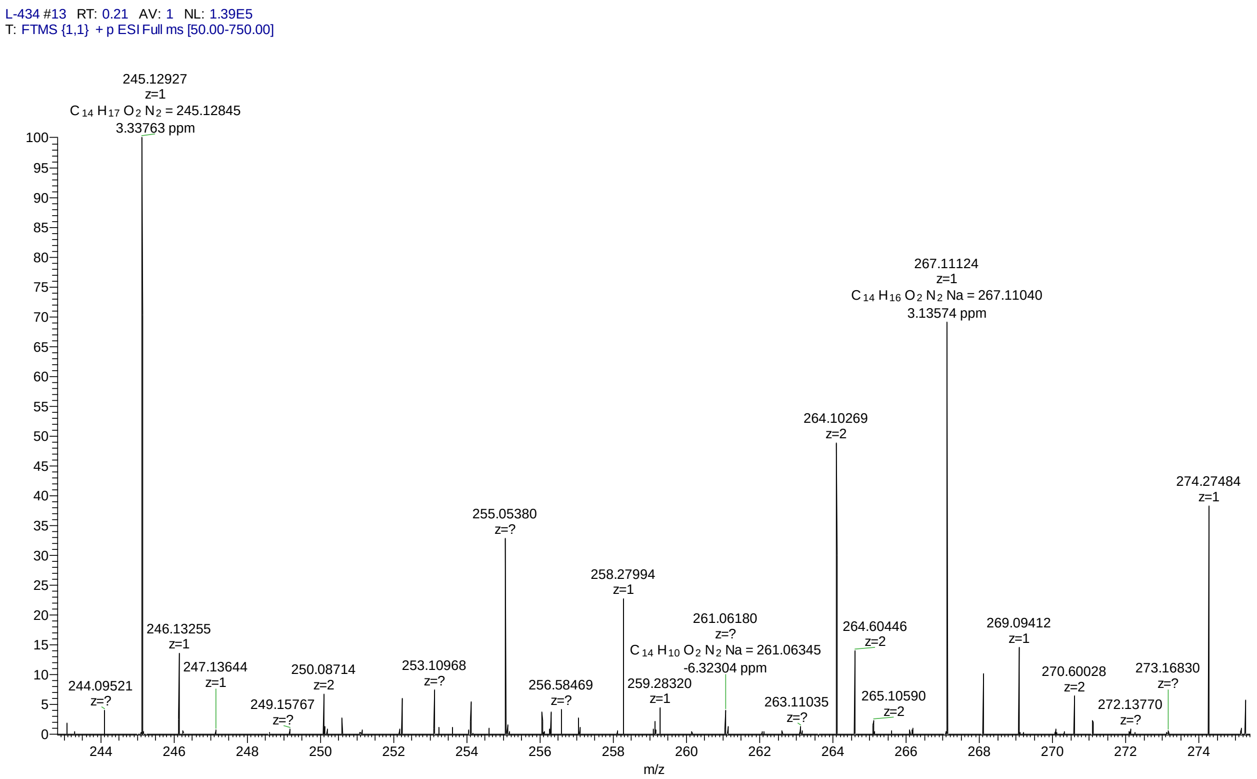

Figure S33. HR-ESI$^+$-MS spectrum of compound **7**.

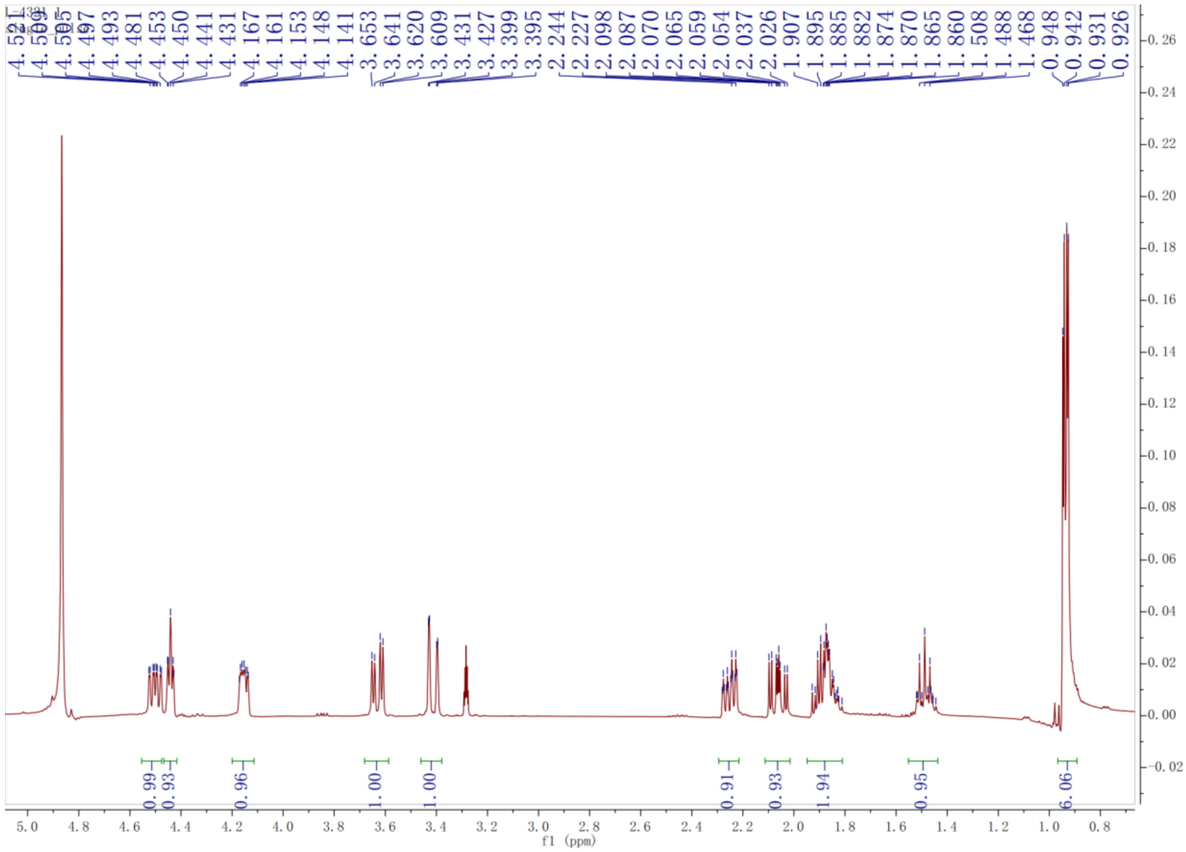

Figure S34. ^{1}H NMR spectrum of compound **8** (CD_3OD).

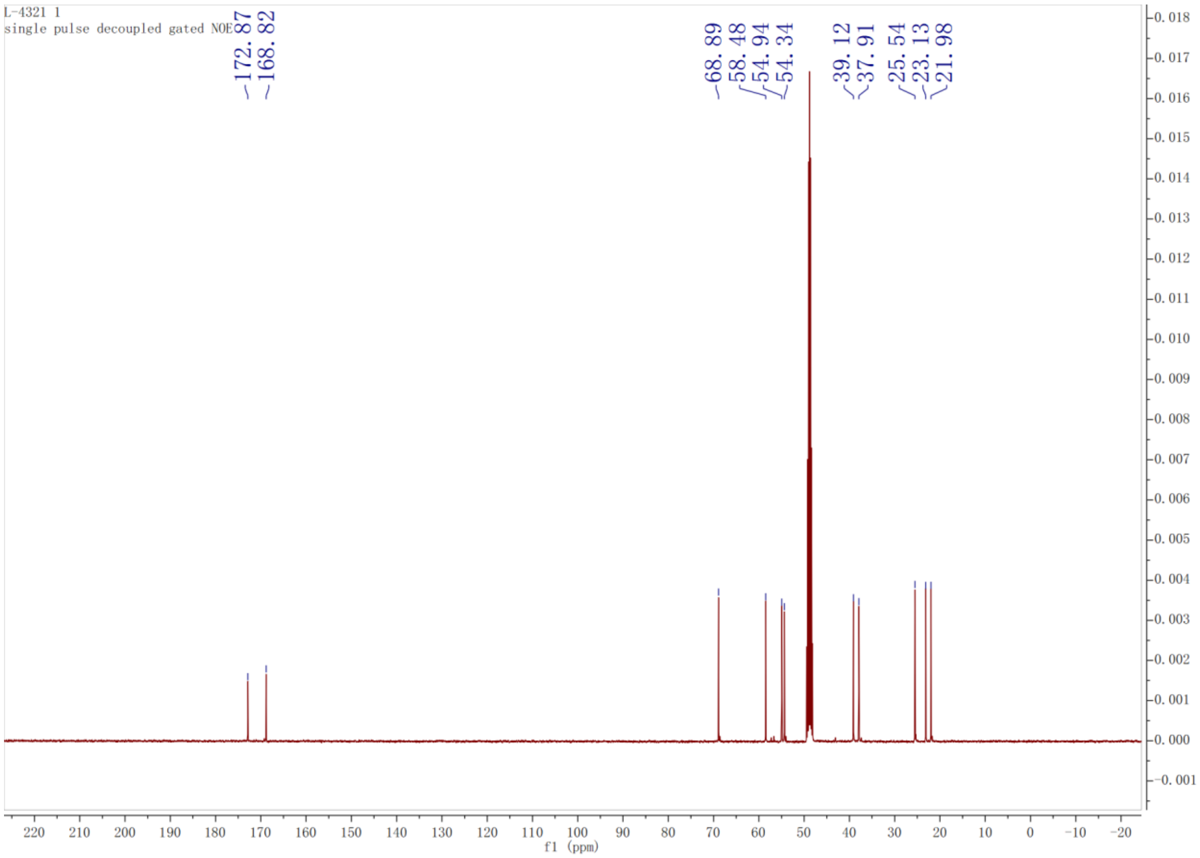

Figure S35. ^{13}C NMR spectrum of compound **8** (CD_3OD).

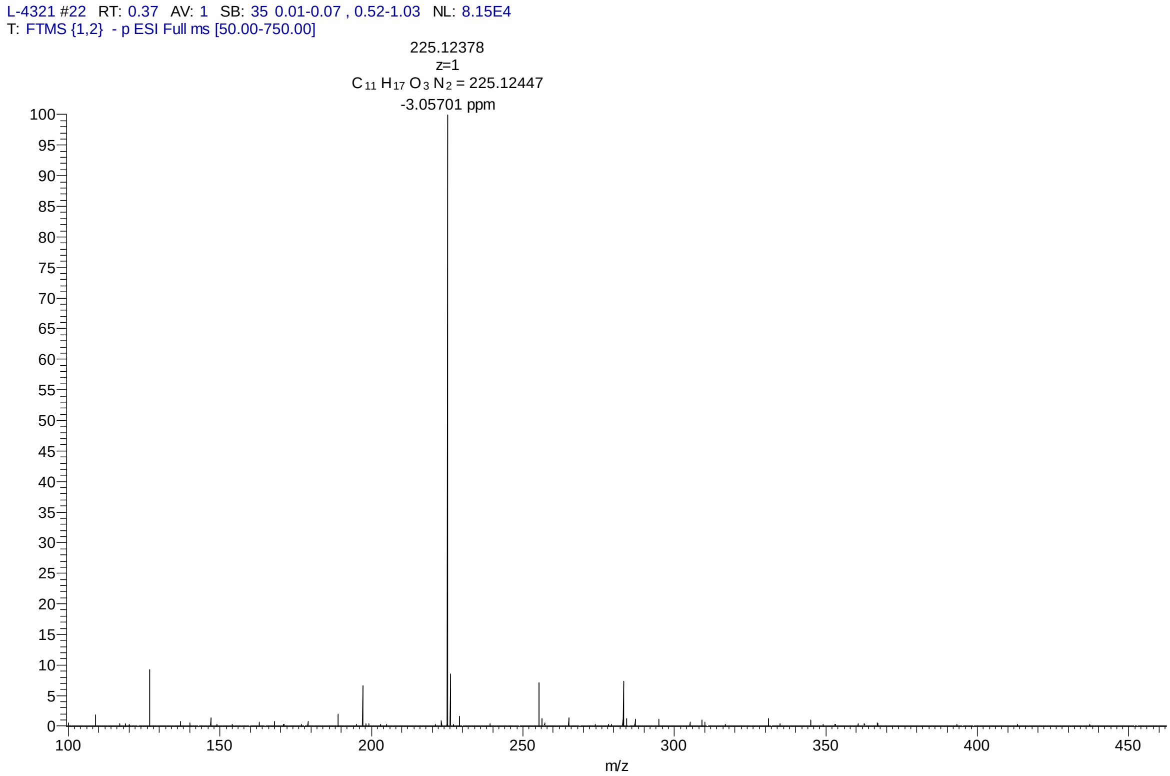

Figure S36. HR-ESI$^-$-MS spectrum of compound **8**.

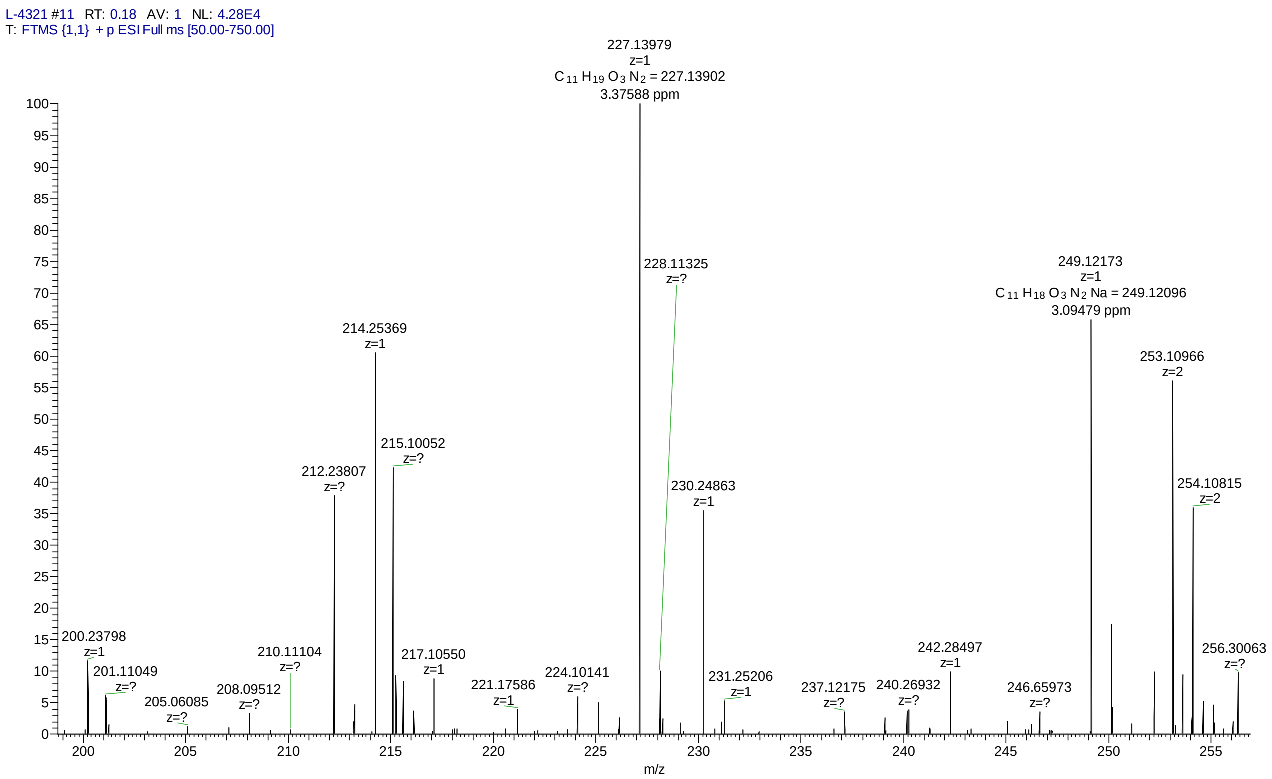

Figure S37. HR-ESI$^+$-MS spectrum of compound **8**.

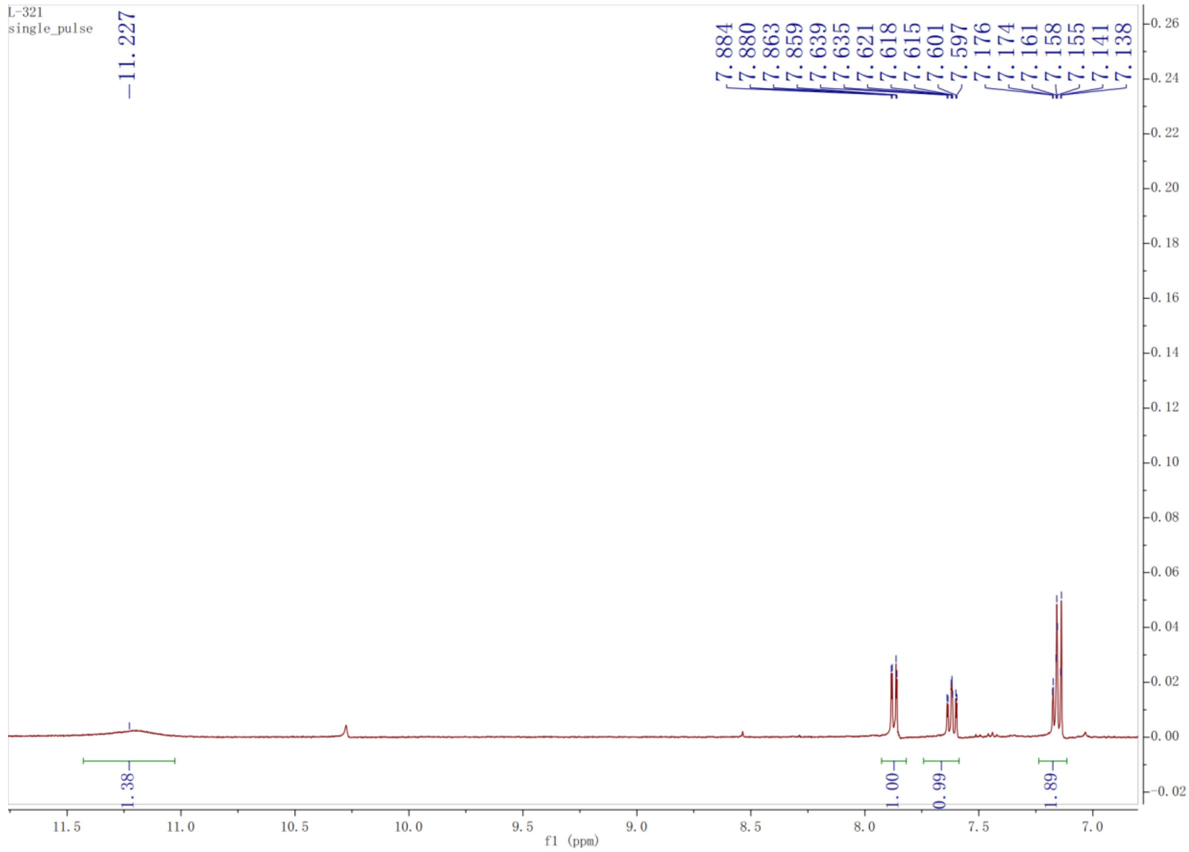

Figure S38. ^{1}H NMR spectrum of compound **9** (DMSO-d_6).

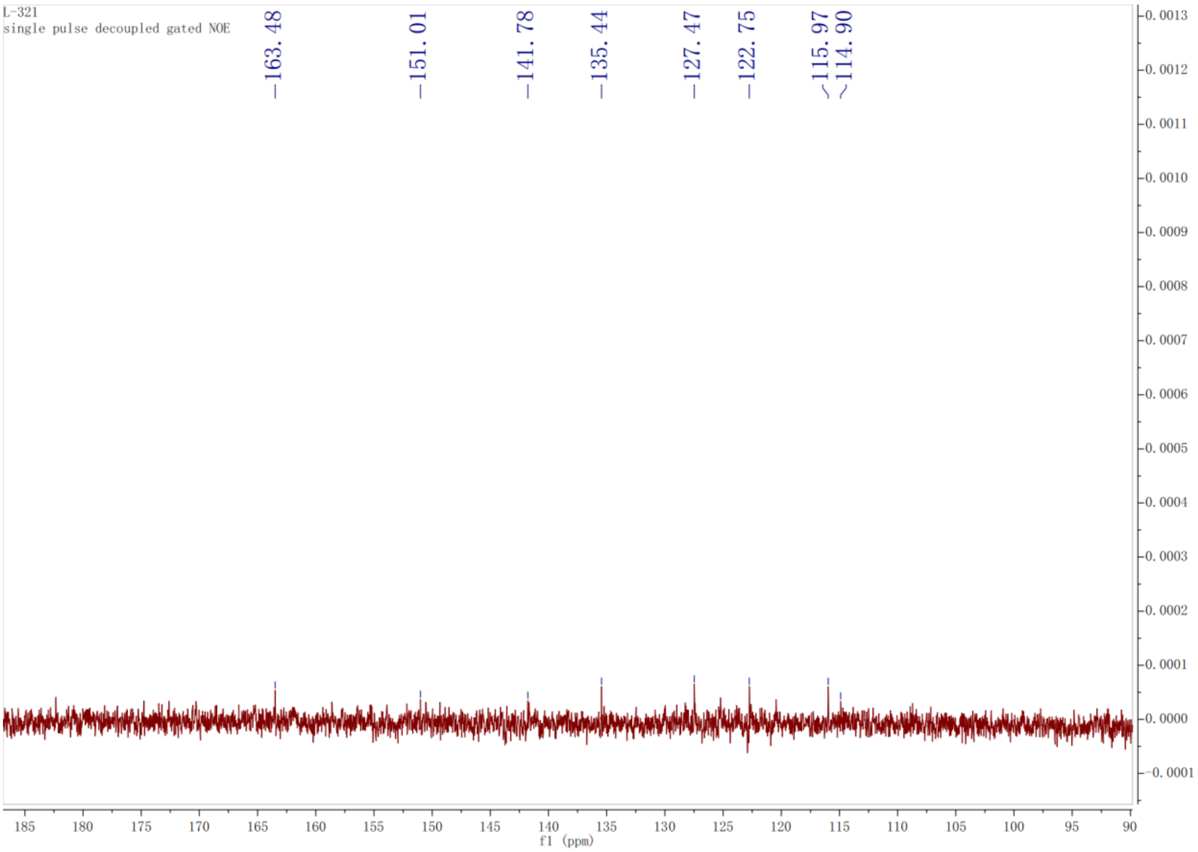

Figure S39. ^{13}C NMR spectrum of compound **9** (DMSO-d_6).

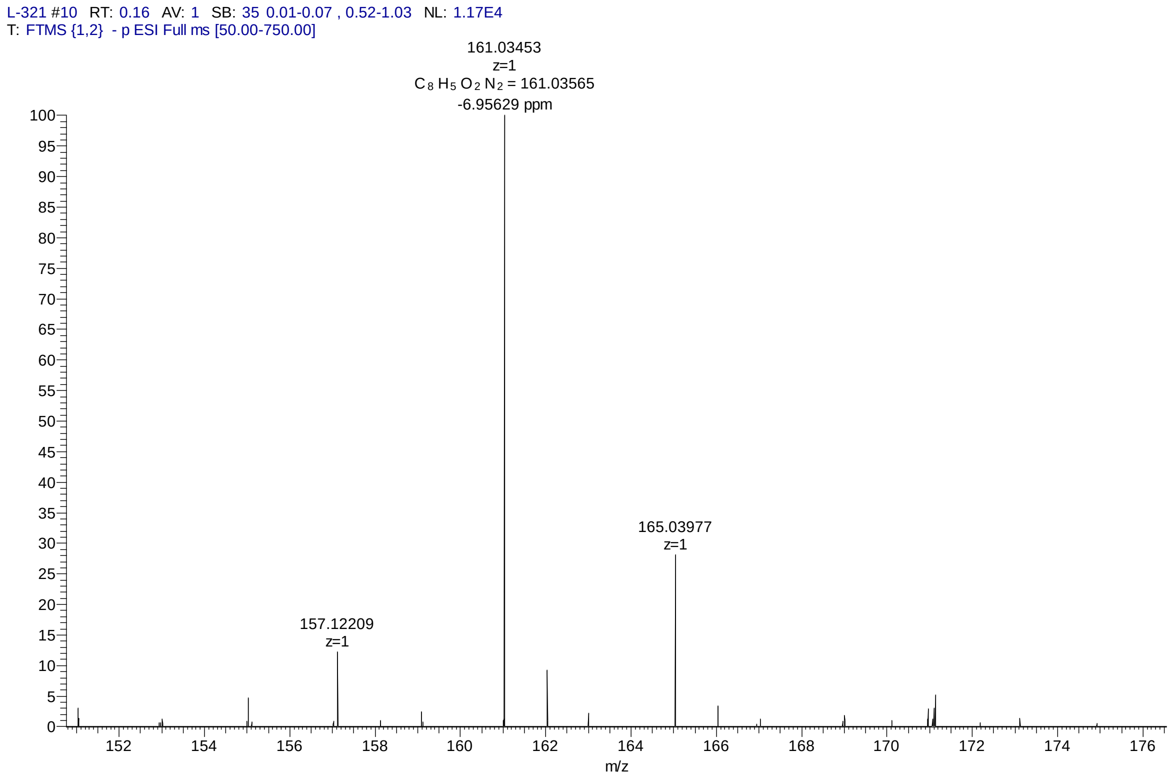

Figure S40. HR-ESI$^-$-MS spectrum of compound **9**.

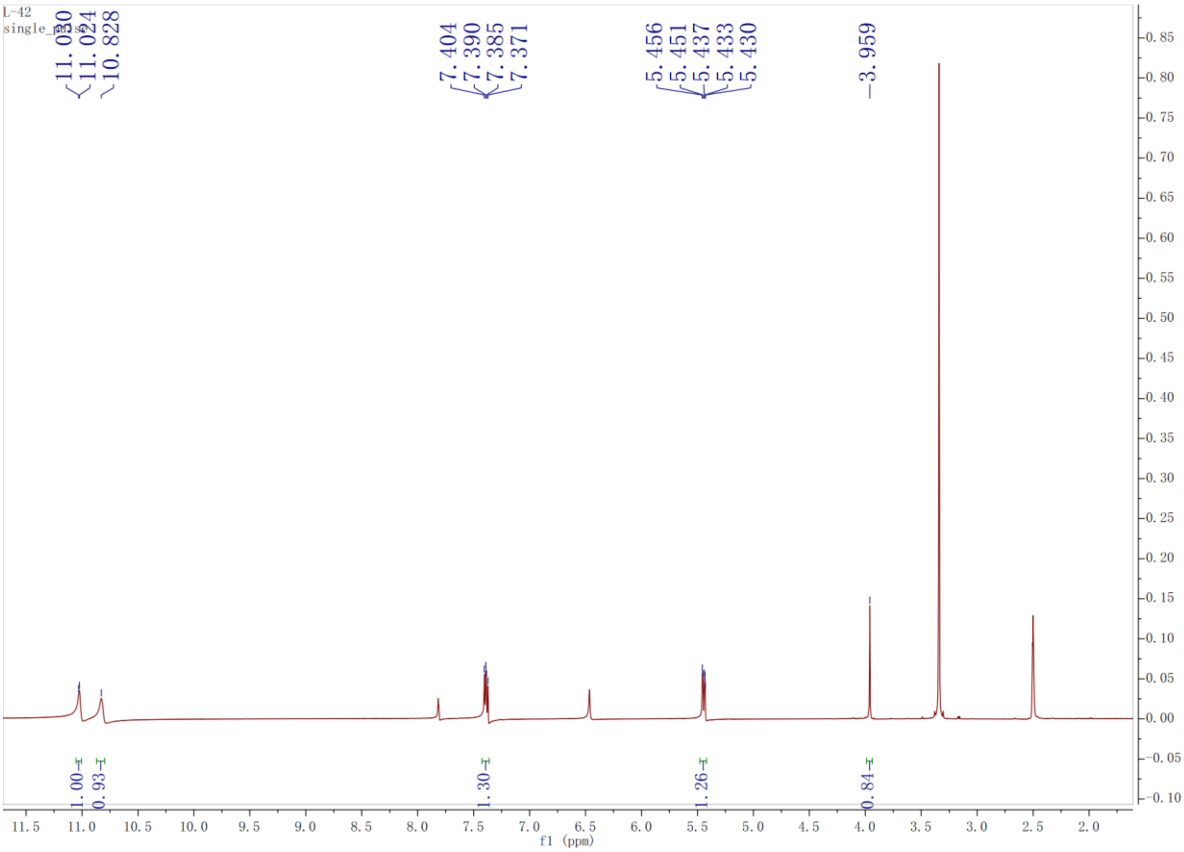

Figure S41. ^{1}H NMR spectrum of compound **10** (DMSO-d_6).

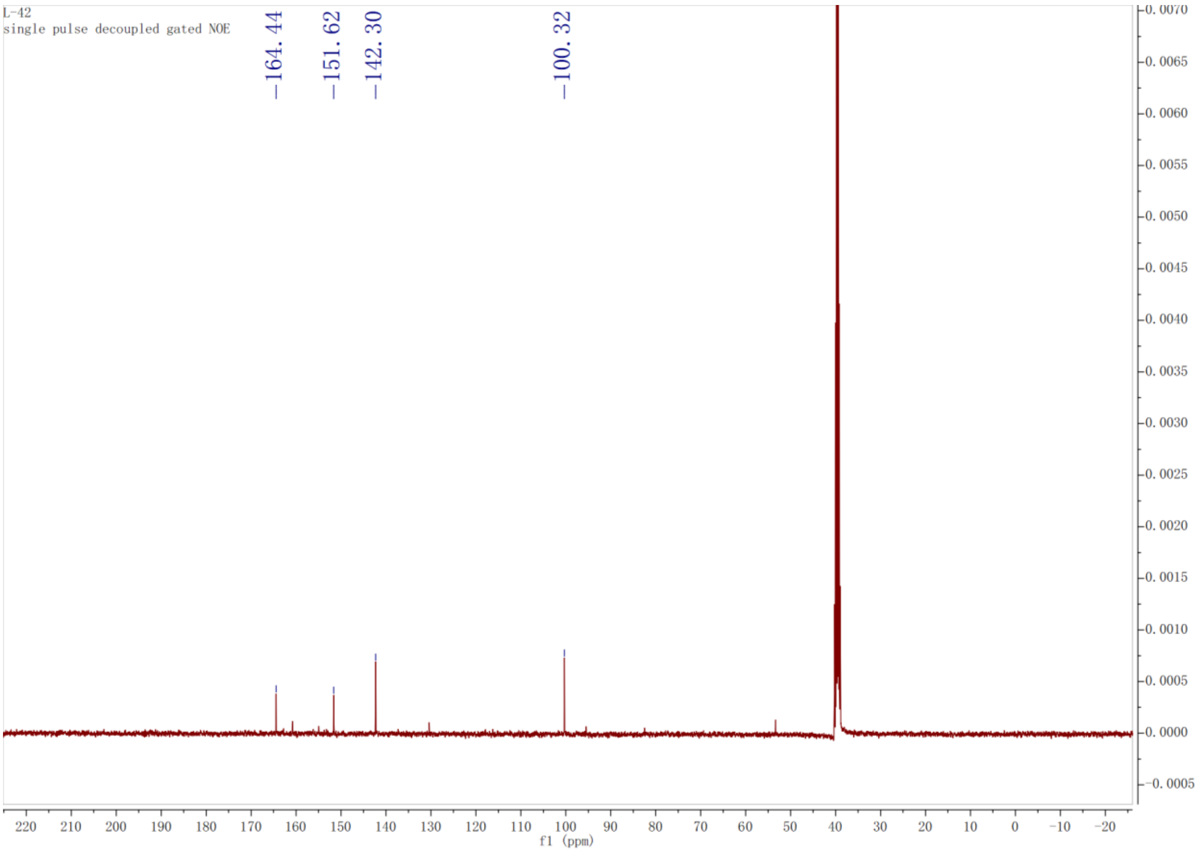

Figure S42. ^{13}C NMR spectrum of compound **10** (DMSO-d_6).

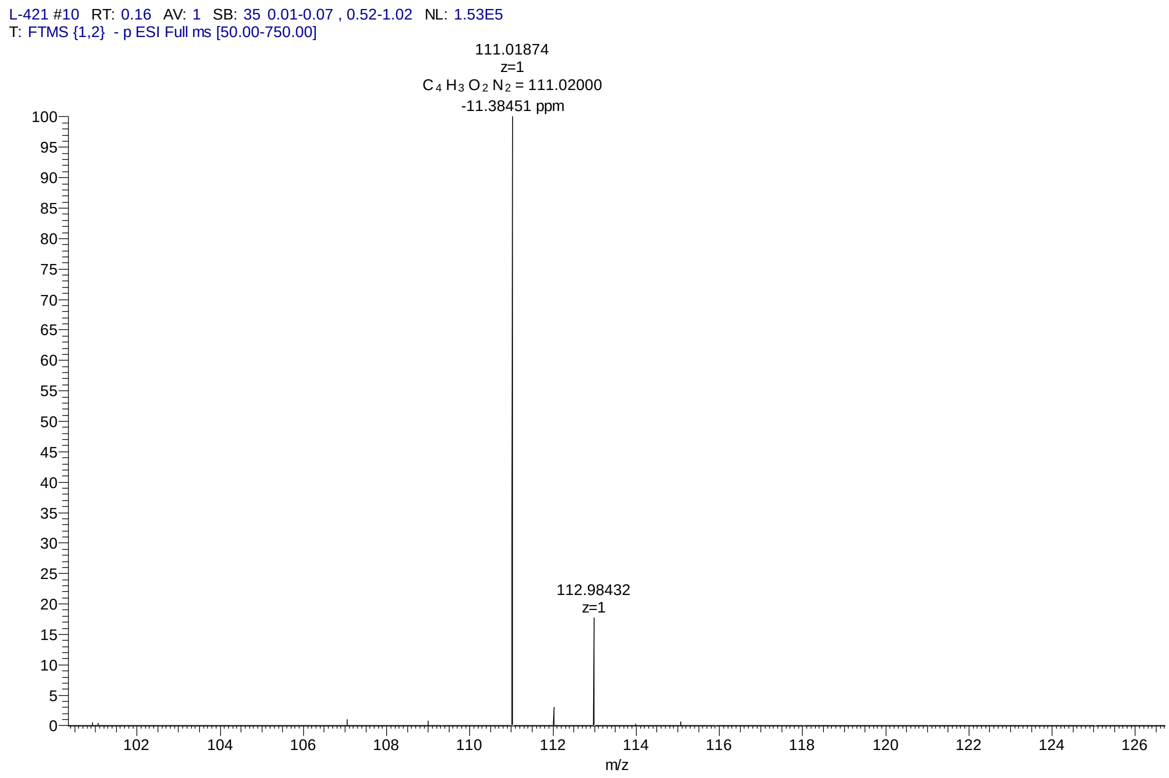

Figure S43. HR-ESI$^-$-MS spectrum of compound **10**.

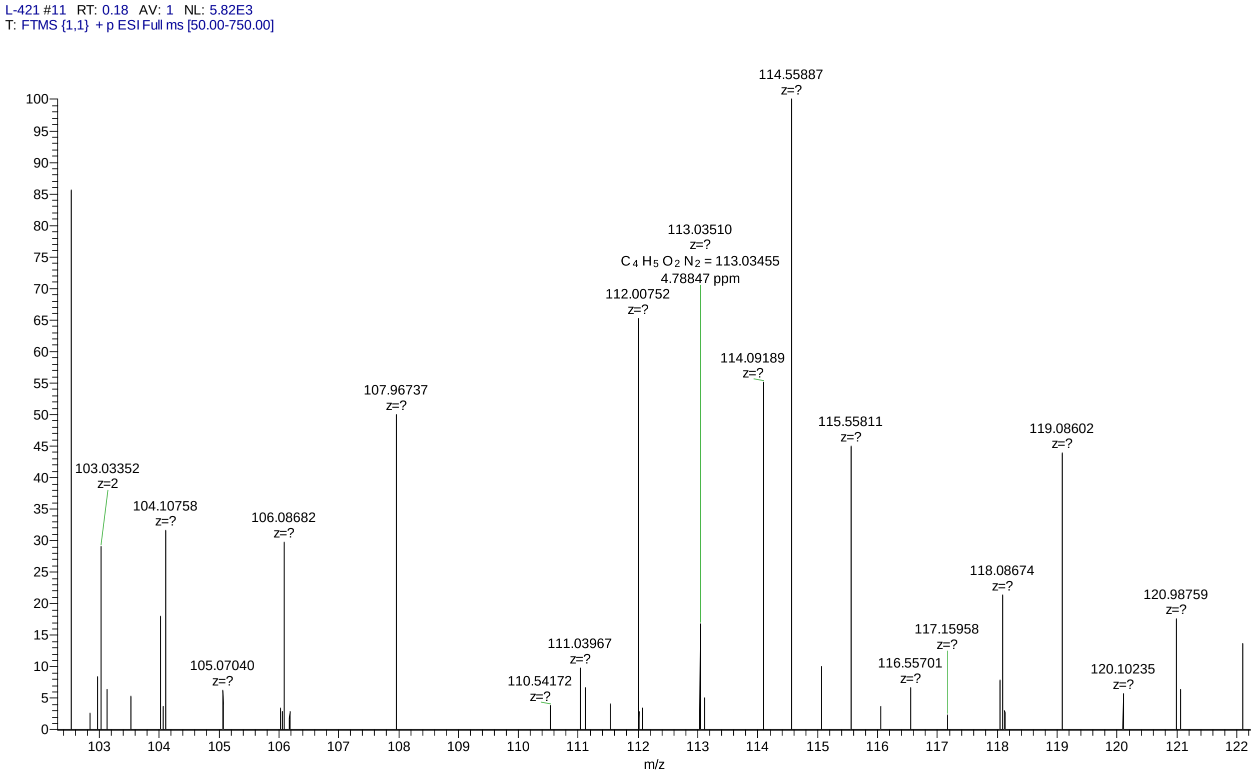

Figure S44. HR-ESI$^+$-MS spectrum of compound **10**.

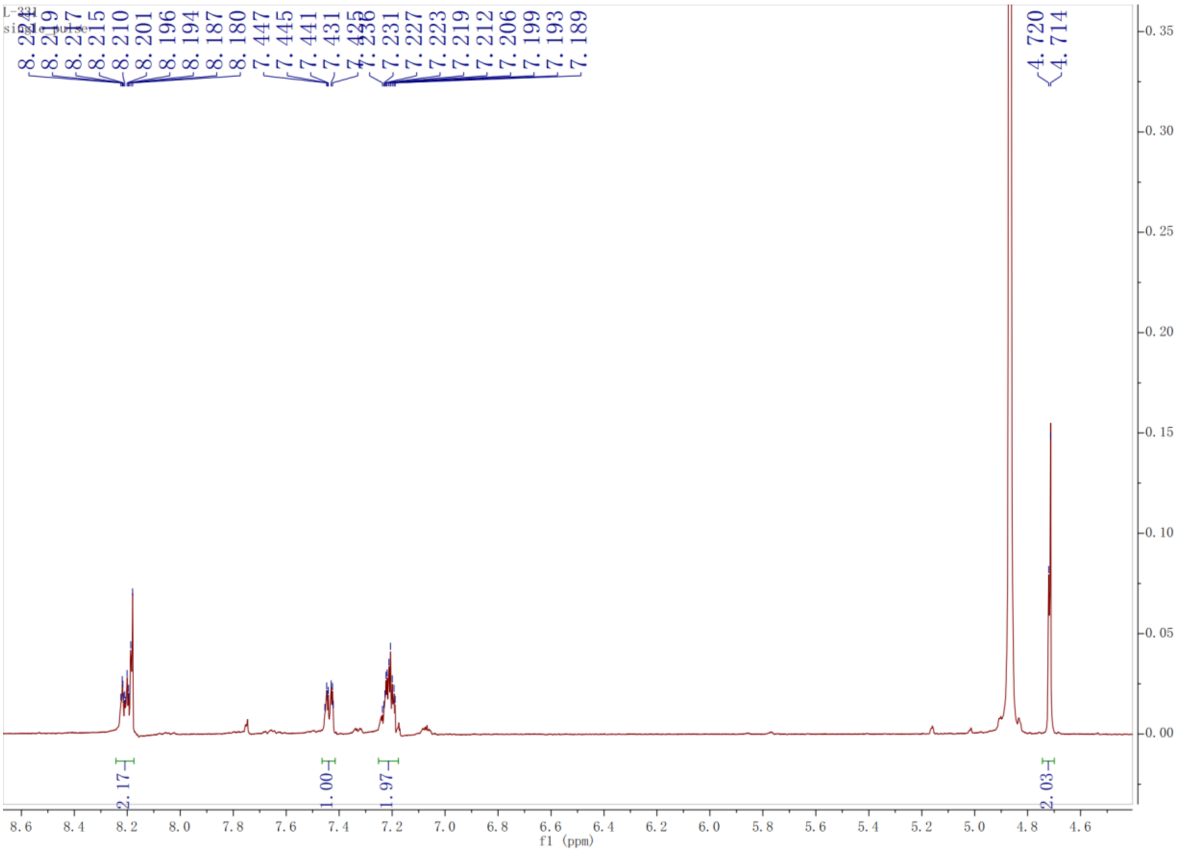

Figure S45. ^{1}H NMR spectrum of compound **11** (CD_3OD).

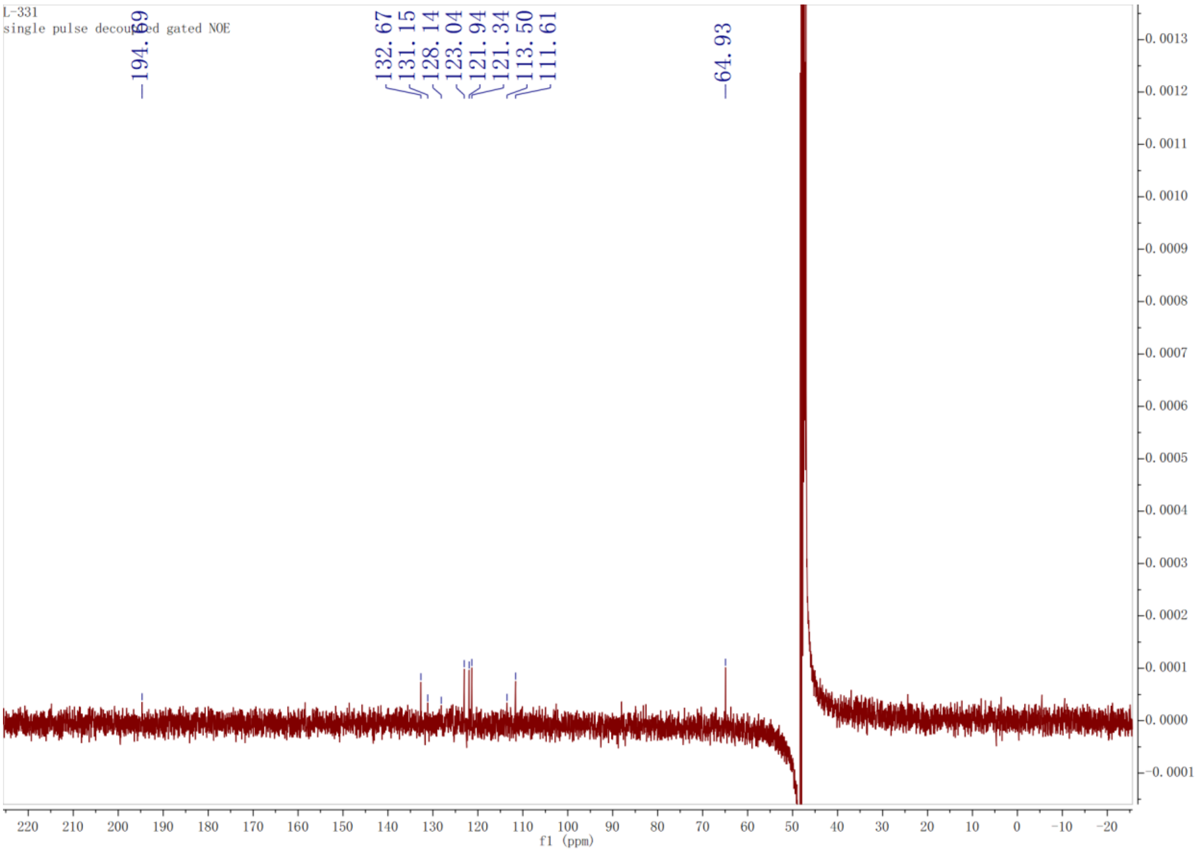

Figure S46. ^{13}C NMR spectrum of compound **11** (CD_3OD).

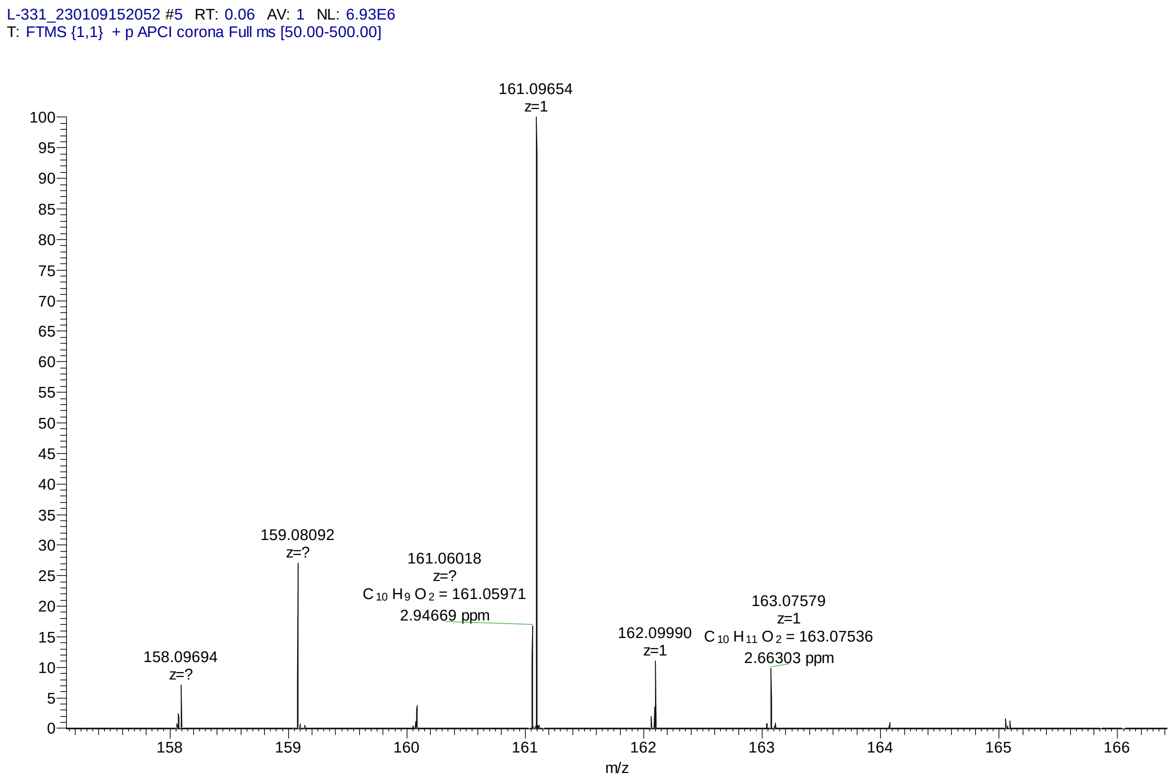

Figure S47. HR-APCI-MS spectrum of compound **11**.

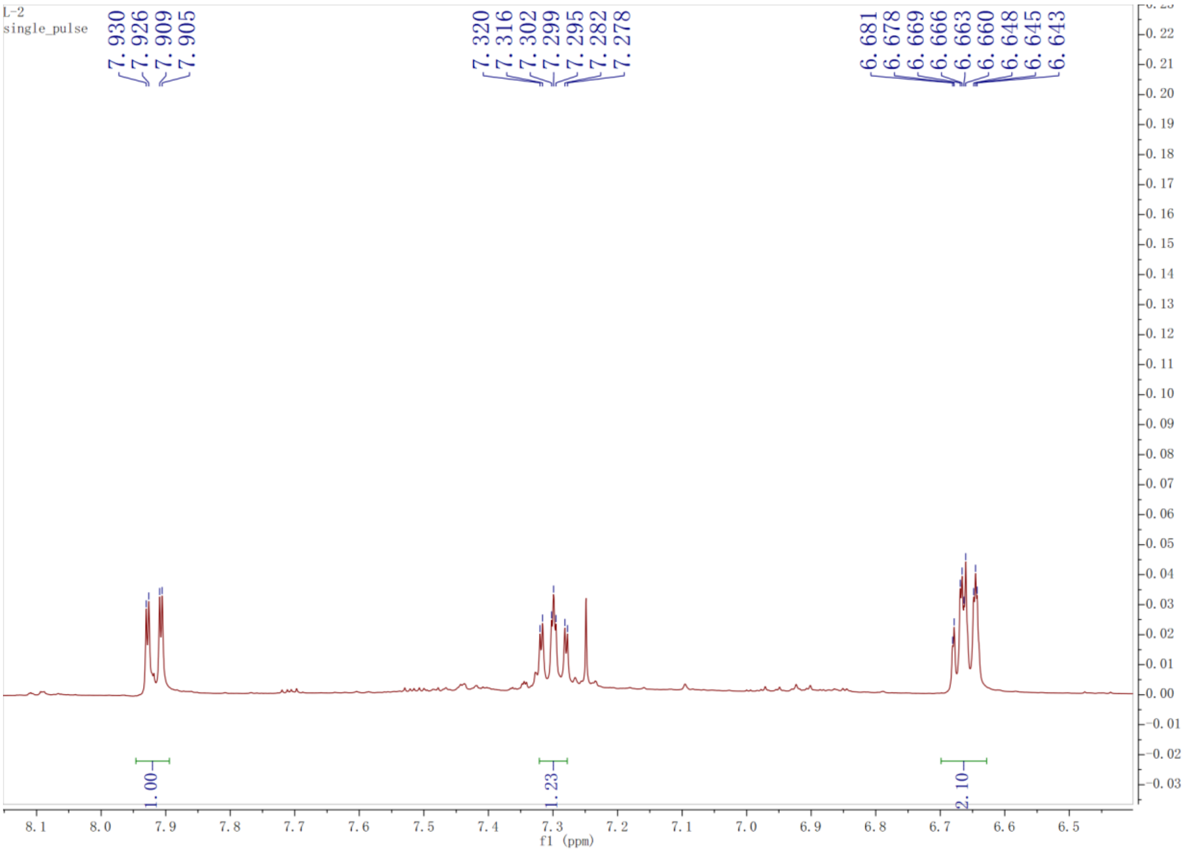

Figure S48. ^{1}H NMR spectrum of compound **12** ($CDCl_3$).

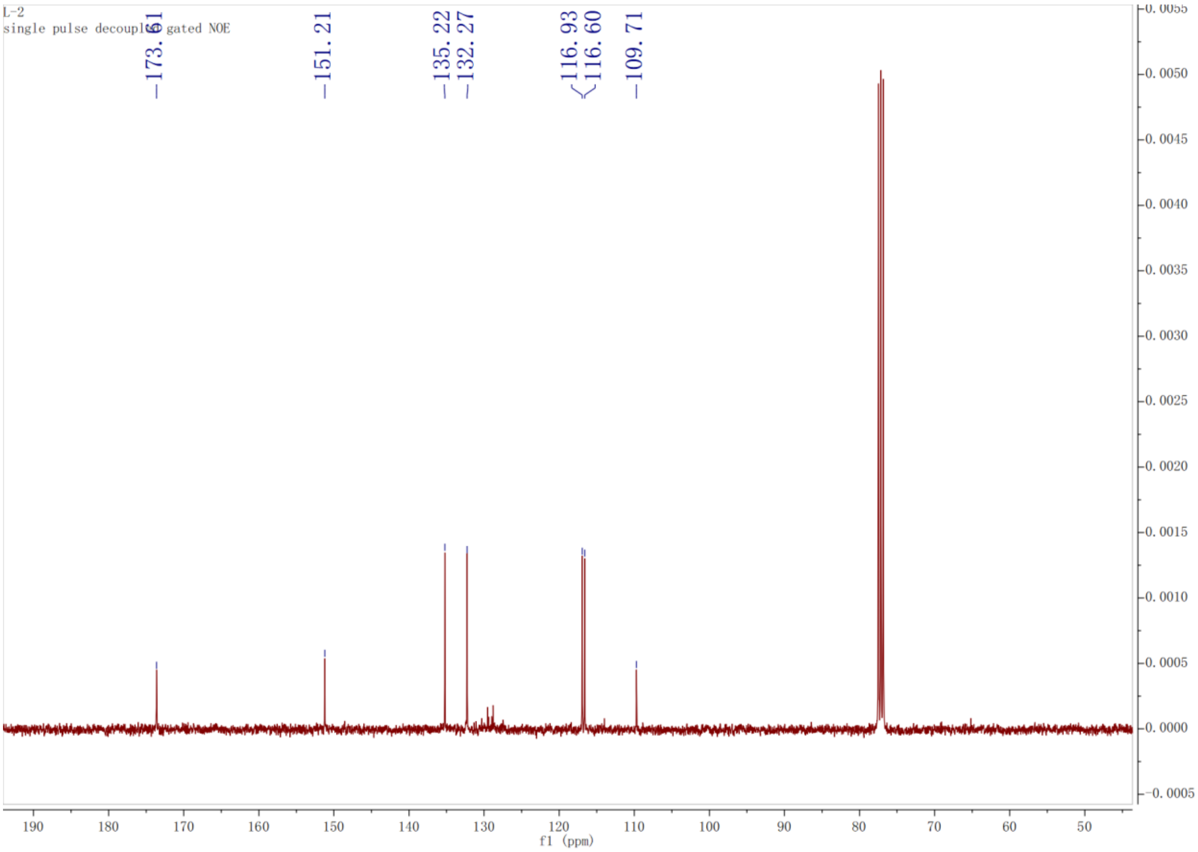

Figure S49. ^{13}C NMR spectrum of compound **12** ($CDCl_3$).

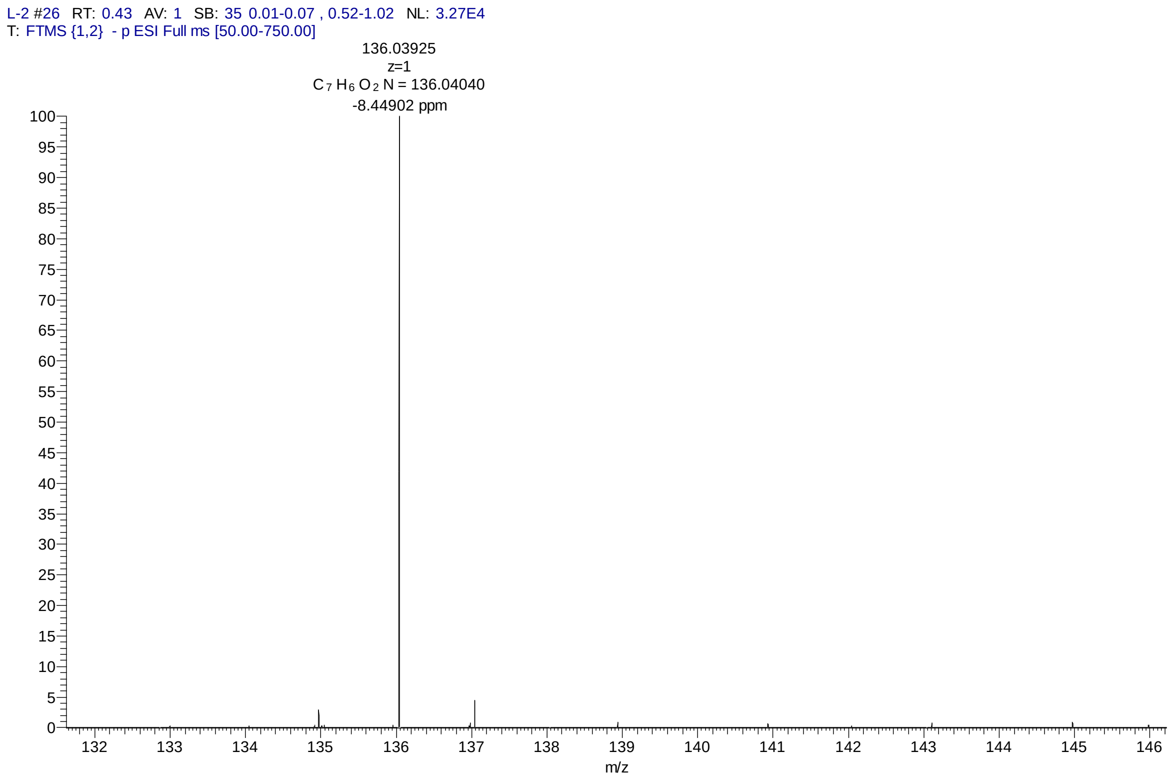

Figure S50. HR-ESI⁻-MS spectrum of compound **12**.

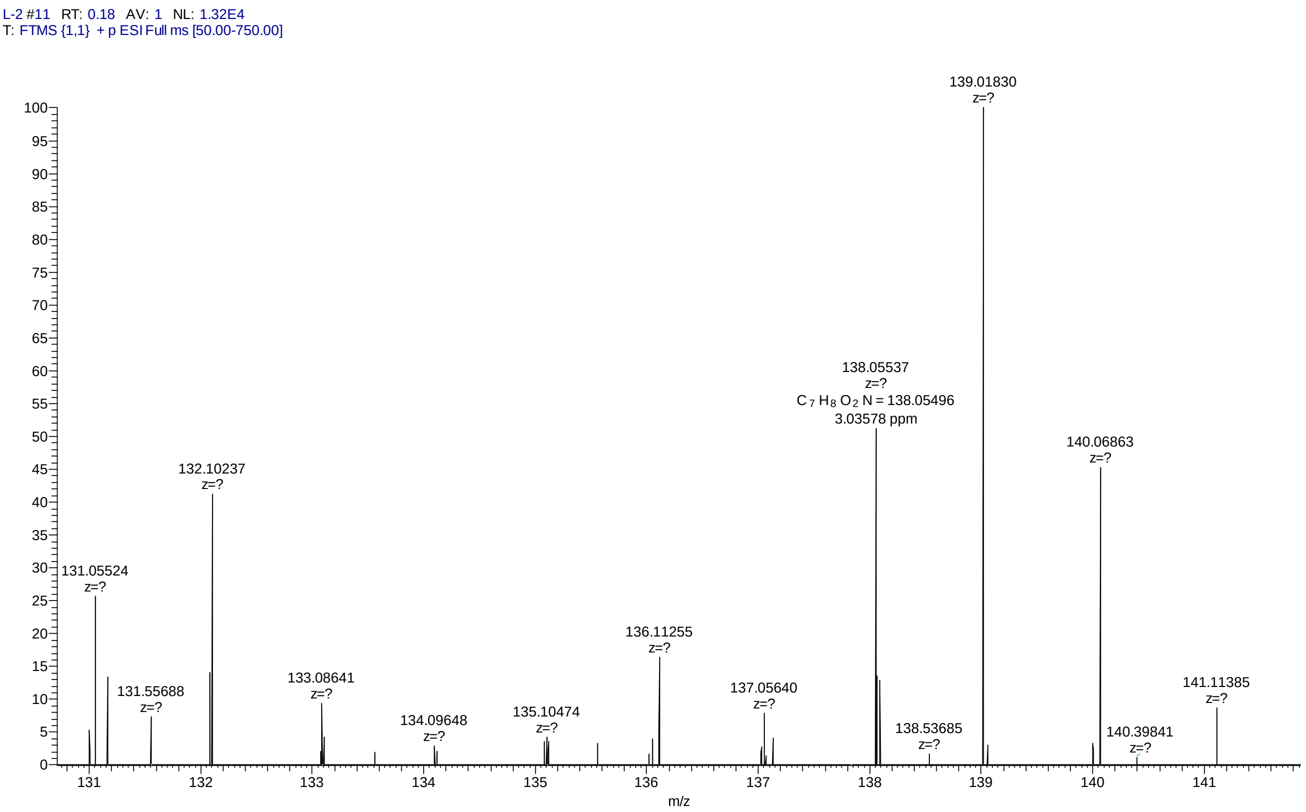

Figure S51. HR-ESI$^+$-MS spectrum of compound **12**.

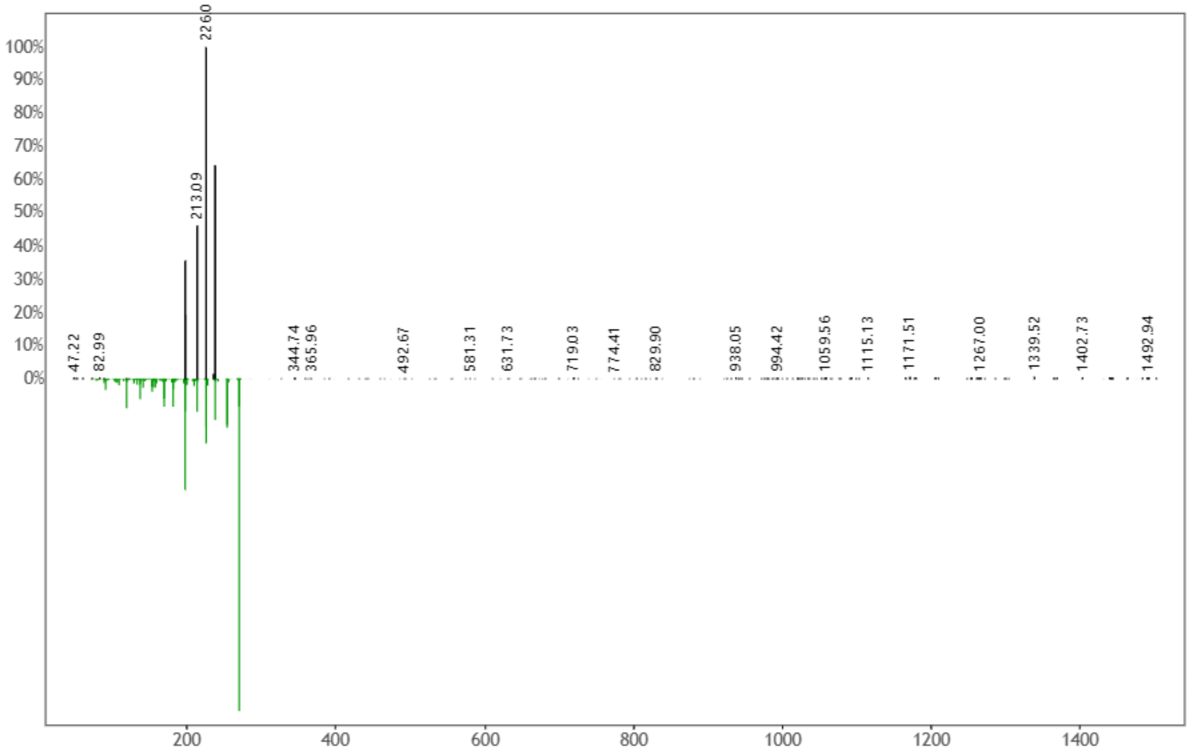

Figure S52. MS/MS spectrum of compound **a** (black) compared with GNPS library spectrum (green).

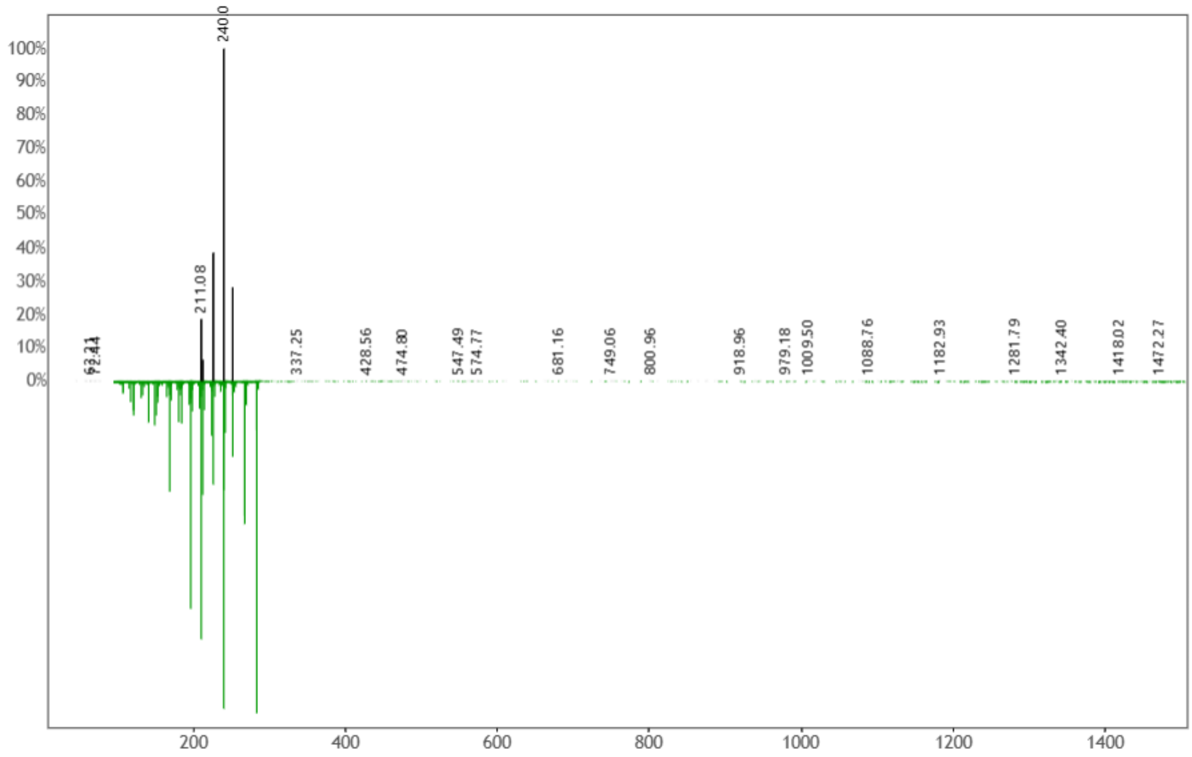

Figure S53. MS/MS spectrum of compound **b** (black) compared with GNPS library spectrum (green).

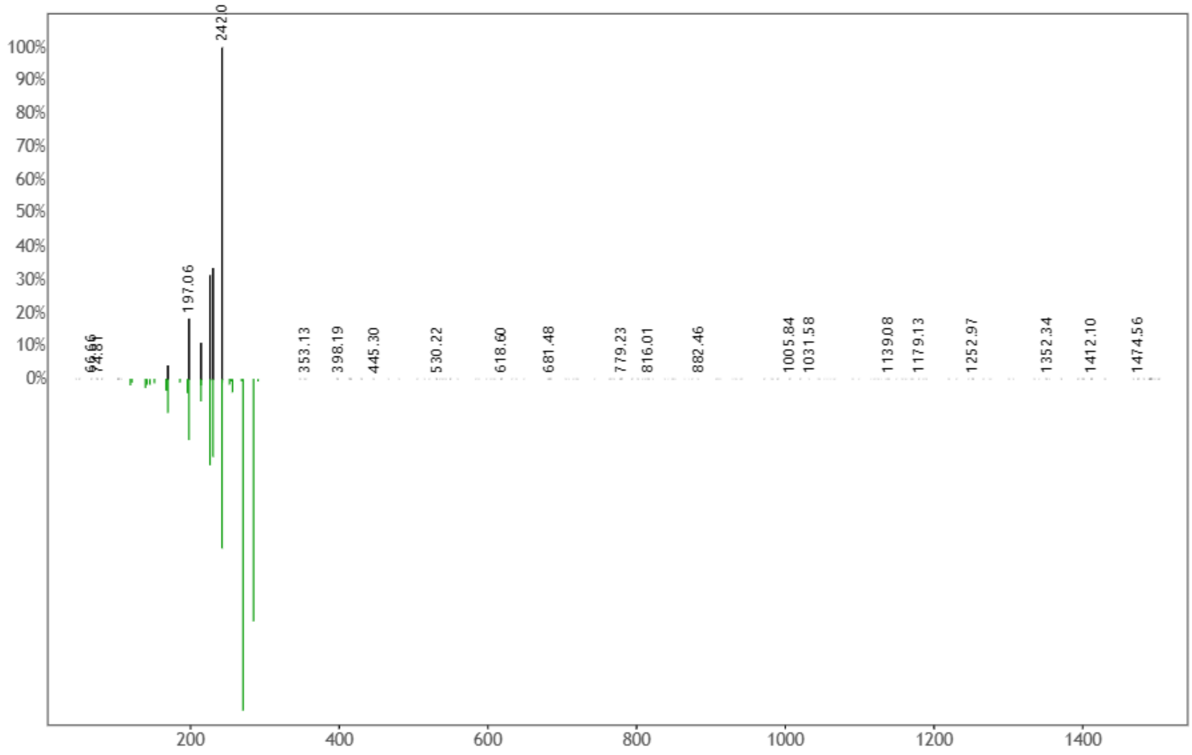

Figure S54. MS/MS spectrum of compound **c** (black) compared with GNPS library spectrum (green).

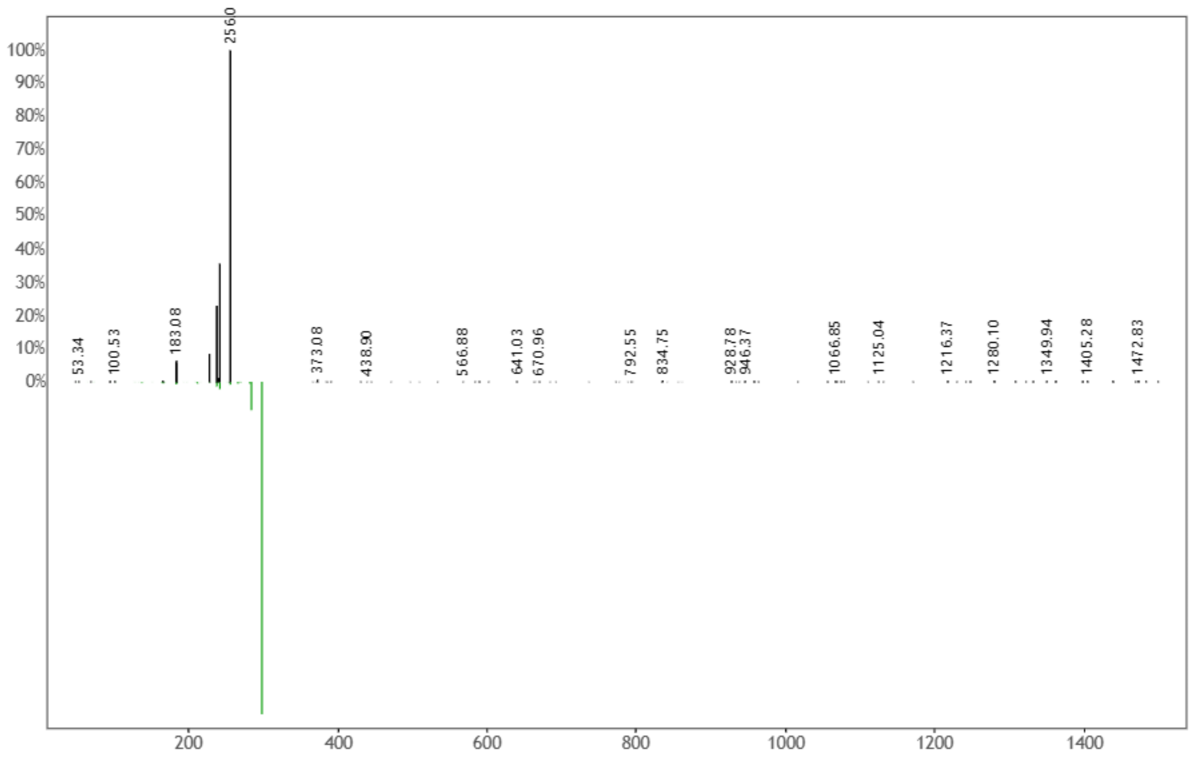

Figure S55. MS/MS spectrum of compound **d** (black) compared with GNPS library spectrum (green).

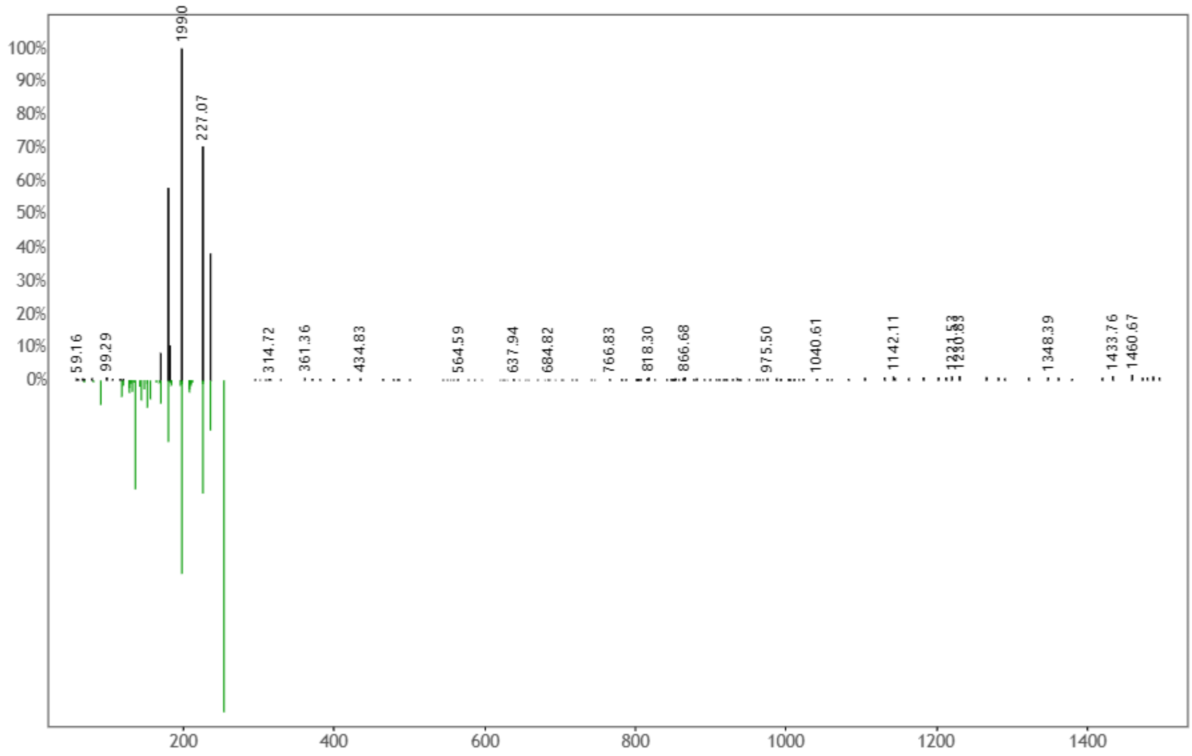

Figure S56. MS/MS spectrum of compound **e** (black) compared with GNPS library spectrum (green).

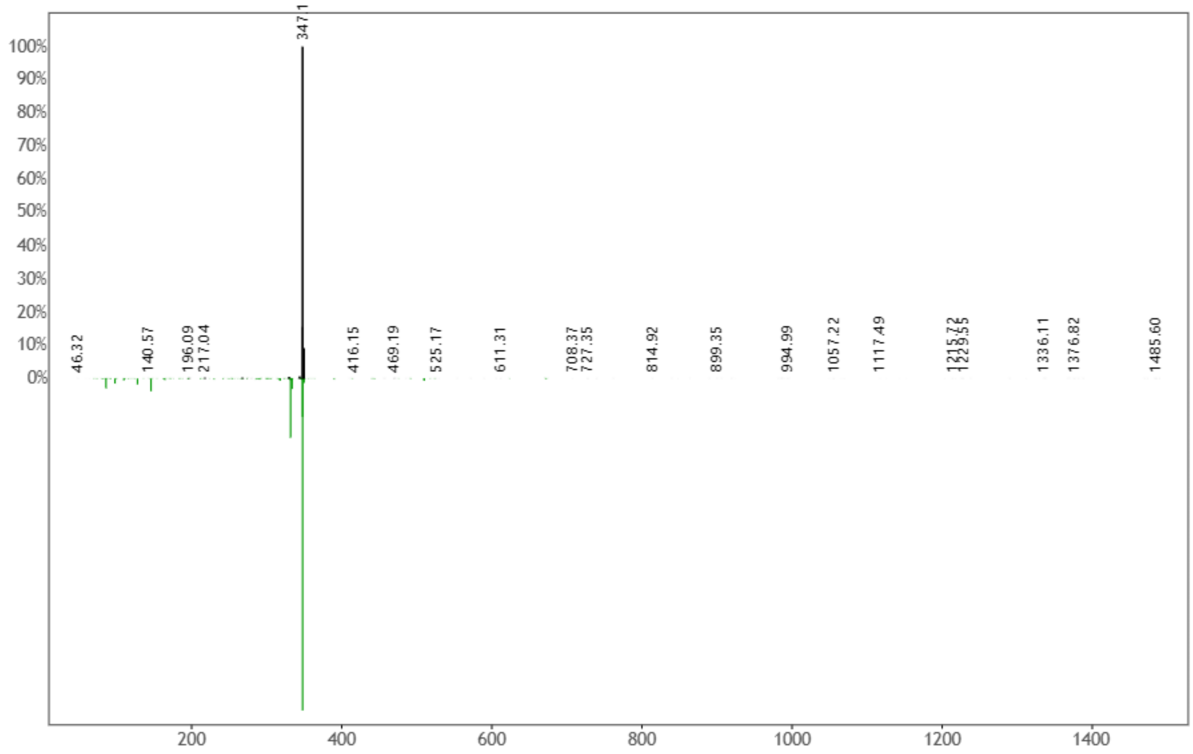

Figure S57. MS/MS spectrum of compound **f** (black) compared with GNPS library spectrum (green).

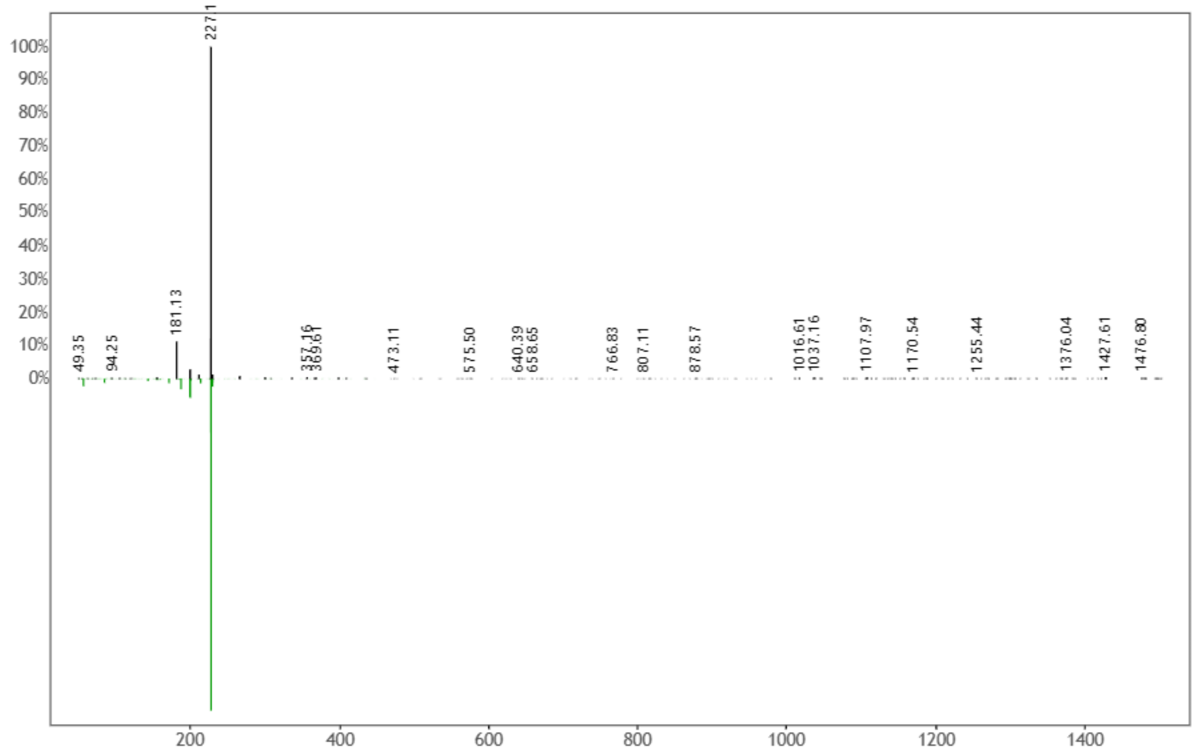

Figure S58. MS/MS spectrum of compound **g** (black) compared with GNPS library spectrum (green).

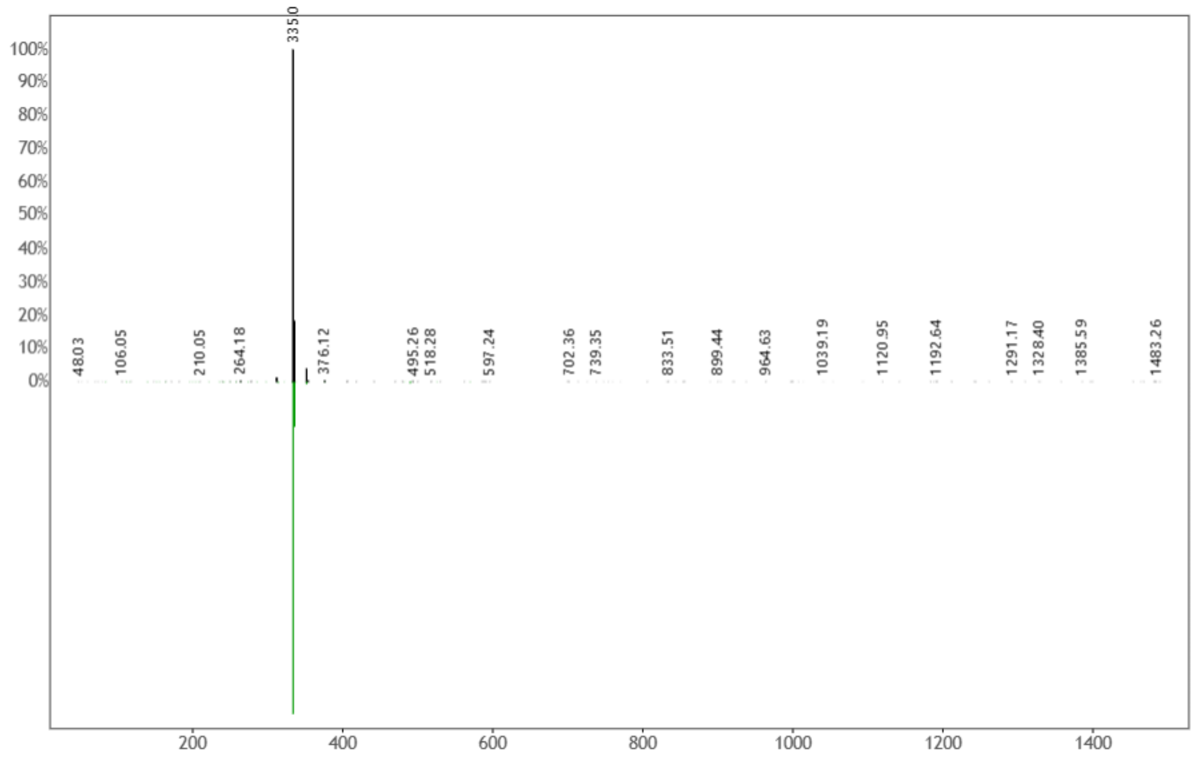

Figure S59. MS/MS spectrum of compound **h** (black) compared with GNPS library spectrum (green).

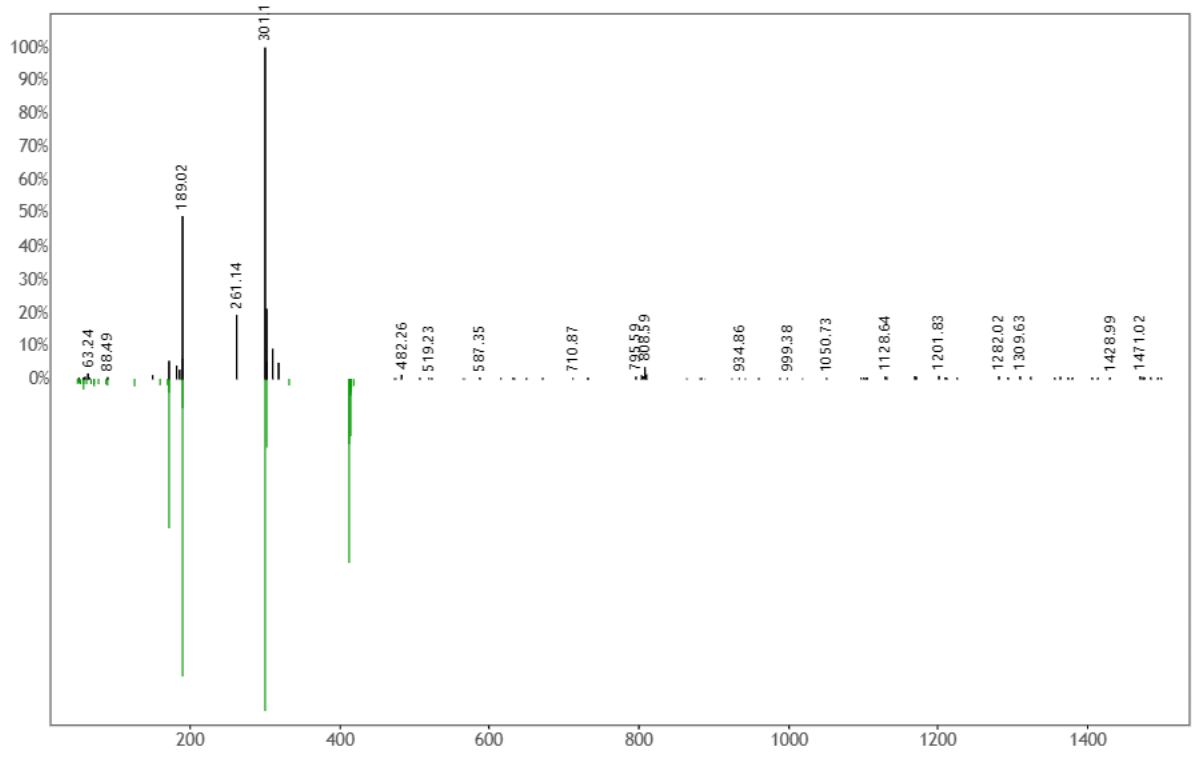

Figure S60. MS/MS spectrum of compound **i** (black) compared with GNPS library spectrum (green).

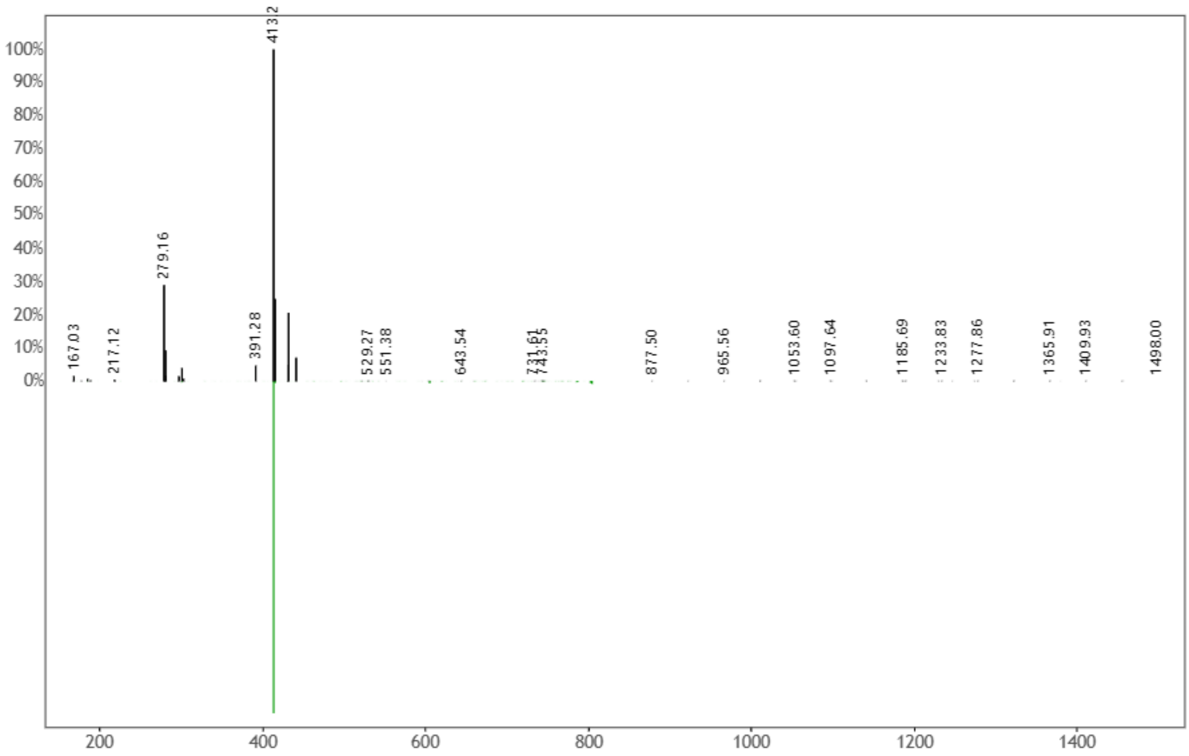

Figure S61. MS/MS spectrum of compound **j** (black) compared with GNPS library spectrum (green).

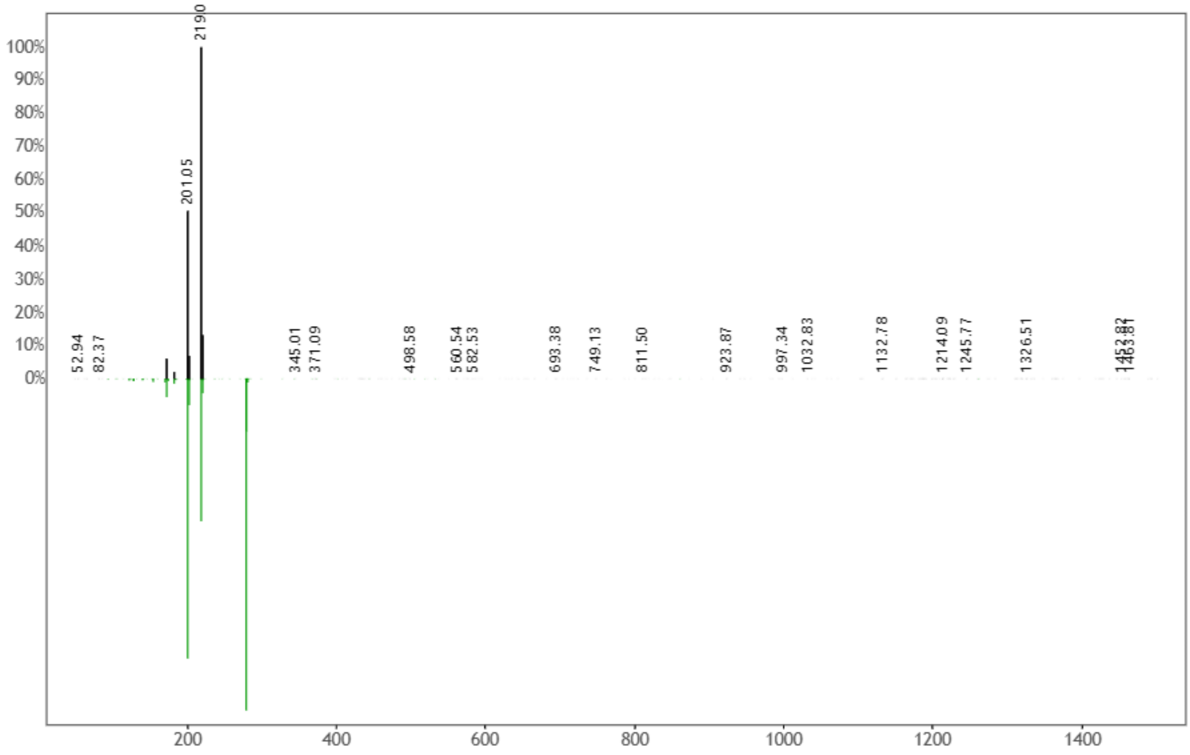

Figure S62. MS/MS spectrum of compound **k** (black) compared with GNPS library spectrum (green).

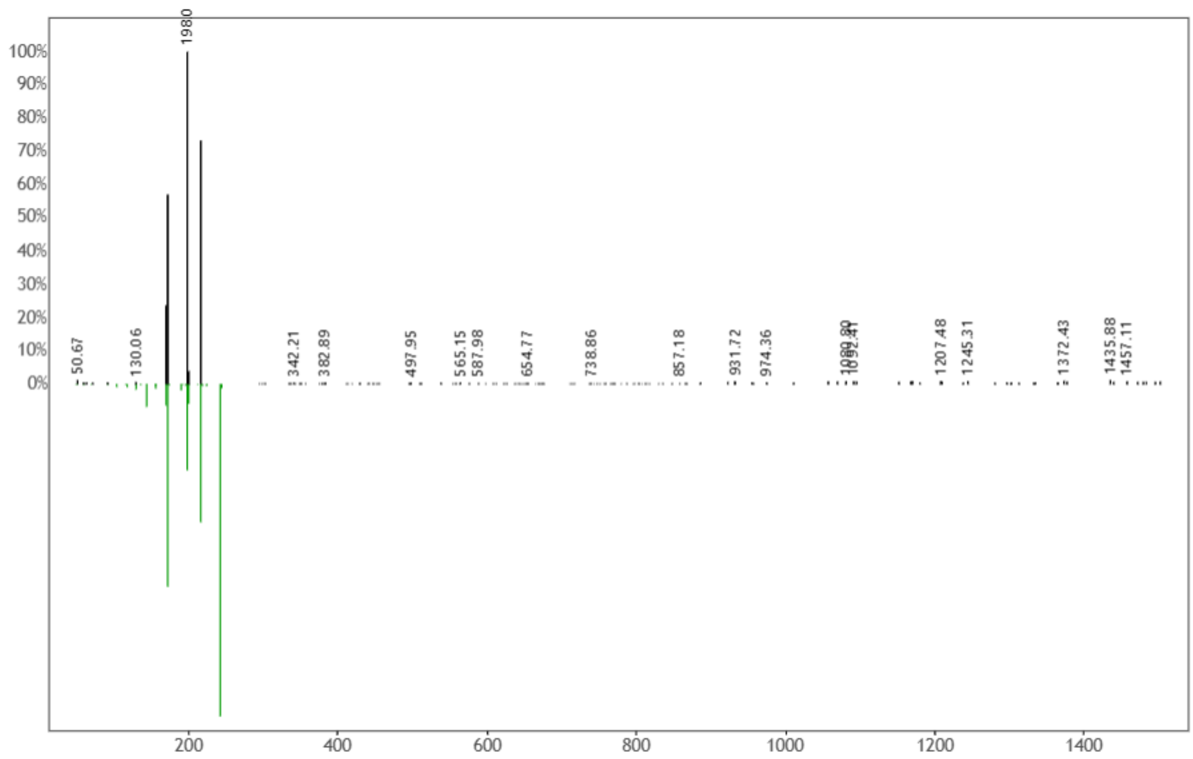

Figure S63. MS/MS spectrum of compound **l** (black) compared with GNPS library spectrum (green).

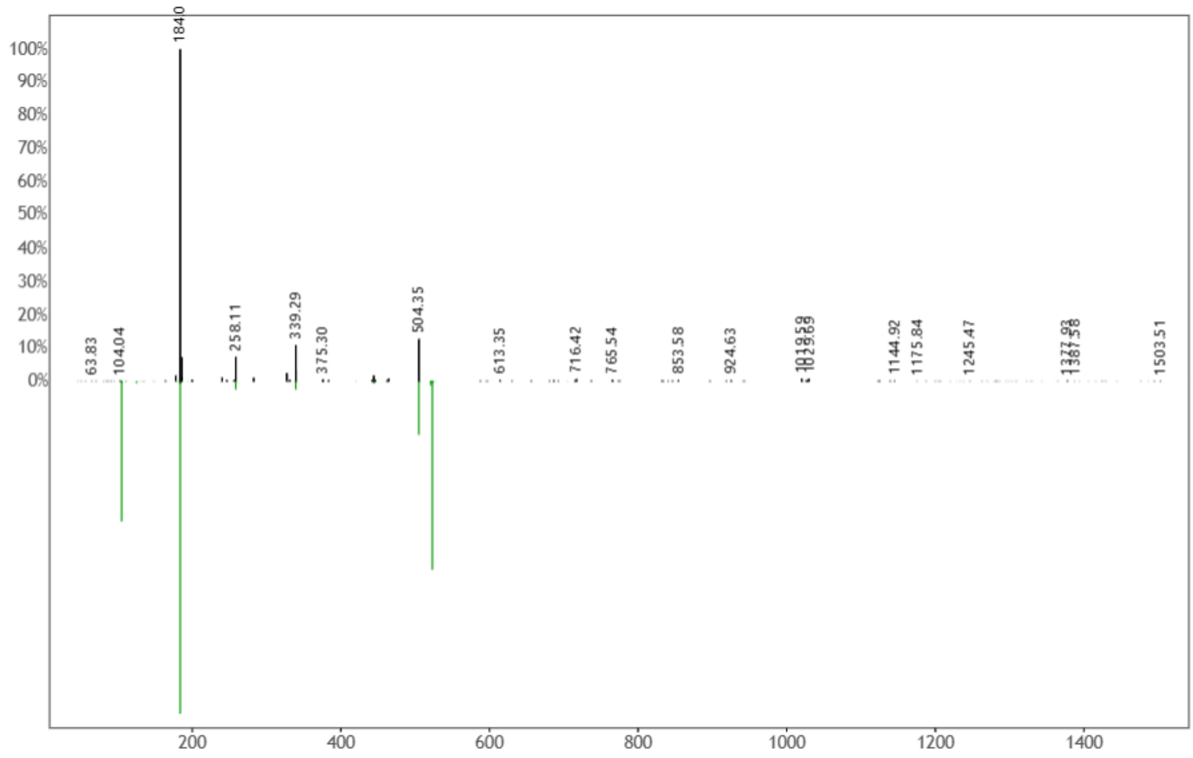

Figure S64. MS/MS spectrum of compound **m** (black) compared with GNPS library spectrum (green).

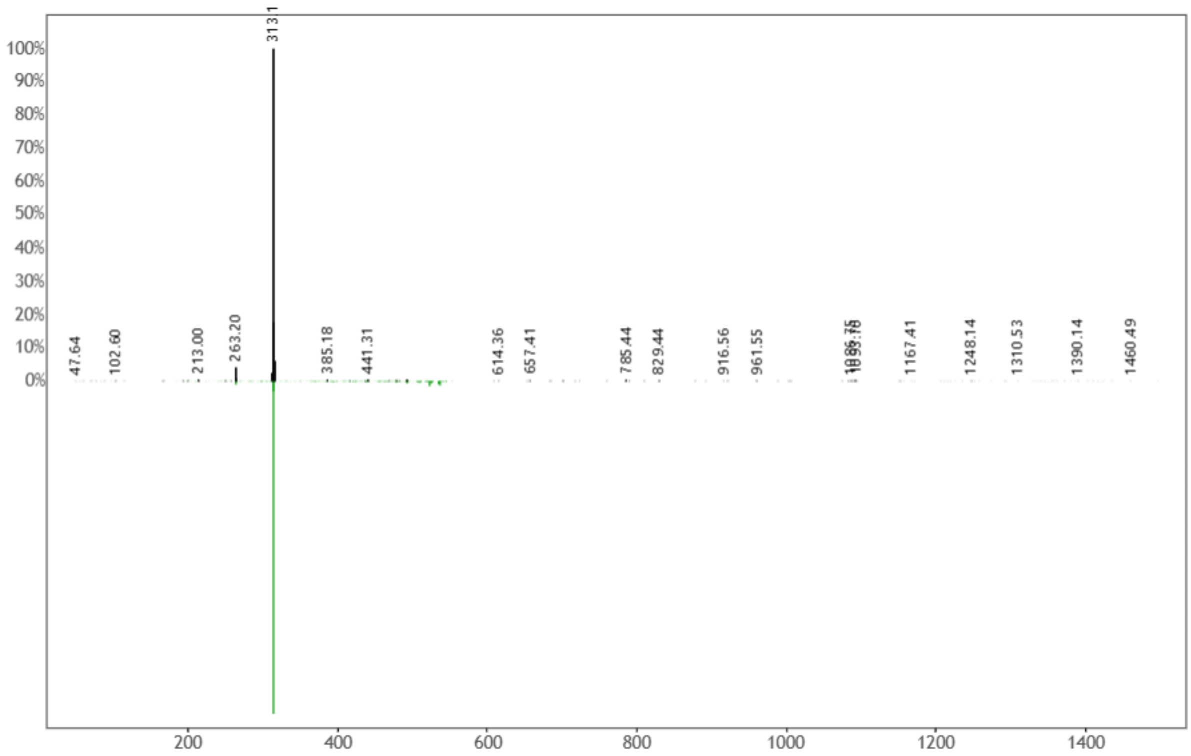

Figure S65. MS/MS spectrum of compound **n** (black) compared with GNPS library spectrum (green).

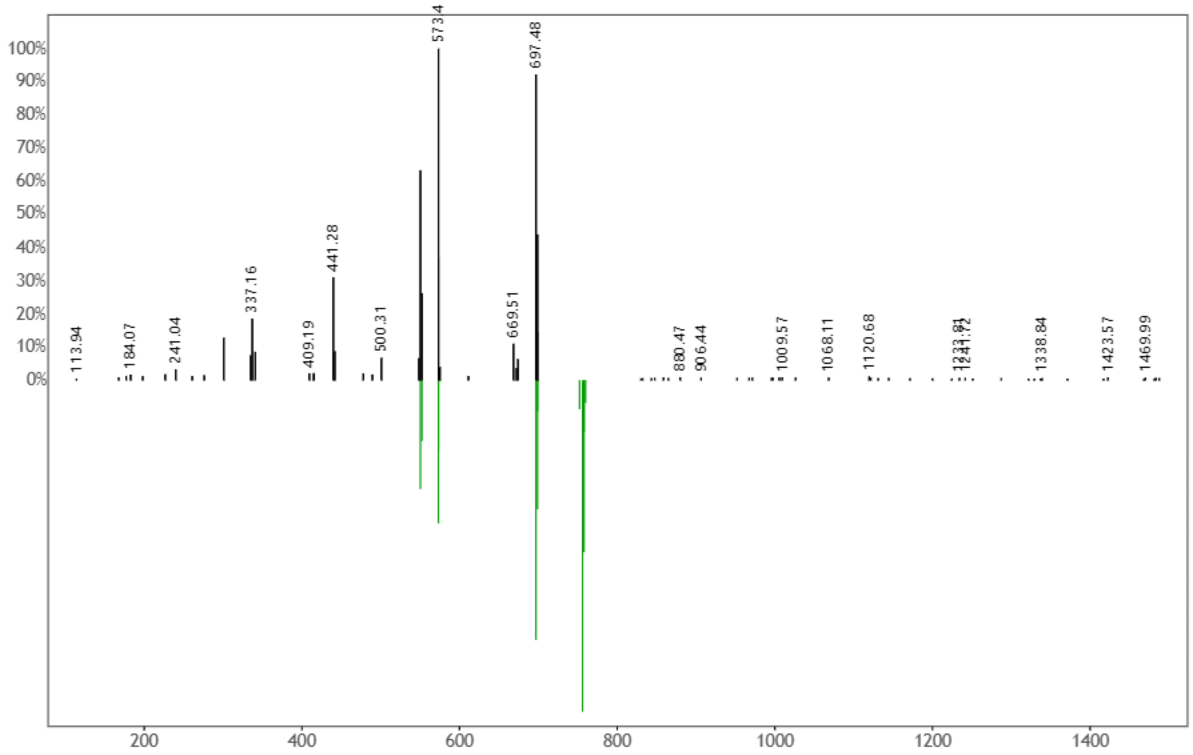

Figure S66. MS/MS spectrum of compound **o** (black) compared with GNPS library spectrum (green).

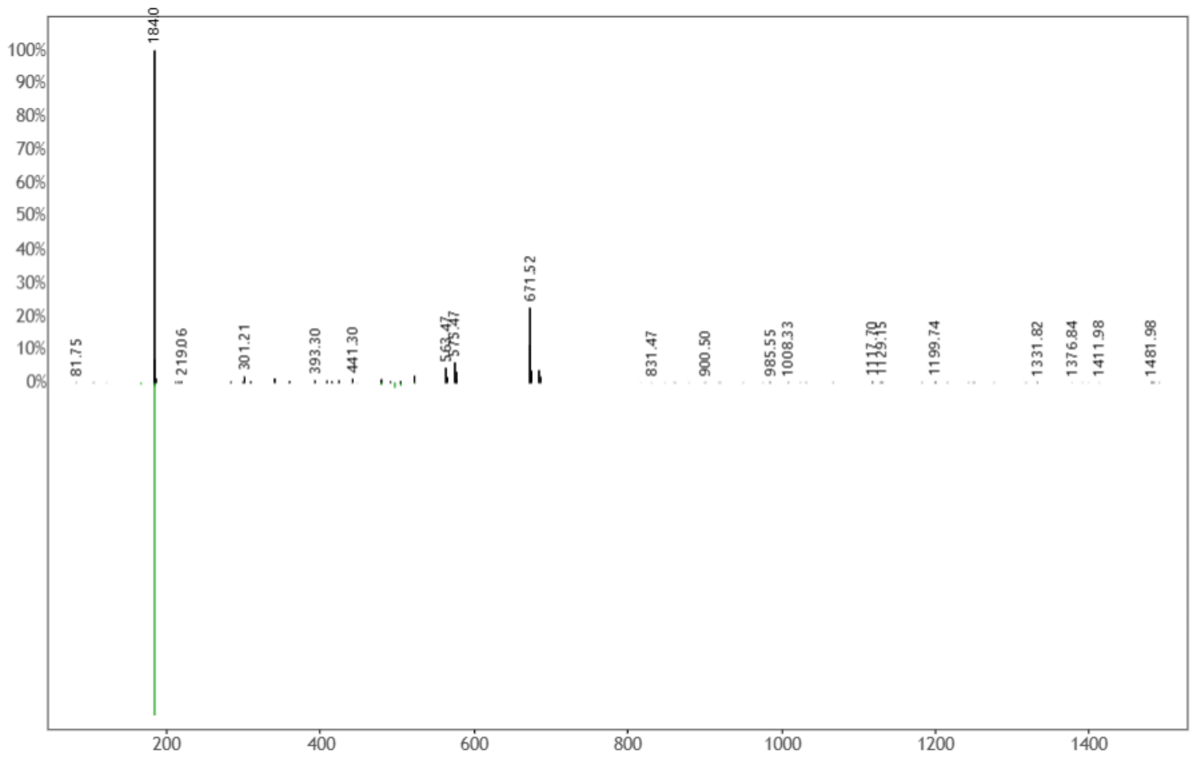

Figure S67. MS/MS spectrum of compound **p** (black, $[M+H]^+$) compared with GNPS library spectrum (green).

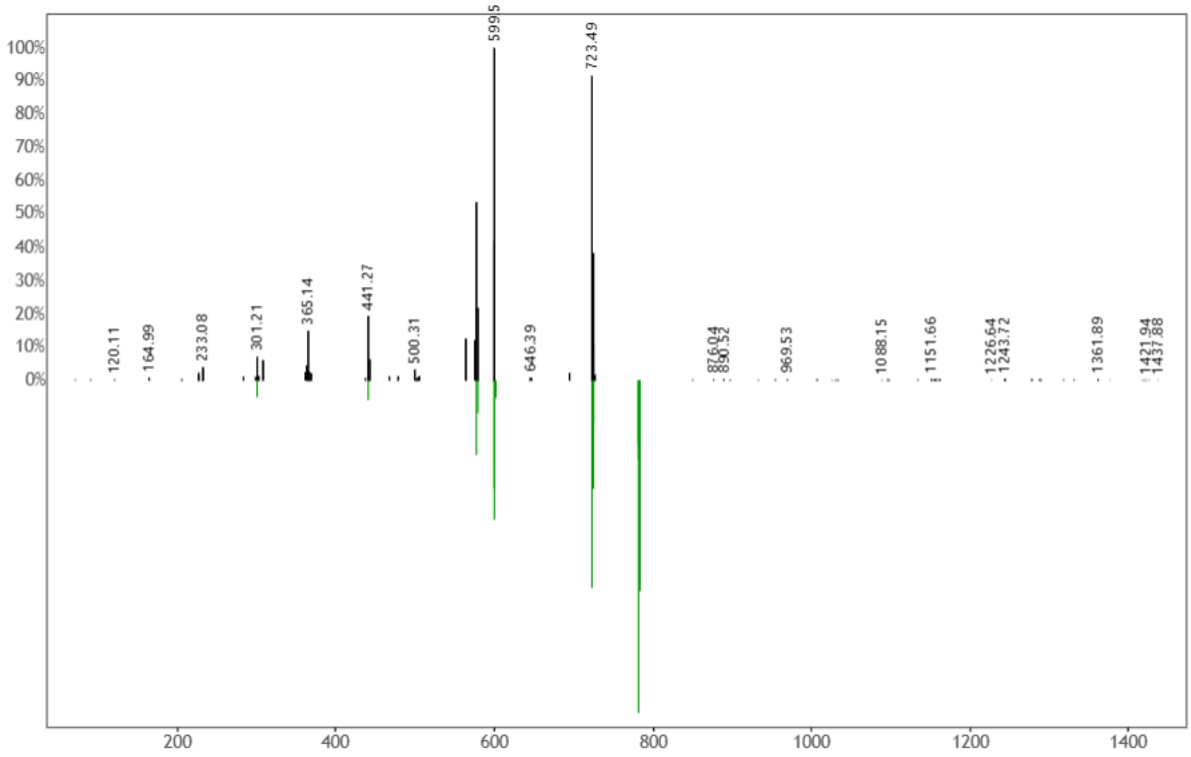

Figure S68. MS/MS spectrum of compound **p** (black, $[M+Na]^+$) compared with GNPS library spectrum (green).

Table S1. NMR spectroscopic data (400/100 MHz) of daidzein (**2**) in DMSO-d_6.

Position	δ_C	δ_H (J in Hz)
2	152.4, CH	8.20, s
3	123.3, C	
4	174.6, C	
5	127.0, CH	7.87, d (8.8)
6	115.0, CH	6.82, dd (8.8, 2.2)
7	158.9, C	
8	102.0, CH	6.69, d (2.2)
9	158.0, C	
10	116.5, C	
1’	122.9, C	
2’	130.1, CH	7.36, d (8.6)
3’	115.0, CH	6.79, d (8.6)
4’	157.0, C	
5’	115.0, CH	6.79, d (8.6)
6’	130.1, CH	7.36, d (8.6)

Table S2. ^{1}H NMR spectroscopic data (400 MHz) of compounds **3**–**8** in $CDCl_3$.

No.	3	4	5 [a]	6	7	8 [a]
3	3.76–3.70, m; 3.55–3.46, m	3.67, dt (12.0, 4.1); 3.49, ddd (12.0, 8.7, 2.8)	3.67, dd (13.1, 5.1); 3.28–3.26, m	3.67–3.61, m; 3.61–3.42, m	3.56, dt (12.1. 8.4); 3.33, ddd (12.1, 9.1, 2.9)	3.63, dd (12.8, 4.3); 3.41, dd (12.8, 1.6)
4	2.10–1.97, m; 1.95–1.78, m	1.96–1.81, m	4.24, t (4.6)	2.05–1.95, m; 1.95–1.79, m	1.77–1.57, m	4.44, t (4.3)
5	2.46–2.33, m; 1.95–1.78, m	2.47–2.31, m; 1.96–1.81, m	2.03, ddd (13.0, 5.9, 1.3); 1.32, ddd (13.0, 11.8, 4.6)	2.29, td (6.4, 3.6); 1.95–1.79, m	2.19–2.04, m; 1.95–1.82, m	2.25, ddt (13.3, 6.5, 1.6); 2.06, ddd (13.3, 11.2, 4.3)
6	4.07, dd (10.1, 5.6)	4.07, dd (9.9, 6.4)	4.33, ddd (11.8, 5.9, 2.0)	4.05, ddd (8.7, 6.6, 1.7)	2.73, dd (10.6, 6.4)	4.50, ddd (11.2, 6.5, 1.6)
8-NH				5.81, br s		
9	3.70–3.62, m	3.77, dd (5.7, 3.9)	4.45, td (5.1, 1.8)	4.26, dd (10.7, 3.0)	4.24–4.20, m	4.15, ddd (6.6, 4.4, 1.8)
10	2.24–2.16, m	2.15–1.96, m	3.13, dd (5.1, 1.8)	2.78, dd (14.5, 10.7); 3.61–3.42, m	3.16, dd (13.6, 5.8); 3.02, dd (13.6, 4.3)	1.95–1.80, m; 1.56–1.42, m
11	1.03, dd (7.1, 4.0)	1.54, ddd (13.2, 7.4, 4.0); 1.31–1.12, m				1.95–1.80, m
12	0.97, dd (6.9, 4.2)	0.90, t (7.4)				0.94, d (2.4)
13						0.93, d (2.3)
4-Me		0.99, d (6.9)				
2’			7.27–7.18, m	7.36–7.18, m	7.28–7.15, m	
3’			7.27–7.18, m	7.36–7.18, m	7.28–7.15, m	
4’			7.27–7.18, m	7.36–7.18, m	7.28–7.15, m	

5’		7.27–7.18, m	7.36–7.18, m	7.28–7.15, m
6’		7.27–7.18, m	7.36–7.18, m	7.28–7.15, m

a: NMR spectrum measured in CD_3OD

Table S3. ^{13}C NMR spectroscopic data (100 MHz) of compounds **3**–**8** in $CDCl_3$.

No.	3	4	5 [a]	6	7	8 [a]
1	169.9, C	169.8, C	166.9, C	169.6, C	165.0, CH	168.8, C
3	45.7, CH_2	45.7, CH_2	55.0, CH_2	45.5, CH_2	45.1, CH_2	54.3, CH_2
4	22.0, CH_2	39.8, CH	68.3, CH	22.6, CH_2	21.7, CH_2	68.9, CH
5	29.5, CH_2	29.5, CH_2	37.8, CH_2	28.4, CH_2	29.0, CH_2	37.9, CH_2
6	58.4, CH	58.5, CH	58.1, CH	59.2, CH	57.8, CH	58.5, CH
7	165.5, C	165.5, C	171.0, C	165.2, C	169.8, C	172.9, C
9	63.5, CH	62.9, CH	57.4, CH	56.3, CH	58.9, CH	54.9, CH
10	33.3, CH	22.1, CH_2	38.6, CH_2	36.8, CH_2	40.5, CH_2	39.1, CH_2
11	19.1, CH_3	24.6, CH_2				25.5, CH
12	17.7, CH_3	11.4, CH_3				22.0, CH_3
13						23.1, CH_3
4-Me		15.4, CH_3				
1’			137.2, C	136.1, C	135.4, C	
2’			130.8, CH	129.33, CH	130.1, CH	
3’			129.3, CH	129.27, CH	128.7, CH	
4’			127.9, CH	127.62, CH	127.6, CH	
5’			129.3, CH	129.27, CH	128.7, CH	
6’			130.8, CH	129.33, CH	130.1, CH	

a: NMR spectrum measured in CD_3OD

Table S4. Specific OR of cyclic dipeptides **3**–**8** in MeOH.

Compounds	Natural $[\alpha]_D^{20}$ (0.033, MeOH)	Literature
**3**	+147.15°	$[\alpha]_D^{25}$ +193° (0.3, MeOH) [30]
**4**	+141.14°	$[\alpha]_D^{20}$ +128° (0.1, EtOH) [31]
**5**	–174.17°	$[\alpha]_D^{20}$ –48.1° (0.1, MeOH) [32]
**6**	–168.17°	$[\alpha]_D^{25}$ –60.6° (0.66, EtOH) [33]
**7**	+114.11°	$[\alpha]_D^{20}$ +67.0° (MeOH) [34]
**8**	–282.28°	$[\alpha]_D$ –77.8° (0.38, MeOH) [35]

Table S5. NMR spectroscopic data (400/100 MHz) of compounds **9**–**12**.

No.	9 [a] δ_C	9 δ_H	10 [a] δ_C	10 δ_H	11 [b] δ_C	11 δ_H	12 [c] δ_C	12 δ_H
1					194.7, C		109.7, C	
2	151.0, C		151.6, C		128.1, C		151.2, C	
3					132.7, CH	8.18, d (2.8)	116.6, CH	6.67, dd (7.1, 1.0)
4	163.5, C		164.4, C		131.2 C		135.2, CH	7.30, ddd (8.3, 7.1, 1.6)
5	127.5, CH	7.87, dd (8.3, 1.6)	100.3, CH	5.44, dd (7.4, 1.9)	121.3, CH	8.21, ddd (6.6, 2.8, 1.4)	116.9, CH	6.66, dd (8.3, 1.0)

6	122.7, CH	7.17–7.13, m	142.3, CH	7.39, dd (7.4, 5.6)	121.9, CH	7.24–7.18, m	132.3, CH	7.92, dd (8.3, 1.6)
7	135.4, CH	7.62, ddd (8.5, 7.3, 1.6)			123.0, CH	7.24–7.18, m	173.6, C	
8	116.0, CH	7.17–7.13, m			111.6, CH	7.44, dd (6.5, 2.3)		
9	141.8, C				113.5, C			
10	114.9, C				64.9, CH_2	4.72, d (2.8)		
1-NH				10.83, br s				
3-NH		11.23, br s		11.03, br s				

a: NMR spectrum measured in DMSO-d_6; b: NMR spectrum measured in CD_3OD; c: NMR spectrum measured in $CDCl_3$
